# Synthesis of 2-Aryl-4-aminoquinazolines: Design, Molecular Docking, and In Vitro Assessment of Antibacterial and Cytotoxic Potential

**DOI:** 10.3390/ijms27062529

**Published:** 2026-03-10

**Authors:** Felipe Verdugo, Capucine Braillon, Sana Mahjoub, Alejandro Castro-Alvarez, Régine Janel-Bintz, Pierre Fechter, Pascal Villa, Claudio A. Jiménez, Diego A. Donoso-Ruiz, Marcia Pérez-Fehrmann, Víctor Kesternich, Sergio Ortiz, Ronald Nelson

**Affiliations:** 1 Departamento de Química Orgánica, Facultad de Ciencias Químicas, Universidad de Concepción, Edmundo Larenas 129, Concepción 4070371, Chile; felipeverdugo@udec.cl (F.V.); cjimenez@udec.cl (C.A.J.); ddonoso2018@udec.cl (D.A.D.-R.); 2 CNRS, UMR 7242, Biotechnologie et Signalisation Cellulaire, Institut de Recherche de l’Ecole de Biotechnologie de Strasbourg, Université de Strasbourg, 67400 Illkirch-Graffenstaden, France; capucine.braillon@etu.unistra.fr (C.B.); sanamahjoub0903@gmail.com (S.M.); regine.janel@unistra.fr (R.J.-B.); p.fechter@unistra.fr (P.F.); 3 UMR 7200 Laboratoire d’Innovation Thérapeutique, CNRS, Strasbourg Drug Discovery and Development Institute (IMS), Université de Strasbourg, 67400 Illkirch-Graffenstaden, France; 4 Laboratory of Human Genome and Multifactorial Diseases (LR12ES07), Faculty of Pharmacy, University of Monastir, Monastir 5000, Tunisia; 5 Departamento de Ciencias Preclínicas, Facultad de Medicina, Universidad de La Frontera, Temuco 4811230, Chile; alejandro.castro.a@ufrontera.cl; 6 Millennium Nucleus Bioproducts, Genomics and Environmental Microbiology (BioGEM), Valparaíso 2390123, Chile; 7 PCBIS Plateforme de Chimie Biologie Intégrative de Strasbourg, UAR 3286 CNRS/Université de Strasbourg, 67000 Strasbourg, France; pvilla@unistra.fr; 8 Departamento de Química, Facultad de Ciencias, Universidad Católica del Norte, Avda. Angamos 0610, Antofagasta 1270709, Chile; maperez@ucn.cl (M.P.-F.); vkestern@ucn.cl (V.K.)

**Keywords:** aminoquinazolines, antibacterial activity, molecular docking studies

## Abstract

Antimicrobial resistance (AMR) remains a major threat to modern medicine, fueled by the excessive use of antibiotics and the spread of multidrug-resistant pathogens such as methicillin-resistant *Staphylococcus aureus* (MRSA). In this study, we designed and synthesized a series of 2-aryl-4-aminoquinazoline derivatives bearing an aminoalkylimidazole linker, combining two pharmacophoric motifs associated with antimicrobial activity. Starting from anthranilamide, the compounds were prepared in three straightforward steps, affording good yields and high purity. Their structures were confirmed by FT-IR spectroscopy, ^1^H and ^13^C nuclear magnetic resonance (NMR), and high-resolution mass spectrometry (HRMS). Biological evaluation showed that series **5** exhibited strong selectivity toward *S. aureus*, with compounds **5c** and **5d** displaying minimum inhibitory concentrations (MICs) between 2.2 and 4.4 µM. No significant activity was observed against other tested strains. Cytotoxicity assays in HepG2 cells revealed moderate to low inhibition. Molecular docking indicated preferential binding to dihydrofolate reductase (DHFR) and relevant interactions with topoisomerase IV, resembling reference inhibitors. ADME analysis predicted favourable absorption, blood–brain barrier permeability, and compliance with Lipinski’s rules.

## 1. Introduction

The accelerating spread of antimicrobial resistance (AMR) has become a major threat to global public health, severely compromising the efficacy of conventional antibiotics [1]. The widespread and often inappropriate use of antimicrobial agents in both clinical and agricultural settings has contributed to the emergence of multidrug-resistant (MDR) bacterial strains, such as methicillin-resistant *Staphylococcus aureus* (MRSA) and carbapenem-resistant *Klebsiella pneumonia* [2]. *S. aureus* is one of the most significant pathogens in healthcare systems, being responsible for a wide range of infections; from minor skin conditions to severe diseases such as bacteraemia and infective endocarditis [3]. Notably, it is among the top five causes of hospital-acquired infections and a leading agent in post-surgical wound infections [4]. The emergence of MRSA has further complicated treatment, leading to increased morbidity and mortality rates [5]. In 2019, *S. aureus* was associated with over 1 million deaths globally, underscoring its critical impact on public health. Various antimicrobial strategies have been recently explored, including the use of nanoparticles [6,7,8,9], natural products and extracts [10,11,12], ions and metallic compounds [13,14,15,16], and hybrid compounds and materials [17,18,19,20]. While these approaches exhibit promising antibacterial activity, they may suffer from limitations related to complexation, selectivity, or translational challenges. In contrast, small-molecule scaffolds remain highly attractive due to their synthetic accessibility, tunable pharmacological properties, and suitability for classical medicinal chemistry optimization. Importantly, the development of new antibacterial compounds with enhanced selectivity toward specific bacterial species or strains is urgently needed to improve therapeutic efficacy, preserve host microbiota, and reduce selective pressure for resistance [21,22].

Among the diverse chemical scaffolds explored for antibacterial drug development, the quinazoline core has attracted considerable interest due to its broad biological activity palette, including anticancer, anti-inflammatory, and antimicrobial properties [23]. In recent years, they have demonstrated promising antibacterial activity, particularly when bearing amino or substituted heterocyclic moieties [24]. Notably, 2,4-disubstituted quinazolines have shown strong in vitro activity against Gram-positive bacteria such as *S. aureus* and *Mycobacterium smegmatis*, with structure–activity relationship (SAR) studies suggesting that both, electron-withdrawing groups and heteroatom substitutions, enhance their potency [25]. These findings highlight the versatility of the quinazoline core as a scaffold for designing novel agents able to overcome existing resistance mechanisms.

To gain a better understanding of the interaction mechanisms and improve the drug-like properties of these compounds, computational approaches have become fundamental to the early-stage discovery of antibacterial drugs. In silico tools such as molecular docking, pharmacophore modelling, and quantitative structure–activity relationship (QSAR) analyses enable rapid screening and optimization, predicting key structural features that influence target binding and biological activity [26]. Moreover, the early evaluation of ADME-Tox (absorption, distribution, metabolism, excretion and toxicity) properties and drug-likeness through platforms such as SwissADME or pkCSM facilitates the selection of lead candidates with favourable pharmacokinetic profiles [27]. Recent studies have demonstrated the utility of such computational workflows in identifying quinazoline-based compounds with high target affinity and selectivity toward bacterial enzymes, supporting their further development as antibacterial agents [28].

**Scheme 1:**
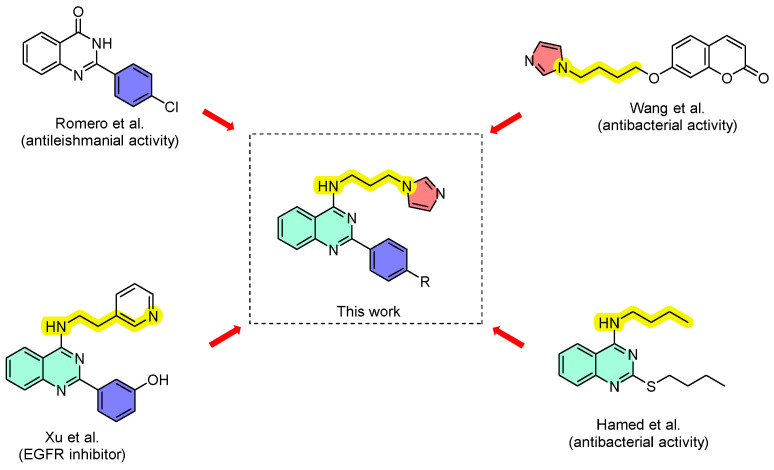
Design of a new 2-aryl-4-aminoquinazoline [31,33,36,42].

In this context and based on the continuous need for new antibacterial agents, we hypothesized that the combination of 2-aryl-4-aminoquinazolines with imidazoles, a well-established pharmacophoric scaffold extensively documented in the literature [29,30,31,32], could yield novel compounds with significant antimicrobial potential. Although 4-aminoquinazoline derivatives have been investigated for their anticancer properties [33,34,35], and 2-arylquinazolinones as antiparasitic [36], antibacterial [37,38,39,40,41] and antifungal agents [42], we sought to evaluate a hybrid system based on 2-aryl-4-aminoquinazolines and aminoalkylimidazoles, the latter having been described by Wang et al. as antiproliferative agent through the inhibition of EGFR receptors (Scheme 1) [35]. Herein, we report the synthesis, in vitro antibacterial evaluation, molecular docking with DHFR and topoisomerase IV and ADME prediction of new 2-aryl-4-aminoquinazolines.

## 2. Results and Discussion

### 2.1. Chemistry

**Scheme 2:**
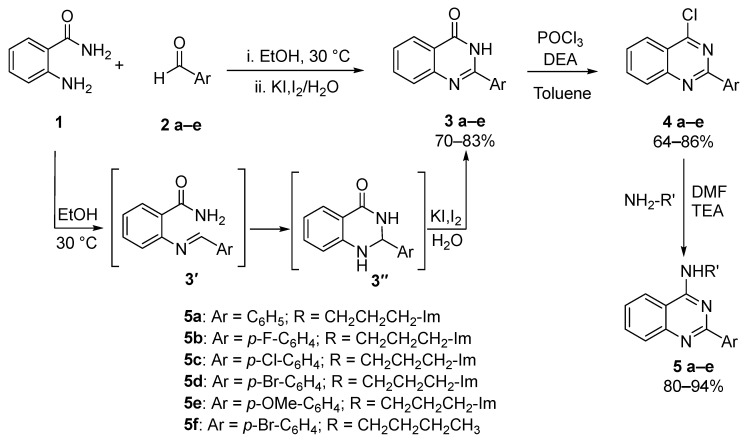
Preparation of 2-aryl-4-aminequinazolines. Im = imidazole.

The synthesis of the designed 2-aryl-4-aminoquinazoline derivatives was conducted through a concise, efficient and scalable three-step sequence, starting from commercially available anthranilamide (**1**) as depicted in Scheme 2. This starting material was chosen due to its versatility and broad utility in heterocyclic synthesis. In the first step, a series of 2-arylquinazolinones (**3a**–**e**) were synthesized via a cyclocondensation reaction between anthranilamide (**1**) and various substituted benzaldehydes (**2a**–**e**). The transformation comprises a condensation/cyclization/oxidation sequence, achieved through a *one-pot* addition of reagents at room temperature under air. Mechanistically significant, Bakavoli et al. [31] identified imine (**3′**) and dihydroquinazolinone (**3″**) as key intermediates, both formed by mixing **1** and **2**(**a**–**e**) in ethanol at room temperature. This latter intermediate is then oxidized by the direct addition of an KI/I_2_ aqueous solution, yielding the corresponding quinazolinone derivatives in good to excellent yields. The choice of the mild KI/I_2_ oxidizing solution is particularly advantageous due to its operational simplicity, functional group tolerance and metal-free conditions. In the second step, the conversion of the 2-arylquinazolinones (**3a**–**e**) into the corresponding 4-chloroquinazolines (**4a**–**e**) was achieved with phosphorus oxychloride (POCl_3_) in the presence of *N,N*-diethylaniline, which acted both as base and catalyst. The reactions were conducted in dry toluene under reflux, following protocols reported in the literature [35], affording the chlorinated intermediates in moderate to high yields, ranging from 63 to 92%. This transformation is shown to be essential since the 4-chloro substituent behaves as an excellent leaving group in the subsequent nucleophilic aromatic substitution step. In the final step, key 2-aryl-4-aminoquinazoline derivatives (**5a**–**e**) were synthesized through a nucleophilic aromatic substitution reaction (S_N_Ar) between **4**(**a**–**e**) and an excess of 1-(3-aminopropyl)imidazole, in the presence of triethylamine as base employing DMF as solvent at room temperature. This methodology, adapted from procedures reported by Okano et al. and Reddy et al. [43,44], proved to be highly efficient, affording the desired 4-aminoquinazoline products in excellent yields ranging from 85 to 94%. The use of 1-(3-aminopropyl)imidazole introduces a polar, biologically relevant imidazole moiety, able to enhance the product solubility in aqueous media while increasing potential interactions with biological targets.

**Figure 1:**
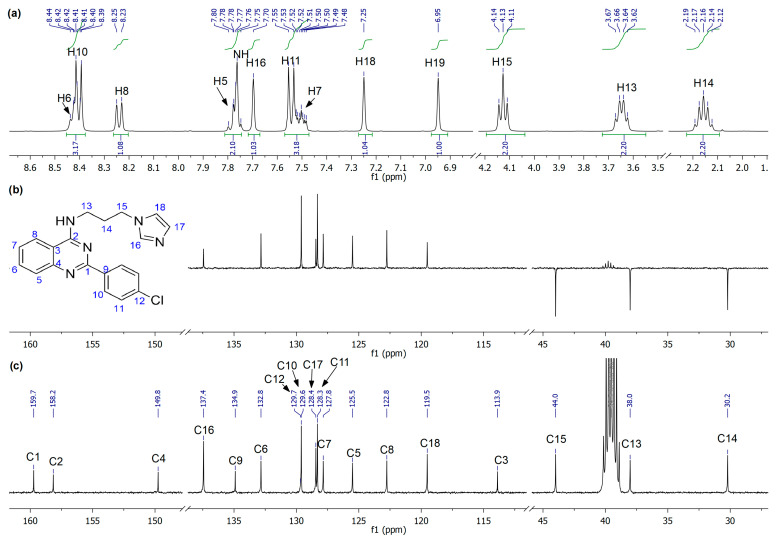
NMR spectra of compound **5c**. (**a**) ^1^H-NMR, (**b**) DEPT-135, (**c**) ^13^C-NMR.

All synthesized compounds were fully characterized by FT-IR, ^1^H- and ^13^C-NMR spectroscopy, and high-resolution ESI mass spectrometry (HRMS-ESI). In the ^1^H-NMR spectra, resonances from both aliphatic and aromatic environments can be rapidly differentiated. As representative example, the compound **5c** (Figure 1a) shows clean and well-resolved signals, allowing a complete assignment from combined mono- and bidimensional NMR analysis. In particular, signals from the alkyl-imidazole fragment can be readily recognized: a pentet at δ 2.16 ppm (-CH_2_-, H14), a quartet at δ 3.65 ppm (NH-CH_2_, H13), and a triplet at δ 4.13 ppm (Im-CH_2_, H15). The secondary amine is observed as a multiplet centered at δ 7.77 ppm (NH), while the protons at the imidazole ring exhibit three broad singlets at δ 6.95 (H19), 7.25 (H18), and 7.70 ppm (H16), consistent with non-equivalent Csp^2^–H environments. Furthermore, the integration of the remaining signals between δ 7.45 and 8.50 ppm corresponds to nine protons, which can be associated with four protons of the *p*-substituted phenyl ring, four from the quinazolinone ring, and the NH of the secondary amine. Complementary ^13^C-NMR and DEPT-135 experiments (Figure 1b,c) enabled the assignment of the carbon framework: three methylenes (CH_2_) at δ 30.2–44.0 ppm; aromatic carbons appearing from δ 113.9 ppm with imidazole CH carbons at δ 119.5 (C18), 128.4 (C17), and 137.4 ppm (C16); six CH signals from the quinazolinone precursor and *p*-substituted phenyl rings; and six additional quaternary carbons at δ 113.9 (C3), 129.7 (C12), 134.9 (C9), 149.8 (C4), 158.2 (C2), and 159.7 ppm (C1).

### 2.2. Biological Studies

The antibacterial activity of the synthesized compounds was assessed through a three-step experimental approach. Initially, compounds from series **3**, **4**, and **5** were screened using an agar diffusion assay against two bacterial model strains, aiming to identify those series exhibiting antibacterial potential. Subsequently, the active series were further evaluated against a panel of 50 reference and clinically isolated bacterial strains using an agar dilution method aiming to determine their minimum inhibitory concentrations (MICs). In the third phase, the antibacterial activity was confirmed via a broth dilution assay conducted on selected bacterial strains. Finally, the cytotoxic potential of the active compounds was assessed by measuring their antiproliferative effects on the HepG2 human cell line, thereby evaluating their impact on eukaryotic cells.

The antibacterial evaluation using agar diffusion assays allowed the determination that series **5** is active against both tested strains (*Staphylococcus aureus* and *Pseudomonas aeruginosa*) at concentrations ranging from 200 to 50 µg/mL (Table 1). Among the series, compound **5c** exhibited the most pronounced antibacterial activity, producing inhibition diameters of 19.5 mm against *S. aureus* and 32.5 mm against *P. aeruginosa* at 500 µg/mL, results that are comparable to those obtained with the positive control, ciprofloxacin. Compounds **5b** and **5d** also displayed significant antibacterial effects against both strains across the tested concentrations, with inhibition diameters ranging from 8 to 30 mm. In contrast, compounds **5a** and **5e** showed a detectable activity only at the highest concentration tested (200 µg/mL), with inhibition diameters of 9–11 mm for *S. aureus* and 18–21 mm for *P. aeruginosa*. Notably, compounds from series **3** and **4** did not exhibit antibacterial activity under the tested conditions. These results highlight the importance of substitution at position 4 of the 2-aryl-quinazoline scaffold in modulating in vitro antibacterial activity. While 2-aryl-quinazoline derivatives have been reported in the literature to exhibit intrinsic antibacterial properties, our data indicates that, within the present series, antibacterial activity is observed only when this scaffold is combined with an appropriate substituent at position 4. This suggests that both the 2-aryl-quinazoline core and the substitution at position 4 are required to achieve sufficient interaction with the potential bacterial targets.

Subsequently, compounds (**5a**–**e**) were evaluated for their antibacterial activity using the agar dilution method in order to determine their minimum inhibitory concentrations (MICs) (Table 2). Activity was observed exclusively against *Staphylococcus* species. Among the tested compounds, **5c** and **5d** exhibited the most significant antibacterial effects against both reference and clinically isolated strains of *S. aureus*, with MIC values ranging from 12.2 to 27.5 µM. Compounds **5e** and **5a** demonstrated moderate activity against the same strains, with MIC values ranging from 27.8 to 60.7 µM, while compound **5b** showed no significant antibacterial activity. The lower antibacterial activity observed for the fluoro-substituted derivative compared to its chloro- and bromo- analogues may be attributed to the smaller size and lower polarizability of fluorine, which can limit hydrophobic and van der Waals interactions with potential bacterial targets. Notably, the compounds displayed a considerably lower efficacy against other *Staphylococcus* species, including *S. epidermidis*, *S. haemolyticus*, *S. lugdunensis*, and *S. saprophyticus*, with MIC values ranging from 122.5 to 303.6 µM.

The larger inhibition zones observed against *P. aeruginosa* in the agar diffusion assay, despite the lack of activity in broth microdilution tests, likely reflect differences between the two methodologies. Agar diffusion assays are influenced by compound diffusion and local concentration gradients, while broth microdilution assays provide a more stringent assessment of antibacterial efficacy in liquid culture.

To confirm the antibacterial activity observed in the agar dilution assay for selected compounds of series **5**, a broth microdilution test was conducted against various *S. aureus* strains (Table 3). Consistent with the previous results, compounds **5c** and **5d** exhibited the most potent activity, with MIC values ranging from 2.2 to 4.4 µM. Moderate activity was also observed for compounds **5a** and **5e**, while compound **5b** showed no antibacterial effect at the tested concentrations, in agreement with the agar dilution results. To further assess the role of the imidazole moiety in modulating antibacterial activity, a derivative lacking this group (*N*-propyl-2-(*p*-bromophenyl)-quinazolin-4-amine, **5f**) was synthesized and evaluated. The resulting MIC values were comparable to those observed for the structural analogue **5d**, suggesting that the presence of the imidazole ring has a limited impact on the antibacterial activity within this series.

Selective antibacterial agents that specifically target *S. aureus* without affecting other *Staphylococcus* species represent a promising strategy in antimicrobial therapy. Such specificity minimizes disruption to the commensal microbiota, thereby reducing the selective pressure that often leads to the emergence of antibiotic-resistant strains. Several synthetic compounds have demonstrated selective antibacterial activity against *S. aureus* while exhibiting a limited or no activity against other *Staphylococcus* species. For instance, afabicin (Debio 1450), a prodrug converted in vivo to afabicin desphosphono, specifically inhibits the enoyl-acyl carrier protein reductase (FabI) enzyme in *S. aureus*, demonstrating an efficacy comparable to vancomycin in clinical trials and a minimal impact on the normal microbiota [45]. Similarly, novel 4-piperazinylquinoline derivative (4-(4-(2,3-dichlorobenzoyl)piperazin-1-yl)-6,7-dimethoxyquinoline-3-carbonitrile) displayed a potent activity against *S. aureus* with MIC values around 10 μM, while remaining inactive against Gram-negative bacteria like *P. aeruginosa* [46].

The evaluation of cytotoxic activity for those active compounds in the series revealed a moderate antiproliferative effect against the HepG2 human hepatocellular carcinoma cell line, relative to the reference compound camptothecin (Table 4). The half-maximal inhibitory concentration (IC_50_) values ranged from 15.7 µM for **5d** to 30.9 µM for **5a**, indicating a variable but generally mild cytotoxic profile within the series. Notably, compound **5b** exhibited no significant inhibitory activity at the tested concentrations. Selectivity indices (SI) were calculated as the ratio between cytotoxic IC_50_ values on HepG2 cells and antibacterial MIC values. Among the tested compounds, derivative **5d** showed the highest selectivity index (SI = 7.1), indicating a favorable balance between antibacterial activity and eukaryotic cell tolerance within this series.

### 2.3. Molecular Docking

Considering the inherent planarity of the quinazolines core, it was postulated as a working hypothesis that the observed biological activity could be associated with the interference with the catalytic process of two target enzymes: dihydrofolate reductase (DHFR), which is susceptible to inhibition by diaminopyrimidine derivatives such as trimethoprim and, on the other hand, topoisomerase IV (TopoIV) of *Staphylococcus aureus*, which is inhibited by third- and fourth-generation fluoroquinolones. Consequently, the interaction between the synthesized compounds and the crystallographic structure of DHFR (cocrystallized with trimethoprim) or with a model of TopoIV (generated in the presence of delafloxacin) were evaluated.

#### 2.3.1. DHFR and Synthesized Compounds

**Figure 2:**
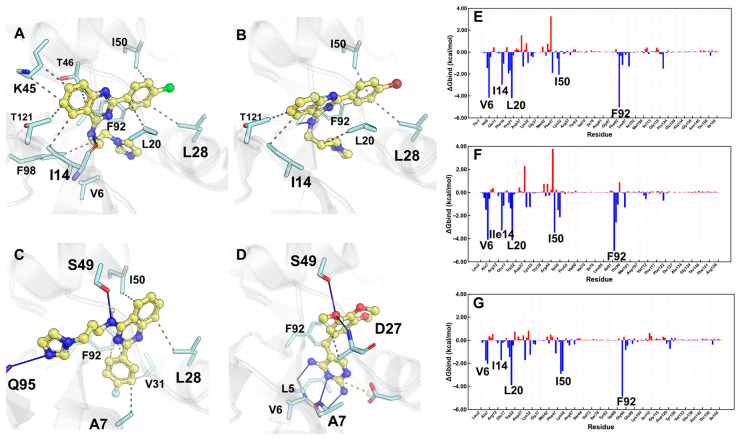
Binding modes of the evaluated compounds (**5c** (**A**), **5d** (**B**), and **5b** (**C**)) and trimethoprim (**D**) (shown in yellow) within the catalytic site of DHFR, and the most characteristic residues (shown in cyan) binding with the ligands. Plot of the energetic contributions per-residues with **5c** (**E**), **5d** (**F**), and **5b** (**G**), ordered according to the biological activity of the compounds.

Molecular docking studies were performed to elucidate the binding affinity and identify the residues involved in the orientation of the synthesized compounds within the catalytic site of DHFR. In this context, the binding affinities of compounds **5c** and **5d**, as the most active, and compound **5b**, the least active, were analyzed to elucidate the influence of halogen substitution at the *para* position of the phenyl ring on biological activity. Interestingly, molecular docking results indicated that **5c** and **5d** adopted analogous binding positions. Specifically, the imidazole fragment was oriented between residues L20 and F98, the quinazoline core was positioned between I14 and T121, and the halogenated phenyl ring was located between I50 and L28 (Figure 2A,B).

In contrast, compound **5b** exhibited a different binding position, in which the imidazole fragment interacted with Q95, the amino group of the quinazoline system interacted with S49, and the halogenated phenyl was located between residues A7, V31, and F92, orienting part of the quinazoline core between L28 and I50 (Figure 1c). Although the pose of compound **5b** suggests favorable interactions, analysis of the energy contribution per residue by MM-GBSA calculations (Figure 2C,G and Figure S1) revealed unfavorable interactions involving the fluorinated phenyl ring of **5b**, which would negatively affect its interactions with V31 and I14. It is hypothesized that the smaller atomic radius of fluorine compared to chlorine or bromine could explain these differences. Different atomic radius sizes could impose steric constraints on compounds **5c** and **5d**, preventing them from adopting a similar conformation observed for compound **5b**, resulting in a more effective inhibition of DHFR catalytic activity. A comparison between the poses of most active compounds and trimethoprim (Figure 2D) suggests that their activity is attributed to more favorable interactions, predominantly established by their diaminopyrimidine core and the trimethoxyphenyl moiety.

#### 2.3.2. TopoIV and Synthesized Compounds

**Figure 3:**
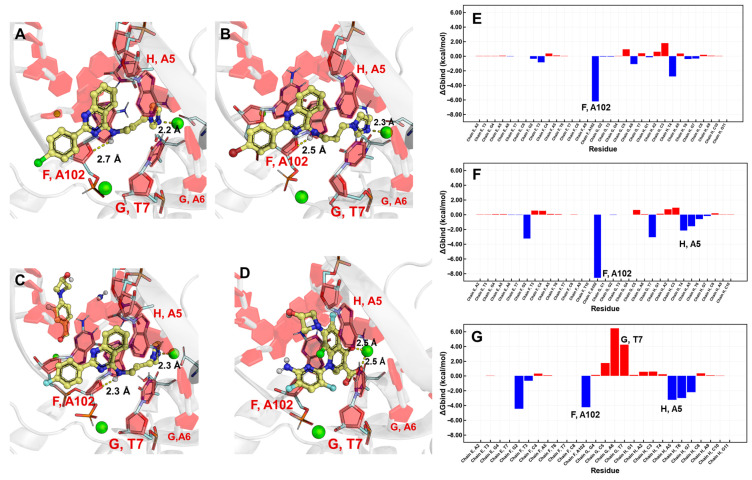
The binding mode of the synthesized compounds (**5c** (**A**), **5d** (**B**), and **5d** (**C**) in yellow) with the delafloxacin-binding site (**D**), nucleotides are represented in red and only those relevant to the interaction with ligands are represented as sticks. The plots of the energy contribution per residue represent the interactions of compounds **5c** (**E**), **5d** (**F**) and **5b** (**G**).

The Topoisomerase IV (TopoIV) model (generated using AlphaFold) was based on the crystal structure of *S. pneumoniae* TopoIV complexed with delafloxacin, a fluoroquinolone, as the primary template. The most active compounds, **5c** and **5d**, exhibited significantly higher binding energies (coupling scores) in their interactions with TopoIV. In addition, the binding mode of the synthesized compounds was similar to that observed for delafloxacin, suggesting intercalation between bacterial DNA nucleotides. Likewise, the imidazole moiety of the evaluated compounds established interactions with the magnesium ion, analogous to the mechanism reported for fluoroquinolones. The affinity energies calculated for the interaction with TopoIV were −45.15 kcal/mol for **5c** and −34.92 kcal/mol for **5d**. Consistently, compound **5b** showed a lower binding affinity for TopoIV compared with the more active analogs (**5c** and **5d**). MM-GBSA analysis enabled the determination of the individual energy contributions of bacterial DNA nucleotides to ligand binding. Nucleotide A102 of chain F was found to contribute favorably to the binding affinity for all ligands evaluated (Figure 3E–G). This nucleotide interacts with the quinazoline core through π–π interactions and hydrogen bonding (Figure 3 and Figure S2). For compounds **5c** and **5d**, the energy contribution of this specific interaction exceeded −6 kcal/mol, whereas for compound **5b** it did not exceed −5 kcal/mol. Additionally, the lower overall affinity of compound **5b** may be attributed to unfavorable interactions between its quinazoline core and the adjacent T7 and A6 nucleotides of the G chain. These unfavorable interactions are likely a consequence of steric hindrance generated by the proximity of the quinazoline core of **5b**, resulting in a perturbation of the conformation of neighboring nucleotides of A102.

Although the biological activity of the simulated compounds was consistent with the binding energies of the synthesized compounds (Table 5), the values obtained indicated a higher affinity towards DHFR compared with TopoIV. This behavior suggests that the energetic contributions associated with lipophilicity and electrostatic interactions play a determining role in the selectivity of these compounds toward the two therapeutic targets evaluated.

#### 2.3.3. Pharmacokinetic Properties

The pharmacokinetic properties of the synthesized active compounds provided valuable insights into their behavior in the human body. The series (**5a**–**e**) was evaluated for drug-likeness and ADMET characteristics using the SwissADME web tool (https://www.swissadme.ch) [27]. As shown in Table 6, all derivatives exhibit favourable drug-like properties and comply with Lipinski’s rule of five. The predicted ADMET profile indicates good human intestinal absorption and the potential to cross the blood–brain barrier. Notably, compound **5d** is not expected to be a substrate of P-glycoprotein, a feature that may contribute positively to its oral bioavailability. It is important to highlight that, although compounds (**5a**–**e**) exhibit favourable predicted ADME properties, these parameters mainly reflect human pharmacokinetic behavior and do not account for bacterial uptake. The lack of activity against Gram-negative bacteria may be attributed to their specific physiological features, including the presence of an outer membrane and reduced permeability, which are not captured by in silico ADME predictions.

## 3. Materials and Methods

### 3.1. Chemicals

All reagents were purchased from commercial suppliers, including Sigma-Aldrich, Merck, Ambeed, and AK Scientific, and used without further purification. Reactions conducted at room temperature are denoted as “rt” (approximately 25 °C). Magnetic stirring was performed using Teflon-coated stir bars, and reaction temperatures were controlled via Thermowatch-regulated silicone oil baths. Reaction progress was monitored by thin-layer chromatography (TLC) on Merck silica gel 60 F_254_ plates. Spots were visualized under UV light (254 or 365 nm) and/or by staining with *p*-anisaldehyde, oleum, phosphomolybdic acid, or cerium nitrate solutions followed by heating. Flash column chromatography was performed using silica gel (particle size 63–200 µm), unless otherwise specified. Organic phases were dried over anhydrous Na_2_SO_4_, and solvent removal (“concentration”) was carried out via rotary evaporation using a Büchi R-300 system, followed by high vacuum to eliminate residual solvents. Melting points were measured using a Stuart SMP3 apparatus (Staffordshire, UK). Infrared spectra were recorded on a Perkin-Elmer FT-IR Spectrometer Spectrum Two (Llantrisant, UK) using KBr pellets. NMR spectra (^1^H and ^13^C) were acquired in CDCl_3_ and DMSO-*d_6_* at 400 or 500 MHz on a Bruker Avance III spectrometer (Oxford, UK) or a 400 MHz Bruker Ascend TM (Bruker BioSpin, Billerica, MA, USA). Chemical shifts (δ) are reported in parts per million (ppm), referenced to residual solvent signals (CDCl_3_: δH = 7.26, δC = 77.16 ppm; DMSO-*d_6_*: δH = 2.50, 3.33, δ = 39.10), and coupling constants (*J*) are given in hertz (Hz). Mass spectra (ESI-MS) were obtained on an Agilent 1200 series system coupled to an Agilent QTof 6520 mass spectrometer with ESI/APCI ionization (Agilent Technologies, Santa Clara, CA, USA).

### 3.2. Synthetic Procedure

#### 3.2.1. General Procedure for the Synthesis of 2-arylquinazolin-4(3*H*)-one (**3a–e**)

A solution of anthranilamide (36.7 mmol) and benzaldehyde (36.7 mmol) in ethanol (37 mL) was stirred vigorously at 30 °C for 1 h until a white solid was formed. Then, 370 mL of an aqueous solution of I_2_/KI (0.1 M) was added, and the resulting mixture was stirred overnight at room temperature. To the resulting dark heterogeneous mixture, an aqueous solution of K_2_S_2_O_3_ (0.1 M) was added until discoloration (reduction in the remaining I_2_) was observed. The suspended solid was then filtered and crystallized from hot ethanol, obtaining the 2-arylquinazolin-4(3*H*)-ones as white, cloudy crystals.

2-phenylquinazolin-4(*3H*)-one (**3a**)

White solid, 70% yield. **m.p**.: 232–234 °C; **^1^H-NMR** (500 MHz, DMSO-*d*_6_) δ 8.17 (d, *J* = 7.1 Hz, 2H), 8.16 (dd, *J* = 7.9 Hz, 1H), 7.82 (t, *J* = 7.6 Hz, 1H), 7.73 (d, *J* = 8.1 Hz, 1H), 7.57 (t, *J* = 7.0 Hz, 1H), 7.53 (t, *J* = 7.5 Hz, H-14; 2H), 7.50 (t, *J* = 7.0 Hz, 1H). **^13^C-NMR** (126 MHz, DMSO) δ 121.0, 125.9, 126.6, 127.3, 127.8, 128.6, 131.4, 132.7, 134.6, 148.6, 152.4, 162.3. **FTIR** (KBr) [cm^−1^]: 3195 (N-H), 3061, 3036, and 3136 (CAr-H); 1668 (C=O); 1602 (C=N); 1557 (CAr-CAr). **HRMS** (ESI-TOF): *m*/*z* calculated for C_14_H_11_N_2_O [M + H]^+^: *m*/*z* 223,0862 found 223,0869.

2-(4-fluorophenyl)quinazolin-4(3*H*)-one (**3b**)

White solid, 83%. **m.p**.: 294–296 °C. **^1^H-NMR** (400 MHz, DMSO-*d*_6_) δ 12.58 (s, 1H), 8.35–8.21 (m, 2H), 8.15 (dd, *J* = 8.2, 1.2 Hz, 1H), 7.84 (ddd, *J* = 8.5, 7.1, 1.6 Hz, 1H), 7.74 (ddd, *J* = 8.2, 1.3, 0.6 Hz, 1H), 7.53 (ddd, *J* = 8.1, 7.1, 1.2 Hz, 1H), 7.45–7.33 (m, 2H). **^13^C-NMR** (126 MHz, DMSO) δ 164.0 (d, *J* = 249.5 Hz), 162.3, 151.4, 148.6, 134.6, 130.4 (d, *J* = 9.0 Hz), 129.3, 129.2, 127.4, 126.6, 125.8, 120.9, 115.6 (d, *J* = 22.0 Hz). **FTIR** (KBr) [cm^−1^]: 3179 (N-H); 1672 (C=O); 1610 (C=C Ar); 1289 (C=N); 1236 (C-F Ar). **HRMS** (ESI-TOF): *m*/*z* calculated for C_14_H_10_FN_2_O [M + H]^+^: *m*/*z* 241.0777, found 241.0779.

2-(4-chlorophenyl)quinazolin-4(3*H*)-one (**3c**)

White solid, 71%. **m.p**.: 329–331 °C. **^1^H-NMR** (500 MHz, DMSO-*d*_6_) δ 12.58 (s, 1H), 8.19 (d, *J* = 8.2 Hz, 2H), 8.14 (d, *J* = 7.9 Hz, 1H), 7.83 (t, *J* = 7.8 Hz, 1H), 7.73 (d, *J* = 8.2 Hz, 1H), 7.61 (d, *J* = 8.2 Hz, 2H), 7.52 (t, *J* = 7.6 Hz, 1H). **^13^C-NMR** (126 MHz, DMSO) δ 162.1, 151.3, 148.6, 136.3, 134.7, 131.5, 129.6, 128.7, 127.5, 126.8, 125.9, 121.0. **FTIR** (KBr) [cm^−1^]: 3179 (N-H); 1678 (C=O); 1603 (C=C Ar); 1346 (C-Cl); 1290 (C=N). **HRMS** (ESI-TOF): *m*/*z* calculated for C_14_H_10_ClN_2_O [M + H]^+^: *m*/*z* 257.0482, found 257.0483.

2-(4-bromophenyl)quinazolin-4(3*H*)-one (**3d**)

White solid, 79%. **m.p**.: 330–332 °C. **^1^H-NMR** (500 MHz, DMSO-*d*_6_) δ 12.59 (s, 1H), 8.17–8.08 (m, 3H), 7.84 (t, *J* = 7.7 Hz, 1H), 7.78–7.72 (t, *J* = 9.1 Hz, 3H), 7.53 (t, *J* = 7.6 Hz, 1H). **^13^C-NMR** (126 MHz, DMSO) δ 162.1, 151.4, 148.6, 134.7, 131.9, 131.6, 129.8, 127.5, 126.8, 125.9, 125.2, 121.0. **FTIR** (KBr) [cm^−1^]: 3181 (N-H); 1677 (C=O); 1602 (C=C Ar); 1348 (C-Br); 1311 (C=N). **HRMS** (ESI-TOF): *m*/*z* calculated for C_14_H_10_BrN_2_O [M + H]^+^: *m*/*z* 300.9977, found 300.9981.

2-(4-methoxyphenyl)quinazolin-4(3*H*)-one (**3e**)

White solid, 83%. **m.p**.: 247–249 °C. **^1^H-NMR** (500 MHz, DMSO-*d*_6_) δ 12.37 (s, 1H), 8.21–8.13 (m, 2H), 8.10 (dd, *J* = 7.8, 1.7 Hz, 1H), 7.78 (ddd, *J* = 8.4, 5.0, 1.5 Hz, 1H), 7.67 (d, *J* = 8.1 Hz, 1H), 7.45 (t, *J* = 7.5 Hz, 1H), 7.11–7.00 (m, 2H), 3.82 (d, *J* = 1.3 Hz, 3H). **^13^C-NMR** (126 MHz, DMSO) δ 162.3, 161.9, 151.8, 148.9, 134.5, 129.4, 127.3, 126.1, 125.8, 124.8, 120.7, 114.0, 55.4. **FTIR** (KBr) [cm^−1^]: 3181 (N-H); 1677 (C=O); 1605 (C=C Ar); 1253 (C-O). **HRMS** (ESI-TOF): *m*/*z* calculated for C_15_H_13_N_2_O_2_ [M + H]^+^: *m*/*z* 253.0977, found 253.0978.

#### 3.2.2. General Procedure for the Synthesis of 2-aryl-4-chloroquinazolines (**4a–e**)

A solution of 2-arylquinazolin-4(3*H*)-one **3** (18.0 mmol), 3.3 mL of POCl_3_ (36.0 mmol), 5.7 mL of *N*,*N*-diethylaniline (36.0 mmol) in dry toluene (60 mL) was heated at reflux for 6 h. The mixture was then cooled to 0 °C, and an aqueous solution of NH_4_Cl_(sat)_ was added carefully. The organic phase was separated, and the resulting aqueous phase was extracted with CH_2_Cl_2_ (3 × 20 mL). The mixed organic phases were dried over Na_2_SO_4_, filtered, evaporated, and the resulting crude was purified by flash chromatography (5% EtOAc:hexane) to afford the 4-chloro-2-arylquinazolines as white solids.

4-chloro-2-phenylquinazoline (**4a**)

White solid, 80%. **m.p**.: 129-131 °C. **^1^H-NMR** (500 MHz, CDCl_3_) δ 8.62–8.55 (m, 2H), 8.25 (dd, *J* = 8.4, 1.4 Hz, 1H), 8.09 (d, *J* = 8.5 Hz, 1H), 7.93 (ddd, *J* = 8.4, 6.9, 1.4 Hz, 1H), 7.66 (ddd, *J* = 8.2, 6.9, 1.2 Hz, 1H), 7.56–7.50 (m, 3H). **^13^C-NMR** (126 MHz, CDCl_3_) δ 162.7, 160.2, 152.0, 136.8, 135.0, 131.3, 129.1, 128.9, 128.8, 128.4, 126.0, 122.6. **FTIR** (KBr) [cm^−1^]: 3053 (C-H); 1554 (C=C); 1333 (C=N); 768 (C-Cl). **HRMS** (ESI-TOF): *m*/*z* calculated for C_14_H_10_FN_2_O [M + H]^+^: *m*/*z* 240.0454, found 240.0472.

4-chloro-2-(4-fluorophenyl)quinazoline (**4b**)

White solid, 86%. **m.p**.: 135-137 °C. **^1^H-NMR** (400 MHz, CDCl_3_) δ 8.66–8.54 (m, 2H), 8.25 (ddd, *J* = 8.4, 1.5, 0.7 Hz, 1H), 8.07 (dt, *J* = 8.4, 1.0 Hz, 1H), 7.94 (ddd, *J* = 8.4, 6.9, 1.4 Hz, 1H), 7.67 (ddd, *J* = 8.2, 6.9, 1.2 Hz, 1H), 7.24–7.14 (m, 2H). **^13^C-NMR** (126 MHz, CDCl_3_) δ (126 MHz, CDCl_3_) δ 165.1 (d, *J* = 251.5 Hz) 162.7, 159.3, 152.0, 135.1, 133.0, 133.0, 131.0 (d, *J* = 8.88 Hz), 129.0, 128.4, 126.0, 122.5, 115.8 (d, *J* = 21.7 Hz). **^19^F-NMR** (376 MHz, CDCl_3_) δ -109.48. **FTIR** (KBr) [cm^−1^]: 3057 (C-H); 1561 (C=C); 1330 (C=N); 1230 (C-F Ar); 759 (C-Cl). **HRMS** (ESI-TOF): *m*/*z* calculated for C_14_H_8_ClFN_2_ [M + H]^+^: *m*/*z* 258.0360, found 258.0385.

4-chloro-2-(4-chlorophenyl)quinazoline (**4c**)

White solid, 77%. **m.p**.: 167-169 °C. **^1^H-NMR** (500 MHz, CDCl_3_) δ 8.58–8.50 (m, 2H), 8.25 (dd, *J* = 8.4, 1.4 Hz, 1H), 8.08 (d, *J* = 8.7 Hz, 1H), 7.94 (ddd, *J* = 8.4, 7.0, 1.4 Hz, 1H), 7.68 (ddd, *J* = 8.2, 6.9, 1.2 Hz, 1H), 7.55–7.47 (m, 2H). **^13^C-NMR** (126 MHz, CDCl_3_) δ 162.8, 159.2, 152.0, 137.6, 135.3, 135.1, 130.2, 129.0, 128.6, 126.0, 122.7. **FTIR** (KBr) [cm^−1^]: 3055 (C-H); 1552 (C=C); 1329 (C=N); 1245 (C-Cl Ar); 759 (C-Cl). **HRMS** (ESI-TOF): *m*/*z* calculated for C_14_H_9_Cl_2_N_2_ [M + H]^+^: *m*/*z* 275.0143, found 275.0144.

4-chloro-2-(4-bromophenyl)quinazoline (**4d**)

White solid, 64%. **m.p**.: 155-157 °C. **^1^H-NMR** (500 MHz, CDCl_3_) δ 8.48–8.42 (m, 2H), 8.24 (dd, *J* = 8.4, 1.4 Hz, 1H), 8.07 (dt, *J* = 8.4, 0.8 Hz, 1H), 7.93 (ddd, *J* = 8.5, 7.0, 1.4 Hz, 1H), 7.69–7.61 (m, 3H) **^13^C-NMR** (126 MHz, CDCl_3_) δ 162.8, 159.3, 151.9, 135.7, 135.1, 132.0, 130.4, 129.0, 128.6, 126.2, 126.0, 122.7. **FTIR** (KBr) [cm^−1^]: 3049 (C-H); 1550 (C=C); 1324 (C=N); 1244 (C-Br Ar): 759 (C-Cl). **HRMS** (ESI-TOF): *m*/*z* calculated for C_14_H_9_BrClN_2_ [M + H]^+^: *m*/*z* 317.9559, found 317.9589.

4-chloro-2-(4-methoxyphenyl)quinazoline (**4e**)

White solid, 67%. **m.p**.: 119-121 °C. **^1^H-NMR** (500 MHz, CDCl_3_) δ 8.54 (d, *J* = 8.9 Hz, 2H), 8.19 (dd, *J* = 8.5, 1.4 Hz, 1H), 8.02 (d, *J* = 7.9 Hz, 1H), 7.88 (ddd, *J* = 8.4, 6.9, 1.4 Hz, 1H), 7.59 (ddd, *J* = 8.2, 6.9, 1.2 Hz, 1H), 7.02 (d, *J* = 8.9 Hz, 2H), 3.89 (s, 3H). **^13^C-NMR** (126 MHz, CDCl_3_) δ 162.4, 160.0, 152.1, 134.8, 130.6, 129.45, 128.8, 127.8, 125.9, 122.2, 114.1, 55.5 **FTIR** (KBr) [cm^−1^]: 3023 (C-H sp3); 1562 (C=C); 1335 (C=N); 1244 (C-O); 761 (C-Cl). **HRMS** (ESI-TOF): *m*/*z* calculated for C_15_H_12_ClN_2_O [M + H]^+^: *m*/*z* 271.0638, found 271.0640.

#### 3.2.3. General Procedure for the Synthesis of 2-aryl-4-aminoquinazolines (**5a–f**)

To a solution of 2-arylquinazoline (4.2 mmol) in DMF (10 mL) was added 1.2 mL of Et_3_N (4.8 mmol) and (3-aminopropyl)-imidazole (5.5 mmol) or butylamine (5.5 mmol) at room temperature. The mixture was stirred at 25 °C for 4 h, then 40 mL of cold water was poured into the solution. The resulting solid was filtered and crystallized over acetone to afford the *N*-(3-(1*H*-imidazol-1-yl)propyl)-2-arylquinazolin-4-amines (**5a**–**e**) and 2-(4-bromophenyl)-*N*-butylquinazolin-4-amine (**5f**) as yellowish crystals.

*N*-(3-(1*H*-imidazol-1-yl)propyl)-2-phenylquinazolin-4-amine (**5a**)

Yellowish crystals, 80%. **m.p**.: 177-179 °C. **^1^H-NMR** (500 MHz, CDCl_3_) δ 8.61–8.47 (m, 2H), 7.95–7.89 (m, 1H), 7.78 (dd, *J* = 8.2, 1.3 Hz, 1H), 7.72 (ddd, *J* = 8.4, 6.9, 1.3 Hz, 1H), 7.54–7.44 (m, 4H), 7.41 (t, *J* = 7.6 Hz, 1H), 7.10 (s, 1H), 6.98 (s, 1H), 6.46 (s, 1H) 4.11 (t, *J* = 6.7 Hz, 2H), 3.80 (q, *J* = 6.4 Hz, 2H), 2.31 (p, *J* = 6.7 Hz, 2H). **^13^C-NMR** (126 MHz, CDCl_3_) δ 160.5, 160.0, 150.7, 139.0, 137.3, 132.8, 130.3, 129.8, 129.0, 128.5, 128.4, 125.6, 121.0, 119.2, 113.9, 45.1, 38.6, 30.7. **FTIR** (KBr) [cm^−1^]: 3231 (N-H); 3058 (C-H sp2); 2958 (C-H sp3); 1574 (C=C); 1362 (C=N). **HRMS** (ESI-TOF): *m*/*z* calculated for C_20_H_20_N_5_ [M + H]^+^: *m*/*z* 330.1719, found 330.1723.

*N*-(3-(1*H*-imidazol-1-yl)propyl)-2-(4-fluorophenyl)quinazolin-4-amine (**5b**)

Yellowish crystals, 80%. **m.p**.: 229-231. **^1^H-NMR** (400 MHz, CDCl_3_) δ 8.55–8.48 (m, 2H), 7.91 (ddd, *J* = 8.4, 1.3, 0.6 Hz, 1H), 7.75 (ddd, *J* = 8.4, 6.9, 1.4 Hz, 1H), 7.62 (dd, *J* = 8.2, 1.1 Hz, 1H), 7.56 (s, 1H), 7.44 (ddd, *J* = 8.2, 7.0, 1.2 Hz, 1H), 7.21–7.13 (m, 2H), 7.12 (t, *J* = 1.1 Hz, 1H), 6.99 (t, *J* = 1.3 Hz, 1H), 5.68 (d, *J* = 6.0 Hz, 1H), 4.15 (t, *J* = 6.7 Hz, 2H), 3.85 (q, *J* = 6.7 Hz, 2H), 2.33 (p, *J* = 6.7 Hz, 2H). **^13^C-NMR** (126 MHz, CDCl_3_) δ 164.59 (d, *J* = 249.5 Hz), 159.6, 150.7, 137.3, 135.1, 132.9, 130.51 (d, *J* = 8.6 Hz), 130.0, 129.1, 125.8, 120.5, 119.1, 115.33 (d, *J* = 21.4 Hz), 113.6, 45.1, 38.8, 30.8, 29.8. **^19^F-NMR** (376 MHz, CDCl_3_) δ -111.35. **FTIR** (KBr) [cm^−1^]: 3229 (N-H); 3058 (C-H sp2); 2958 (C-H sp3); 1581 (C=C); 1362 (C=N); 1217(C-F Ar). **HRMS** (ESI-TOF): *m*/*z* calculated for C_20_H_19_N_5_ [M + H]^+^: *m*/*z* 348.1624, found 348.1629.

*N*-(3-(1*H*-imidazol-1-yl)propyl)-2-(4-chlorophenyl)quinazolin-4-amine (**5c**)

Yellowish crystals, 94%. **m.p**.: 219-221. **^1^H-NMR** (400 MHz, DMSO-*d*_6_) δ 8.45–8.37 (m, 3H), 8.24 (d, *J* = 8.2 Hz, 1H), 7.82–7.72 (m, 2H), 7.70 (s, 1H), 7.58–7.47 (m, 3H), 7.25 (s, 1H), 6.95 (s, 1H), 4.13 (t, *J* = 6.7 Hz, 2H), 3.65 (q, *J* = 6.6 Hz, 2H), 2.16 (p, *J* = 6.9 Hz, 2H). **^13^C-NMR** (101 MHz, DMSO-*d*_6_) δ 159.7, 158.2, 149.8, 137.4, 134.9, 132.8, 129.7, 129.6, 128.4, 128.3, 127.8, 125.0, 122.8, 119.5, 113.9, 44.0, 38.0, 30.2. **FTIR** (KBr) [cm^−1^]: 3227 (N-H); 3065 (C-H sp2); 2964 (C-H sp3); 1580 (C=C);1354 (C=N); 1231(C-Cl Ar). **HRMS** (ESI-TOF): *m*/*z* calculated for C_20_H_19_ClN_5_ [M + H]^+^: *m*/*z* 364.1329, found 364.1333.

*N*-(3-(1*H*-imidazol-1-yl)propyl)-2-(4-bromophenyl)quinazolin-4-amine (**5d**)

Yellowish crystals, 92%. **m.p**.: 203-205 °C. **^1^H-NMR** (500 MHz, CDCl_3_) δ 8.35–8.29 (m, 2H), 7.84 (d, *J* = 8.3 Hz, 1H), 7.67 (ddd, *J* = 8.4, 7.0, 1.3 Hz, 1H), 7.62 (d, *J* = 8.2 Hz, 1H), 7.57–7.52 (m, 3H), 7.37 (ddd, *J* = 8.3, 6.9, 1.2 Hz, 1H), 7.05 (s, 1H), 6.92 (s, 1H), 5.91 (s, 1H), 4.08 (t, *J* = 6.7 Hz, 2H), 3.76 (q, *J* = 6.5 Hz, 2H), 2.26 (p, *J* = 6.7 Hz, 2H). ^**13**^**C-NMR** (126 MHz, CDCl_3_) δ 159.9, 159.5, 150.6, 137.9, 133.0, 131.6, 130.1, 129.9, 129.1, 126.0, 125.0, 120.7, 119.1, 113.8, 45.1, 38.8, 30.7. **FTIR** (KBr) [cm^−1^]: 3227 (N-H); 3060 (C-H sp2); 2932 (C-H sp3); 1579 (C=C); 1352 (C=N); 1227(C-Br Ar). **HRMS** (ESI-TOF): *m*/*z* calculated for C_20_H_19_BrN_5_ [M + H]^+^: *m*/*z* 408.0824, found 408.0830.

*N*-(3-(1*H*-imidazol-1-yl)propyl)-2-(4-methoxyphenyl)quinazolin-4-amine (**5e**)

Yellowish crystals, 86%. **m.p**.: 145-147 °C. ^**1**^**H-NMR** (500 MHz, CDCl_3_) δ 8.53–8.37 (m, 2H), 7.88 (dd, *J* = 8.5, 1.2 Hz, 1H), 7.75 (dd, *J* = 8.3, 1.3 Hz, 1H), 7.69 (ddd, *J* = 8.3, 7.0, 1.3 Hz, 1H), 7.48 (d, *J* = 1.2 Hz, 1H), 7.36 (ddd, *J* = 8.1, 6.9, 1.2 Hz, 1H), 7.10 (d, *J* = 1.1 Hz, 1H), 7.03–6.98 (m, 2H), 6.97 (s, 1H), 6.41 (t, *J* = 5.8 Hz, 1H), 4.10 (t, *J* = 6.7 Hz, 2H), 3.88 (s, 3H), 3.77 (q, *J* = 6.5 Hz, 2H), 2.29 (p, *J* = 6.7 Hz, 2H). **^13^C-NMR** (126 MHz, CDCl_3_) δ 161.6, 160.3, 159.8, 150.8, 137.2, 132.7, 131.7, 130.0, 129.8, 128.8, 125.2, 121.0, 119.1, 113.8, 113.7, 55.5, 45.0, 38.6, 30.7. **FTIR** (KBr) [cm^−1^]: 3247 (N-H); 3057 (C-H sp2);2935 (C-H sp3); 1582 (C=C); 1354 (C=N); 1243 (C-O Ar). **HRMS** (ESI-TOF): *m*/*z* calculated for C_21_H_22_N_5_O [M + H]^+^: *m*/*z* 360.1824, found 360.1830.

2-(4-bromophenyl)-*N*-butylquinazolin-4-amine (**5f**)

Yellowish crystals, 83%. **m.p**.: 119-121 °C. **^1^H-NMR** (500 MHz, CDCl_3_) δ 8.45 (d, *J* = 8.5 Hz, 2H), 7.89 (d, *J* = 8.3 Hz, 1H), 7.72 (ddd, *J* = 8.2, 6.9, 1.4 Hz, 1H), 7.67 (d, *J* = 8.2 Hz, 1H), 7.61 (d, *J* = 8.5 Hz, 2H), 7.42 (ddd, *J* = 8.2, 7.0, 1.2 Hz, 1H), 5.70 (t, *J* = 5.5 Hz, 1H), 3.79 (td, *J* = 7.2, 5.5 Hz, 2H), 1.77 (p, *J* = 7.3 Hz, 2H), 1.52 (h, *J* = 7.4 Hz, 2H), 1.02 (t, *J* = 7.4 Hz, 3H). ^**13**^**C-NMR** (126 MHz, CDCl_3_) δ 159.8, 159.8, 150.5, 138.2, 132.7, 131.5, 130.2, 129.1, 125.7, 124.8, 120.5, 113.9, 41.2, 31.7, 20.4, 14.1. **FTIR** (KBr) [cm^−1^]: 3341 (N-H); 3057 (C-H sp2); 2937 (C-H sp3); 1574 (C=C); 1357 (C=N); 1219(C-Br Ar). **HRMS** (ESI-TOF): *m*/*z* calculated for C_18_H_19_BrN_3_ [M + H]^+^: *m*/*z* 356.0762, found 356.0768.

### 3.3. Antibacterial Assays

#### 3.3.1. Bacterial Culture Preparation

The bacterial strains were inoculated from frozen bacterial stocks of reference strains from French (CIP- and CRBI-) or American (ATCC-) national collections or clinical strains (E-, N- and n-HUS (Hopitaux Universitaires de Strasbourg- collection)). All strains were grown overnight in MH broth without antibiotics at 37 °C under shaking at 220 rpm. The OD at 600 (OD600) was adjusted to 0.2, and cells were re-suspended in fresh MH medium. The cultures were then incubated until they reached an OD600 of 1.0. In all experiments, bacteria were diluted when they were in the logarithmic phase of growth. The cells were then pelleted by centrifugation at 12,000 rpm for 3 min to remove the media and resuspended in fresh MH medium before each assay.

#### 3.3.2. Agar-Diffusion Assay

Twenty microliters of MH agar were distributed into sterile Petri dishes. The agar was left to set, and each plate was inoculated with a suspension of *S. aureus* ATCC 25923 or *P. aeruginosa* ATCC 27853. Wells of 5 mm in diameter were cut using a sterile cork borer, and the agar discs were removed. Alternate wells were filled with 20 µL of compound solution at concentrations of 50, 100, and 200 µg/mL and allowed to diffuse at room temperature for two hours. The plates were then incubated in the upright position at 37 °C for 18 h. DMSO and ciprofloxacin were used as negative and positive controls, respectively. The diameters of the growth inhibition zones were measured, and mean values were calculated.

#### 3.3.3. Agar-Dilution Assay

Each bacterial culture was diluted in sterile distilled water to obtain approximately 1 × 10^7^ CFU/mL, and 1 µL of each (approximately 1 × 10^4^ CFU/spot) was applied to the test medium using a Steers inoculator. Then, 250 µL of the compound solution in DMSO was added to 1.75 mL of sterile distilled water in a Petri dish, and MH agar was added to a final volume of 20 mL. Afterward, the mixture was homogenized. After agar solidification, each culture strain solution in sterile distilled water was applied to the test medium using a Steers inoculator. Petri dishes were then incubated for 18–24 h at 37 °C in aerobic conditions. The respective DMSO blank was conducted under the same conditions and was determined as non-toxic (1.25% final concentration). MICs from pure compounds were determined by evaluation at 8 different concentrations (100, 50, 20, 10, 5, 2, and 1 µg/mL). The MICs values were recorded as the lowest concentration at which no bacterial growth was observed. The assay was run in triplicate and repeated three times.

#### 3.3.4. Broth-Dilution Assay

Serial dilutions were prepared for compounds ranging from 200 to 10 µg/mL in LB broth (final concentration of 2.5% DMSO). Then, 100 µL of each dilution was placed in each well of a 96-well plate. Then, selected bacterial cultures were prepared in LB broth to obtain a suspension of 5 × 10^5^ CFU/mL, and 100 µL were added to each well. The respective negative (DMSO blank) and positive controls (ofloxacin and erythromycin) were also prepared. Plates were incubated overnight at 37 °C by shaking at 200 rpm. MICs were evaluated by the absence of bacterial growth (absorbance measured at OD600). The assay was run in triplicate and repeated three times.

### 3.4. Cytotoxic Assay

The cytotoxicity of the compounds was assessed using the HepG2 hepatocarcinoma cell line. Briefly, cells (5000 cells/well) were seeded in 96-well plates for 24 h at 37 °C with 5% CO_2_. Compounds were solubilized in DMSO to obtain stock solutions at a concentration of 20 mg/mL. These solutions were diluted in culture medium (DMEM: Dulbecco’s Modified Eagle’s Medium) to obtain various concentrations (0.05 to 100 μg/mL) for treating cells. Plates were incubated (37 °C with 5% CO_2_) for 72 h, then the medium was replaced by MTT solution, prepared by dissolving 15 mg of MTT salt (3-(4,5-dimethylthiazol-2-yl)-2,5-diphenyl-tetrazoliumbromide) in 50 mL (5 mL PBS plus 45 mL DMEM). After 45 min of incubation, the MTT solution was replaced with 100 µL of DMSO in the wells, and absorbance was measured spectrophotometrically at 570 and 620 nm to quantify the formazan formed by MTT reduction. The assay was run in triplicate and repeated three times.

### 3.5. Statistical Analysis

All antimicrobial and cytotoxic assays were performed in triplicate within the same experiment, and each experiment was independently repeated three times. MIC values are reported as the mean of the independent experiments. Standard deviations were calculated from these independent measurements.

### 3.6. Computational Methods

#### 3.6.1. Preparation of Ligands and DHFR Protein

Three-dimensional modeling of the synthesized compounds was initiated by converting their SMILES representations into three-dimensional structures using Omega software (version 6.1.1.1), a component of the OpenEye Suite package (version 2025.2). Subsequently, partial charges were assigned using the AM1-Bcc force field [47] implemented in the QUACPAC module, version 2.2.5.1. Finally, energy minimization was performed to optimize the dihedral angles of the molecules.

To prepare the receptor, the crystallographic structure of dihydrofolate reductase (PDB ID: 2W9H) was downloaded from the Protein Data Bank [48]. This structure has a resolution of 1.48 Å. The selected protein has trimethoprim as a co-crystallized ligand, which will be helpful as a reference molecule for delimiting the binding site of the synthesized compounds and for comparative evaluation of their affinity energies. The protein was prepared by the addition of hydrogen atoms and assignment of partial charges using the OPLS4 force field [49]. In addition, the protonation states of the polar amino acid residues were adjusted according to their pKa values, which were determined using the PropKa program (version 3.1). Subsequently, the side chains were minimized to reduce the steric overlap and refine the corresponding dihedral angles.

#### 3.6.2. Preparation of *Staphylococcus aureus* Topoisomerase IV

Considering the characteristic planarity of the synthesized quinazolines, it was proposed that the synthesized compounds could exert their action on *Staphylococcus aureus* topoisomerase IV in a manner analogous to fluoroquinolones. To this end, topoisomerase IV was modeled using the AlphaFold 2.0 [50]. Subunit A of S. aureus topoisomerase IV (corresponding to PDB ID: 2INR [51]) was used as the input sequence, and the crystallographic structure with PDB ID: 8C41 [52] was used as the template. This protein includes the bacterial DNA sequence and the co-crystallized ligand delafloxacin, whose spatial arrangement was taken into consideration. Additionally, magnesium atoms involved in coordination with delafloxacin and DNA were incorporated into the model to simulate the potential anchoring mode of the synthesized compounds.

The resulting model of *S. aureus* topoisomerase IV underwent preprocessing similar to that described for DHFR, which included the addition of hydrogen atoms, assignment of partial charges, adjustment of the protonation states of acidic and basic amino acid residues, and minimization of side chains. To define the grid, the center of mass of the delafloxacin ligand present in the generated model was used, and its dimensions were adjusted to accommodate the imidazole substructure of the compounds of interest adequately.

Molecular docking studies for both proteins (DHFR and topoisomerase IV) were performed using Glide software (version 4.6) [53], applying Standard Precision (SP) and eXtra precision (XP) modes. Using the SP mode, five of the best docking poses were generated for each ligand. Subsequently, the pose with the best score obtained in the SP was refined using XP mode for a more rigorous evaluation of affinity. Next, the optimal pose obtained through molecular docking was used to calculate the free binding energy using the Born Generalized Surface Area Molecular Mechanics (MM-GBSA) method [54] using Prime software, version 6.1. This calculation involved minimizing protein residues located within a 6 Å sphere centered on the center of mass of each bound ligand. The energy values are expressed in kcal/mol and listed in Table 5. Visual representations of the interactions (Figure 1 and Figure 2) were generated using PyMOL software (version 3.0.3).

## 4. Conclusions

This work demonstrates that the combination of a 2-aryl-4-aminoquinazoline scaffold with an aminoalkylimidazole linkage can generate compounds displaying selective antibacterial activity against *S. aureus*, with moderate cytotoxicity and favourable predicted pharmacokinetic properties. The efficient, scalable and high-yielding synthetic protocols enabled a systematic evaluation of structure–activity relationships, highlighting the influence of subtle structural modifications (such as the nature of the *para*-halogen substituent on the phenyl ring) on antibacterial activity.

Molecular docking studies provided valuable insights into how these structural variations may affect key interactions with potential bacterial targets. In particular, significant interactions were observed between dihydrofolate reductase (DHFR) or topoisomerase IV (TopoIV) and compounds **5c** and **5d**, which exhibited binding modes comparable to those of selected reference inhibitors. Importantly, although the imidazole moiety contributed additional interactions in the docking models, its removal did not switch off the antibacterial activity, suggesting flexibility for further pharmacomodulation of this series.

Overall, these results support the interest in this scaffold as an early-stage starting point for the development of species-selective anti-*S. aureus* agents. Such selectivity may offer advantages in limiting collateral effects on the microbiota and in reducing the selective pressure associated with the emergence of antibacterial resistance.

## Figures and Tables

**Table 1:** In vitro antibacterial activity of synthesized compounds by agar diffusion test (mm) at different concentrations (µg/mL) against model bacteria species.

Comp.	*Staphylococcus aureus* ATCC 25.923			*Pseudomonas aeruginosa* ATCC 27.853		
	Concentration			Concentration		
	50	100	200	50	100	200
**3a**	0	0	0	0	0	0
**3b**	0	0	0	0	0	0
**3c**	0	0	0	0	0	0
**3d**	0	0	0	0	0	0
**3e**	0	0	0	0	0	0
**4a**	0	0	0	0	0	0
**4b**	0	0	0	0	0	0
**4c**	0	0	0	0	0	0
**4d**	0	0	0	0	0	0
**4e**	0	0	0	0	0	0
**5a**	0	0	11.5 ± 0.5	0	0	21.5 ± 0.5
**5b**	8.0 ± 0.0	11.0 ± 1.0	15.5 ± 0.5	21.5 ± 0.5	25.5 ± 0.5	29.0 ± 0.0
**5c**	14.5 ± 0.5	18.5 ± 0.5	19.5 ± 0.5	25.0 ± 0.0	32.0 ± 0.0	32.5 ± 0.5
**5d**	17.0 ± 1.0	20.0 ± 0.0	22.0 ± 0.0	26.0 ± 0.0	30.0 ± 1.0	30.5 ± 0.5
**5e**	0	0	9.5 ± 0.5	0	0	18.5 ± 0.5
**Ciprofloxacin**	22.2 ± 0.2	24.0 ± 0.0	26.0 ± 0.0	30.5 ± 0.5	33.5 ± 0.5	35.0 ± 1.0

**Table 2:** In vitro antibacterial minimal inhibitory concentration of synthesized compounds by agar dilution test (µM).

Bacteria Strain		Code	Compounds and Positive Control					
			5a	5b	5c	5d	5e	Ofloxacin
**Gram-negative Strains**	*Achromobacter xylosoxidans denitrificans*	CIP 71.32	>303.6	>287.8	>274.8	>245.0	>278.2	88.6
	*Achromobacter xylosoxidans*	CIP 77.15	>303.6	>287.8	>274.8	>245.0	>278.2	5.5
	*Acinetobacter baumannii*	CIP 70.34	>303.6	>287.8	>274.8	>245.0	>278.2	2.8
	*Aeromonas hydrophila*	CIP 76.14	>303.6	>287.8	>274.8	>245.0	>278.2	≤0.2
	*Burkholderia cepacia*	CIP 80.24	>303.6	>287.8	>274.8	>245.0	>278.2	22.2
	*Citrobacter freundii*	CIP 57.32	>303.6	>287.8	>274.8	>245.0	>278.2	≤0.2
	*Citrobacter koseri*	CIP 82.87	>303.6	>287.8	>274.8	>245.0	>278.2	0.3
	*Comamonas testosteroni*	N70	>303.6	>287.8	>274.8	>245.0	>278.2	11.1
	*Enterobacter aerogenes*	CIP 6086T	>303.6	>287.8	>274.8	>245.0	>278.2	0.7
	*Enterobacter cloacae*	CIP 60.85	>303.6	>287.8	>274.8	>245.0	>278.2	≤0.2
	*Escherichia coli*	ATCC 25922	>303.6	>287.8	>274.8	>245.0	>278.2	≤0.2
	*Klebsiella oxytoca*	E282	>303.6	>287.8	>274.8	>245.0	>278.2	0.3
	*Klebsiella pneumoniae*	CIP 52.145	>303.6	>287.8	>274.8	>245.0	>278.2	0.3
	*Klebsiella pneumoniae*	ATCC10031	>303.6	>287.8	>274.8	>245.0	>278.2	≤0.2
	*Klebsiella pneumoniae*	ATCC 700603	>303.6	>287.8	>274.8	>245.0	>278.2	5.5
	*Providencia stuartii*	CIP 107.808	>303.6	>287.8	>274.8	>245.0	>278.2	88.6
	*Pseudomonas aeruginosa*	CIP 103.467	>303.6	>287.8	>274.8	>245.0	>278.2	5.5
	*Pseudomonas aeruginosa*	ATCC 27853	>303.6	>287.8	>274.8	>245.0	>278.2	11.1
	*Pseudomonas aeruginosa*	CIP A22	>303.6	>287.8	>274.8	>245.0	>278.2	5.5
	*Pseudomonas putida*	CIP 55.191	>303.6	>287.8	>274.8	>245.0	>278.2	2.8
	*Salmonella enterica*	CIP 58.58	>303.6	>287.8	>274.8	>245.0	>278.2	≤0.2
	*Serratia marcescens*	CIP 60.93	>303.6	>287.8	>274.8	>245.0	>278.2	1.4
	*Stenotrophomonas maltophilia*	CIP 54.90	>303.6	>287.8	>274.8	>245.0	>278.2	5.5
	*Vibrio parahaemolyticus*	CIP 75.2	>303.6	>287.8	>274.8	>245.0	>278.2	0.3
	*Yersinia enterocolitica*	CIP 80.27	>303.6	>287.8	>274.8	>245.0	>278.2	≤0.2
**Gram-positive Strains**	*Bacillus cereus*	CIP 6624	>303.6	>287.8	>274.8	>245.0	>278.2	1.4
	*Corynebacterium minutissimum*	CIP 100652T	>303.6	>287.8	>274.8	>245.0	>278.2	0.7
	*Enterococcus avium*	CIP 104 053	>303.6	>287.8	>274.8	>245.0	>278.2	11.1
	*Enterococcus casselliflavus*	CIP 103.018	>303.6	>287.8	>274.8	>245.0	>278.2	11.1
	*Enterococcus durans*	CIP 104 999	>303.6	>287.8	>274.8	>245.0	>278.2	5.5
	*Enterococcus faecalis*	CIP 103.214	>303.6	>287.8	>274.8	>245.0	>278.2	5.5
	*Enterococcus faecium*	CIP 103.014	>303.6	>287.8	>274.8	>245.0	>278.2	22.2
	*Enterococcus gallinarum*	CIP 105.985	>303.6	>287.8	>274.8	>245.0	>278.2	11.1
	*Enterococcus hirae*	CIP 5855	>303.6	>287.8	>274.8	>245.0	>278.2	5.5
	*Listeria monocytogenes*	CIP 103.575	>303.6	>287.8	>274.8	>245.0	>278.2	5.5
	*Staphylococcus aureus*	ATCC 25.923	60.7	278.8	13.7	12.2	27.8	1.4
	*Staphylococcus aureus*	CIP 53.156	151.8	>287.8	27.5	24.5	55.6	0.7
	*Staphylococcus aureus*	CIP 53.154	60.7	>287.8	27.5	24.5	27.8	0.7
	*Staphylococcus aureus*	ATCC 29213	151.8	278.8	13.7	24.5	27.8	1.4
	*Staphylococcus aureus*	E372	30.3	>287.8	27.5	24.5	27.8	11.1
	*Staphylococcus aureus*	E378	60.7	>287.8	13.7	24.5	55.6	11.1
	*Staphylococcus aureus*	N217	30.3	>287.8	13.7	12.2	27.8	1.4
	*Staphylococcus aureus*	N587	60.7	>287.8	27.5	24.5	55.6	1.4
	*Staphylococcus aureus*	N588	30.3	>287.8	13.7	12.2	27.8	1.4
	*Staphylococcus aureus*	N589	60.7	>287.8	27.5	24.5	27.8	177.1
	*Staphylococcus aureus*	N594	30.3	>287.8	27.5	24.5	27.8	1.4
	*Staphylococcus epidermidis*	CIP 53.124	151.8	>287.8	137.4	122.5	278.2	1.4
	*Staphylococcus haemolyticus*	CIP 81.56	303.6	>287.8	274.8	245	278.2	0.7
	*Staphylococcus lugdunensis*	ATCC 43.809	151.8	>287.8	274.8	245	278.2	2.8
	*Staphylococcus saprophyticus*	CIP 76125	151.8	>287.8	274.8	245	278.2	5.5

**Table 3:** In vitro antibacterial minimal inhibitory concentration of synthesized compounds by broth dilution test (µM).

Bacterial Strain	Compounds and Positive Control						
	5a	5b	5c	5d	5e	5f	Erythromycin
*Staphylococcus aureus* HG001	70.2	>575.6	4.4	2.2	70.2	7.3	0.4
*Staphylococcus aureus* Clinical Isolate 1 (1-HUS)	36.5	>575.6	2.9	2.9	36.5	8.8	0.3
*Staphylococcus aureus* Clinical Isolate 2 (2-HUS)	35.2	>575.6	2.2	2.2	35.2	8.8	0.5
*Escherichia coli* MG1655	>607.2	>575.6	>549.6	>490.0	>556.4	>562	85.2

**Table 4:** Antiproliferative activity and selectivity index of compounds (**5a**–**e**) against the HepG2 cell line in vitro. n.d.: not determined.

Compound	IC_50_ (µM)	Selectivity Index (IC_50_/MIC Against *S. aureus* HG001)
**5a**	30.9 ± 2.6	0.4
**5b**	≥287.8 ± 0.0	n.d.
**5c**	22.0 ± 1.6	5.0
**5d**	15.7 ± 1.4	7.1
**5e**	18.5 ± 5.0	0.2
**Camptothecin**	0.19 ± 0.02	n.d.

**Table 5:** Values expressed in kcal/mol, Standard Precision (SP), and eXtra Precision (XP) functional score.

Comp.	DHFR			TopoIV		
	SP	XP	MM-GBSA	SP	XP	MM-GBSA
**5c**	−8.08	−7.25	−61.65	−4.69	−5.48	−45.15
**5d**	−8.00	−7.01	−64.58	−4.60	−5.29	−34.92
**5b**	−7.16	−5.83	−52.59	−4.80	−5.03	−24.04
**Trimetropim**	−7.95	−7.34	−72.09	-	-	-
**Delafloxacin**	-	-	-	−9.68	−6.97	−56.55

**Table 6:** Prediction of drug-likeness characteristics and ADMET properties of 2-aryl-aminoquinazolines (**5a**–**e**).

Comp.	5a	5b	5c	5d	5e
**Molecular weight**	329.40	347.39	363.84	408.29	359.42
**Topological polar surface área (Å)**	55.63	55.63	55.63	55.63	64.88
**WLogP**	3.8	4.36	4.46	4.57	3.81
**HBA**	3	4	3	3	4
**HBD**	1	1	1	1	1
**Lipinski violations**	0	0	0	0	0
**Drug-likeness**	Yes	Yes	Yes	Yes	Yes
**Bioavailability Score**	0.55	0.55	0.55	0.55	0.55
**GI absorption**	High	High	High	High	High
**BBB permeant**	Yes	Yes	Yes	Yes	Yes
**Pgp-substrate**	Yes	Yes	No	Yes	Yes

## Data Availability

Data is contained within the article and the Supplementary Materials.

## Supplementary Materials

The following supporting information can be downloaded at: https://www.mdpi.com/article/10.3390/ijms27062529/s1.
