# Supplementary material for: Synthesis of 2-Aryl-4-aminoquinazolines: Design, Molecular Docking, and In Vitro Assessment of Antibacterial and Cytotoxic Potential
Source: Int J Mol Sci. 2026 Mar 10;27(6):2529. doi: 10.3390/ijms27062529 (PMC13026357; doi:10.3390/ijms27062529)
Supplement: Supplementary file 1 [file ijms-27-02529-s001.zip › ijms-4110402-supplementary.pdf]

Supporting Information

Synthesis of 2-Aryl-4-aminoquinazolines: Design, Molecular Docking, and In Vitro Assessment of Antibacterial and Cytotoxic Potential

Felipe Verdugo [1], Capucine Braillon [2,3], Sana Mahjoub [2,4], Alejandro Castro-Alvarez [5,6], Régine Janel-Bintz [2], Pierre Fechter [2], Pascal Villa [7], Claudio A. Jiménez [1], Diego A. Donoso-Ruiz [1], Marcia Pérez-Fehrmann [8], Víctor Kesternich [8], Sergio Ortiz [3,*] and Ronald Nelson [8,*]

1 Departamento de Química Orgánica, Facultad de Ciencias Químicas, Universidad de Concepción, Edmundo Larenas 129, Concepción 4070371, Chile; felipeverdugo@udec.cl, cjimenez@udec.cl, ddonoso2018@udec.cl
2 CNRS, UMR 7242, Biotechnologie et Signalisation Cellulaire, Institut de Recherche de l'Ecole de Biotechnologie de Strasbourg, Université de Strasbourg, 67400 Illkirch-Graffenstaden, France; capucine.braillon@etu.unistra.fr, sanamahjoub0903@gmail.com, regine.janel@unistra.fr, p.fechter@unistra.fr;
3 UMR 7200 Laboratoire d'Innovation Thérapeutique, CNRS, Strasbourg Drug Discovery and Development Institute (IMS), Université de Strasbourg, 67400 Illkirch-Graffenstaden, France; capucine.braillon@etu.unistra.fr, ortizaguirre@unistra.fr
4 Laboratory of Human Genome and Multifactorial Diseases (LR12ES07), Faculty of Pharmacy, University of Monastir, Tunisia; sanamahjoub0903@gmail.com
5 Departamento de Ciencias Preclínicas, Facultad de Medicina, Universidad de La Frontera, Temuco 4811230, Chile; alejandro.castro.a@ufrontera.cl
6 Millennium Nucleus Bioproducts, Genomics and Environmental Microbiology (BioGEM), Valparaíso 2390123, Chile; alejandro.castro.a@ufrontera.cl
7 PCBIS Plateforme de Chimie Biologie Intégrative de Strasbourg, UAR 3286 CNRS/Université de Strasbourg, F-67000 Strasbourg, France; pvilla@unistra.fr
8 Departamento de Química, Facultad de Ciencias, Universidad Católica del Norte, Avda. Angamos 0610, Antofagasta 1270709, Chile; maperez@ucn.cl, vkestern@ucn.cl, rnelson@ucn.cl
* Correspondence: ortizaguirre@unistra.fr (S.O.); rnelson@ucn.cl (R.N.)

Table of Contents

Molecular docking figures 2
General Procedures 4
SYNTHESIS OF QUINAZOLINE DERIVATIVES 5
Synthesis of quinazolin-4(3H)-ones (3a-e). 5
Synthesis of 4-chloro-quinazolines (4a-e). 7
Synthesis of N-(3-(1H-imidazol-1-yl)propyl)-quinazolin-4-amines (5a-e). 9
Selected Spectra 12

Molecular docking figures

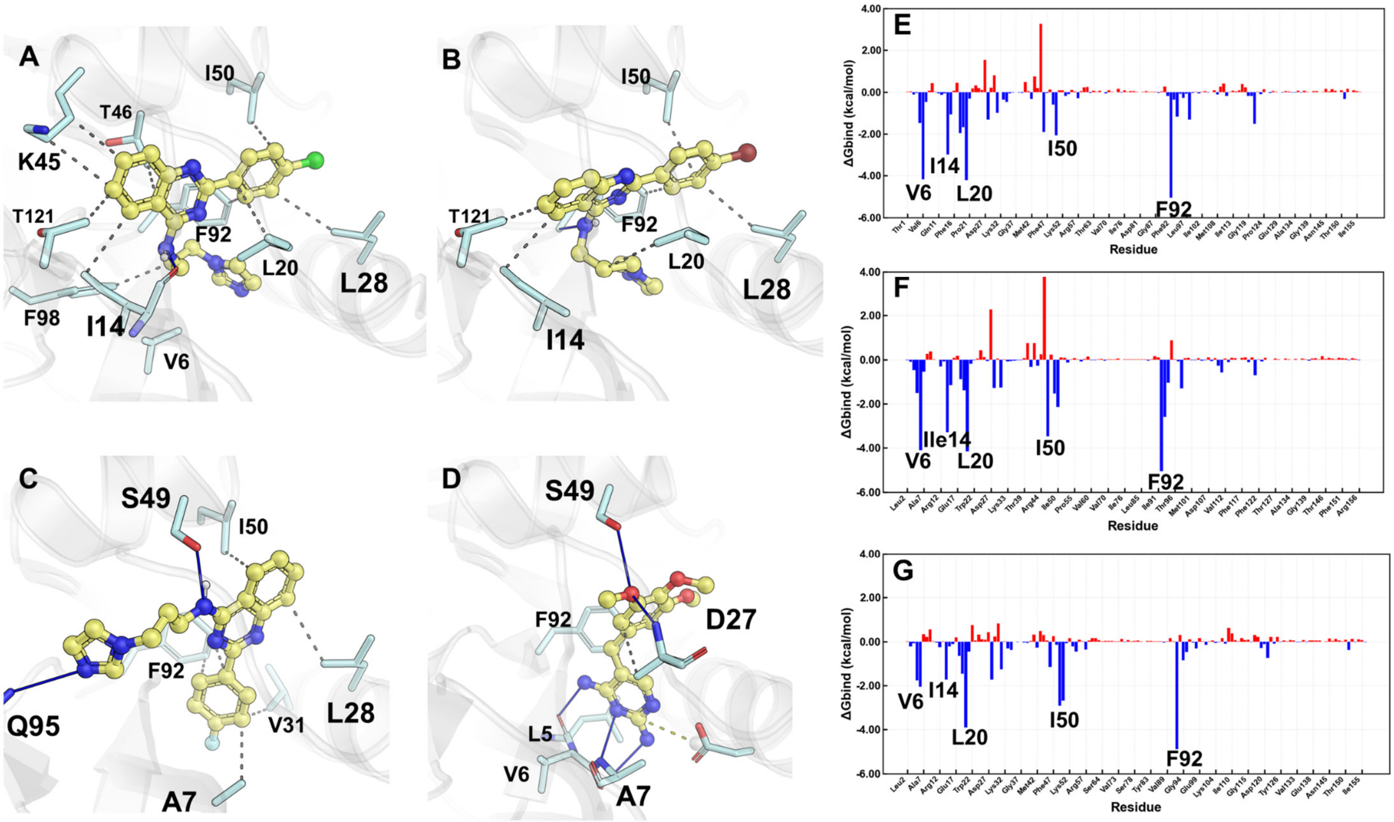

Figure S1. Figures and plots with larger size and resolution than Figure 2.

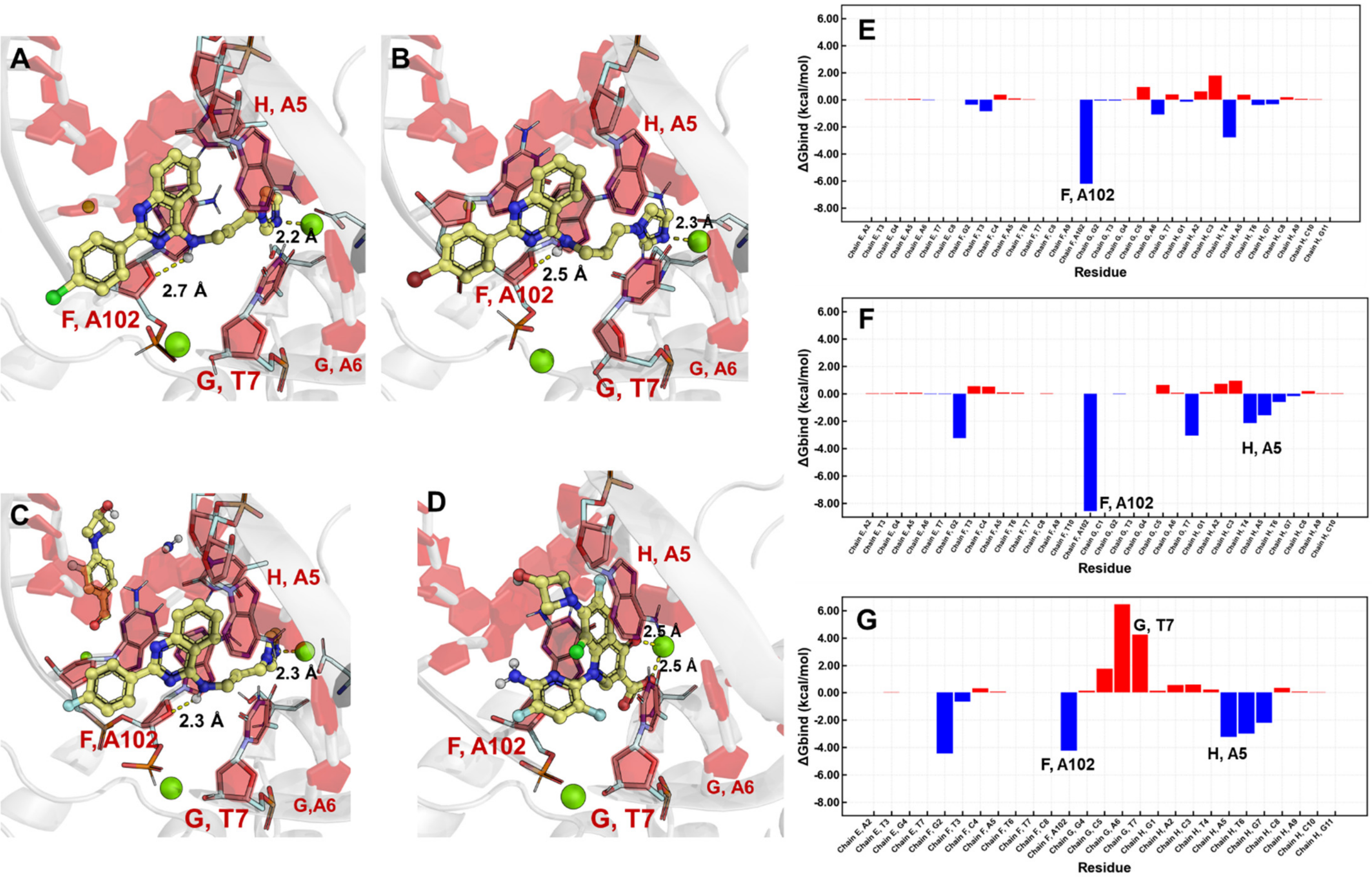

Figure S2. Figures and plots with larger size and resolution than Figure 3.

General Procedures

All reactions were conducted using HPLC grade solvents or similar under air. The abbreviation “rt” refers to reactions carried out at 20-25°C. Reaction mixtures were stirred using Teflon-coated magnetic stir bars. Reaction temperatures were maintained using Thermowatch-controlled silicone oil baths. All reagents for the synthesis of precursors were purchased from Sigma-Aldrich or Merck Millipore. Thin-layer chromatography (TLC) was performed on silica gel plates and components were visualized by observation under UV light, or by treating the plates with either *p*-anisaldehyde, ninhydrin, potassium permanganate or cerium nitrate solutions, followed by heating. Flash chromatography was carried out on silica gel unless otherwise stated. Dryings were performed with anhydrous Na_2SO_4. Concentration refers to the removal of volatile solvents via distillation using a Buchi rotary evaporator, followed by residual solvent removal under high vacuum.

NMR spectra (^{1}H and ^{13}C) were acquired in $CDCl_3$ and DMSO-*d*$_6$ at 400 or 500 MHz on a Bruker Avance III spectrometer or a 400 MHz Bruker Ascend TM (California, USA). Chemical shifts (δ) are reported in parts per million (ppm), referenced to residual solvent signals ($CDCl_3$: δH = 7.26, δC = 77.16 ppm; DMSO-*d*$_6$: δH = 2.50, 3.33, δ = 39.10), and coupling constants (*J*) are given in hertz (Hz). NMR spectra were analyzed using MestreNova© processing software (www.mestrelab.com). ^{1}H-NMR spectral data are reported as follows: chemical shift (δ ppm), multiplicity (s = singlet, d = doublet, t = triplet, q = quartet, dd = double doublet, td = triple doublet, m = multiplet, br = broad), integration. Data for ^{13}C are reported in terms of chemical shift relative to the residual solvent peak. Mass spectra (ESI-MS) were obtained on an Agilent 1200 series system coupled to an Agilent QTof 6520 mass spectrometer with ESI/APCI ionization. Melting points were measured using a Stuart SMP3 apparatus. Infrared spectra were recorded on a Perkin-Elmer FT-IR Spectrometer Spectrum Two using KBr pellets. Absorption bands were informed in cm^{-1}.

SYNTHESIS OF QUINAZOLINE DERIVATIVES

Synthesis of quinazolin-4(3H)-ones (3a-e).

Exemplified for the synthesis of 2-phenylquinazolin-4(3H)-one (**3a**)

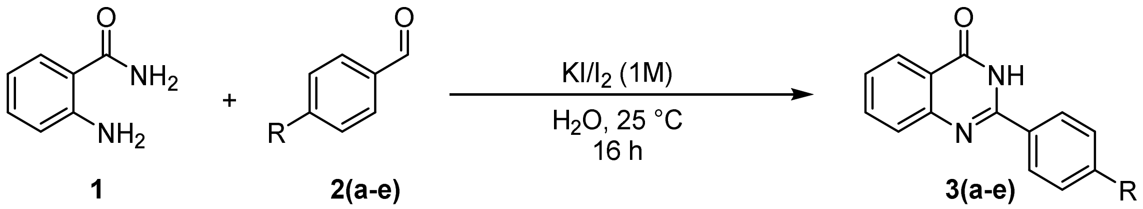

A solution of anthranilamide (5.00 g, 36.7 mmol, 1 eq.) and benzaldehyde (3.90 g, 36.7 mmol, 1 eq.) in ethanol (37 mL) was stirred vigorously at 30 ºC for 1h until the formation of a white solid. Then, 370 mL of an aqueous solution of I_2/KI (0.1 M) was added and the resulting mixture was stirred overnight. Over the resulting solution was added an aqueous solution of $K_2S_2O_3$ (0.1M) until the notorious discoloration of the mixture (reduction of remaining I_2). The solid was filtered and crystallized over hot ethanol, obtaining 4.90 g (22.4 mmol, 61%) of 2-phenylquinazolin-4(3H)-one (**1a**) as white crystals. The spectroscopic data were consistent with the reported in the literature.[1] **^{1}H-NMR** (500 MHz, DMSO-*d_6*) δ 8.17 (d, *J* = 7.1 Hz, 2H) , 8.16 (dd, *J* = 7.9 Hz, 1H), 7.82 (t, *J* = 7.6 Hz, 1H), 7.73 (d, *J* = 8.1 Hz, 1H), 7.57 (t, *J* = 7.0 Hz, 1H), 7.53 (t, *J* = 7.5 Hz, H-14; 2H), 7.50 (t, *J* = 7.0 Hz, 1H). **^{13}C-NMR** (126 MHz, DMSO) δ 121.0, 125.9, 126.6, 127.3, 127.8, 128.6, 131.4, 132.7, 134.6, 148.6, 152.4, 162.3. **FTIR** (KBr) [cm^{-1}]: 3195 (N-H), 3061, 3036, and 3136 (CAr-H); 1668 (C=O); 1602 (C=N); 1557 (CAr-CAr). **HRMS** (ESI-TOF): m/z 223,0862 calculated for $C_{14}H_{11}N_2O$ [M+H]$^+$, found 223,0869. **FTIR (KBr) [cm^{-1}]:** 3200 (N-H); 1670 (C=O); 1605 (C=C ar); 1295 (C=N ar). **mp:** 233 °C

[1] Ref [31]: Bakavoli, M.; Shiri, A.; Ebrahimpour, Z.; Rahimizadeh, M. Clean Heterocyclic Synthesis in Water: I2/KI Catalyzed One-Pot Synthesis of Quinazolin-4(3H)-Ones. *Chinese Chemical Letters* **2008**, *19*, 1403–1406, doi:10.1016/j.cclet.2008.07.016.

2-(4-fluorophenyl)quinazolin-4(3H)-one (3b)

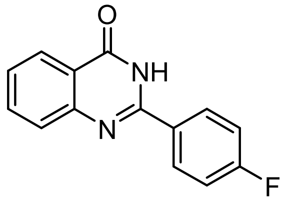

Obtained 4.63 g of **1b** (83%) as white crystals.**^{1}H-NMR** (400 MHz, DMSO-*d*$_6$) δ 12.58 (s, 1H), 8.35 – 8.21 (m, 2H), 8.15 (dd, *J* = 8.2, 1.2 Hz, 1H), 7.84 (ddd, *J* = 8.5, 7.1, 1.6 Hz, 1H), 7.74 (ddd, *J* = 8.2, 1.3, 0.6 Hz, 1H), 7.53 (ddd, *J* = 8.1, 7.1, 1.2 Hz, 1H), 7.45 – 7.33 (m, 2H). **^{13}C-NMR** (126 MHz, DMSO) δ 164.03 (d, *J* = 249.6 Hz), 162.3, 151.4, 148.6, 134.6, 130.35 (d, *J* = 8.9 Hz), 129.3, 129.2, 127.4, 126.6, 125.8, 120.9, 115.60 (d, *J* = 22.0 Hz). **^{19}F-NMR** (376 MHz, DMSO-*d*$_6$) δ -109.07 (td, *J* = 9.0, 4.6 Hz). **HRMS** (ESI-TOF): m/z calculated for $C_{14}H_{10}FN_2O$ [M+H]$^+$: m/z 241.0777, found 241.0779. **FTIR (KBr) [cm^{-1}]:** 3179 (N-H); 1672 (C=O); 1610 (C=C ar); 1289 (C=N); 1236 (C-F Ar). **mp:** 295 °C

2-(4-chlorophenyl)quinazolin-4(3H)-one (3c)

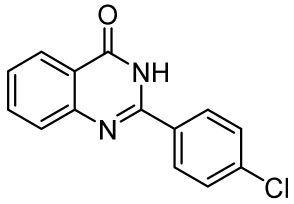

Obtained 5.22 g of **1c** (71%) as white crystals.**^{1}H-NMR** (500 MHz, DMSO-*d*$_6$) δ 12.58 (s, 1H), 8.19 (d, *J* = 8.2 Hz, 2H), 8.14 (d, *J* = 7.9 Hz, 1H), 7.83 (t, *J* = 7.8 Hz, 1H), 7.73 (d, *J* = 8.2 Hz, 1H), 7.61 (d, *J* = 8.2 Hz, 2H), 7.52 (t, *J* = 7.6 Hz, 1H). **^{13}C-NMR** (126 MHz, DMSO) δ 162.1, 151.3, 148.6, 136.3, 134.7, 131.5, 129.6, 128.7, 127.5, 126.8, 125.9, 121.0. **HRMS** (ESI-TOF): m/z calculated for $C_{14}H_{10}ClN_2O$ [M+H]$^+$: m/z 257.0482, found 257.0483. **FTIR (KBr) [cm^{-1}]:** 3179 (N-H); 1678 (C=O); 1603 (C=C ar); 1346 (C-Cl); 1290 (C=N). **mp:** 330 °C

2-(4-bromophenyl)quinazolin-4(3H)-one (3d)

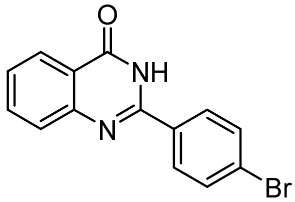

Obtained 6.83 g of **1d** (79%) as white crystals.**^{1}H-NMR** (500 MHz, DMSO-*d*$_6$) δ 12.59 (s, 1H), 8.17 – 8.08 (m, 3H), 7.84 (t, *J* = 7.7 Hz, 1H), 7.78 - 7.72 (t, *J* = 9.1 Hz, 3H), 7.53 (t, *J* = 7.6 Hz, 1H). **^{13}C-NMR** (126 MHz, DMSO) δ 162.1, 151.4, 148.6, 134.7, 131.9, 131.6, 129.8, 127.5, 126.8, 125.9, 125.2, 121.0. **HRMS** (ESI-TOF): m/z calculated for $C_{14}H_{10}BrN_2O$ [M+H]$^+$: m/z 300.9977, found 300.9981. **FTIR (KBr) [cm^{-1}]:** 3181 (N-H); 1677 (C=O); 1602 (C=C ar); 1348 (C-Br); 1311 (C=N). **mp:** 331 °C

2-(4-methoxyphenyl)quinazolin-4(3H)-one (3e)

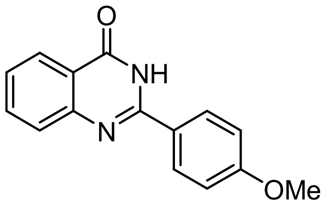

Obtained 4.61 g of **1e** (83%) as white crystals. **^{1}H-NMR** (500 MHz, DMSO-*d*6) δ 12.37 (s, 1H), 8.21 – 8.13 (m, 2H), 8.10 (dd, *J* = 7.8, 1.7 Hz, 1H), 7.78 (ddd, *J* = 8.4, 5.0, 1.5 Hz, 1H), 7.67 (d, *J* = 8.1 Hz, 1H), 7.45 (t, *J* = 7.5 Hz, 1H), 7.11 – 7.00 (m, 2H), 3.82 (d, *J* = 1.3 Hz, 3H). **^{13}C-NMR** (126 MHz, DMSO) δ 162.3, 161.9, 151.8, 148.9, 134.5, 129.4, 127.3, 126.1, 125.8, 124.8, 120.7, 114.0, 55.4. **HRMS** (ESI-TOF): m/z calculated for $C_{15}H_{13}N_2O_2$ [M+H]$^+$: m/z 253.0977, found 253.0978. **FTIR (KBr) [cm^{-1}]:** 3181 (N-H); 1677 (C=O); 1605 (C=C ar); 1253 (C-O). **mp:** 248 °C

Synthesis of 4-chloro-quinazolines (4a-e).

Exemplified for the synthesis of 4-chloro-2-phenylquinazoline **(4a)**

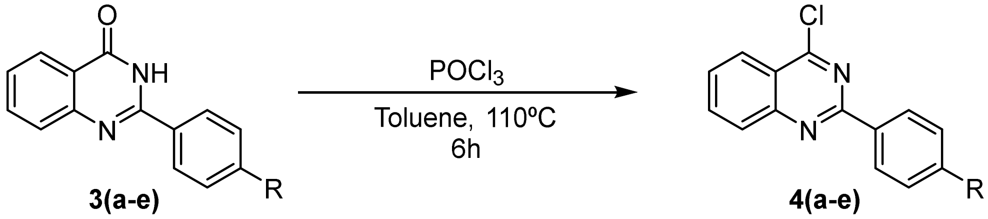

A solution of 2-phenylquinazolin-4(3H)-one **1a** (4.00 g, 18.0 mmol, 1 eq.), 3.3 mL of $POCl_3$ (36.0 mmol, 2 eq.), 5.7 mL of N,N-diethylaniline (36.0 mmol, 2 eq.) in dry toluene (60 mL) was heated to reflux for 6 h. The mixture was cooled to 0 ºC and NH_4Cl(sat) was added carefully. The organic phase was separated, and the resulting aqueous phase was extracted with CH_2Cl_2 (3x20 ml). The mixed organic phases were dried with sodium sulphate, evaporated and purified by flash chromatography (5% EtOAc:Hexanes) to afford 3.95 g (16.6 mmol, 92%) of 4-chloro-2-phenylquinazoline (**2a**) as a white solid. **^{1}H-NMR** (500 MHz, $CDCl_3$) δ 8.62 – 8.55 (m, 2H), 8.25 (dd, *J* = 8.4, 1.4 Hz, 1H), 8.09 (d, *J* = 8.5 Hz, 1H), 7.93 (ddd, *J* = 8.4, 6.9, 1.4 Hz, 1H), 7.66 (ddd, *J* = 8.2, 6.9, 1.2 Hz, 1H), 7.56 – 7.50 (m, 3H). **^{13}C-NMR** (126 MHz, $CDCl_3$) δ 162.7, 160.2, 152.0, 136.8, 135.0, 131.3, 129.1, 128.9, 128.8, 128.4, 126.0, 122.6. **HRMS** (ESI-TOF): m/z calculated for $C_{14}H_{10}FN_2O$ [M+H]$^+$: m/z 240.0454, found 240.0472. **FTIR (KBr) [cm^{-1}]:** 3053 (C-H); 1554 (C=C); 1333 (C=N); 768 (C-Cl). **mp:** 130 °C

4-chloro-2-(4-fluorophenyl)quinazoline (4b)

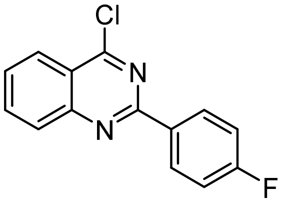

Obtained 3.72 g of **2b** (86%) as a white solid.**^{1}H-NMR** (400 MHz, $CDCl_3$) δ 8.66 – 8.54 (m, 2H), 8.25 (ddd, *J* = 8.4, 1.5, 0.7 Hz, 1H), 8.07 (dt, *J* = 8.4, 1.0 Hz, 1H), 7.94 (ddd, *J* = 8.4, 6.9, 1.4 Hz, 1H), 7.67 (ddd, *J* = 8.2, 6.9, 1.2 Hz, 1H), 7.24 – 7.14 (m, 2H). **^{13}C-NMR** (126 MHz, $CDCl_3$) δ 165.1 (d, *J* = 251.5 Hz), 162.7, 159.3, 152.0, 135.1, 133.0, 133.0, 131.0 (d, *J* = 8.8 Hz), 129.0, 128.4, 126.0, 122.5, 115.8 (d, *J* = 21.7 Hz). **^{19}F-NMR** (376 MHz, $CDCl_3$) δ -109.48. **HRMS** (ESI-TOF): m/z calculated for $C_{14}H_8ClFN_2$ $[M+H]^+$: m/z 258.0360, found 258.0385. **FTIR (KBr) [cm^{-1}]:** 3057 (C-H); 1561 (C=C); 1330 (C=N); 1230 (C-F Ar); 759 (C-Cl). **mp:** 136 °C

4-chloro-2-(4-chlorophenyl)quinazoline (4c)

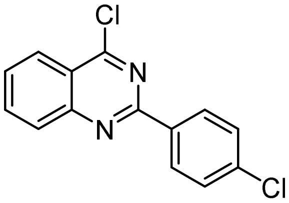

Obtained 3.34 g of **2c** (77%) as a white solid.**^{1}H-NMR** (500 MHz, $CDCl_3$) δ 8.58 – 8.50 (m, 2H), 8.25 (dd, *J* = 8.4, 1.4 Hz, 1H), 8.08 (d, *J* = 8.7 Hz, 1H), 7.94 (ddd, *J* = 8.4, 7.0, 1.4 Hz, 1H), 7.68 (ddd, *J* = 8.2, 6.9, 1.2 Hz, 1H), 7.55 – 7.47 (m, 2H). **^{13}C-NMR** (126 MHz, $CDCl_3$) δ 162.8, 159.2, 152.0, 137.6, 135.3, 135.1, 130.2, 129.0, 128.6, 126.0, 122.7. **HRMS** (ESI-TOF): m/z calculated for $C_{14}H_9Cl_2N_2$ $[M+H]^+$: m/z 275.0143, found 275.0144. **FTIR (KBr) [cm^{-1}]:** 3055 (C-H); 1552 (C=C); 1329 (C=N); 1245 (C-Cl Ar); 759 (C-Cl). **mp:** 168 °C

4-chloro-2-(4-bromophenyl)quinazoline (4d)

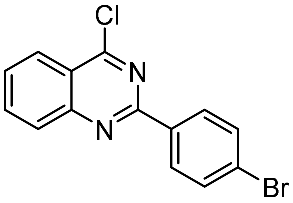

Obtained 2.71 g of **2d** (64%) as a white solid.**^{1}H-NMR** (500 MHz, $CDCl_3$) δ 8.48 – 8.42 (m, 2H), 8.24 (dd, *J* = 8.4, 1.4 Hz, 1H), 8.07 (dt, *J* = 8.4, 0.8 Hz, 1H), 7.93 (ddd, *J* = 8.5, 7.0, 1.4 Hz, 1H), 7.69 – 7.61 (m, 3H). **^{13}C-NMR** (126 MHz, $CDCl_3$) δ 162.8, 159.3, 151.9, 135.7, 135.1, 132.0, 130.4, 129.0, 128.6, 126.2, 126.0, 122.7. **HRMS** (ESI-TOF): m/z calculated for $C_{14}H_9BrClN_2$ $[M+H]^+$: m/z 317.9559, found 317.9589. **FTIR (KBr) [cm^{-1}]:** 3049 (C-H); 1550 (C=C); 1324 (C=N); 1244 (C-Br Ar): 759 (C-Cl). **mp:** 156 °C

4-chloro-2-(4-methoxyphenyl)quinazoline (4e)

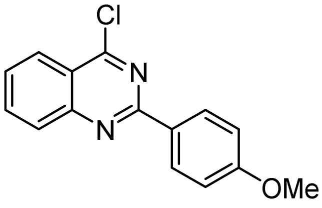

Obtained 2.92 g of **2e** (67%) as a white solid.**^{1}H-NMR** (500 MHz, $CDCl_3$) δ 8.54 (d, *J* = 8.9 Hz, 2H), 8.19 (dd, *J* = 8.5, 1.4 Hz, 1H), 8.02 (d, *J* = 7.9 Hz, 1H), 7.88 (ddd, *J* = 8.4, 6.9, 1.4 Hz, 1H), 7.59 (ddd, *J* = 8.2, 6.9, 1.2 Hz, 1H), 7.02 (d, *J* = 8.9 Hz, 2H), 3.89 (s, 3H). **^{13}C-NMR** (126 MHz, $CDCl_3$) δ 162.4, 162.4, 160.0, 152.1, 134.8, 130.6, 129.45, 128.8, 127.8, 125.9, 122.2, 114.1, 55.5. **HRMS** (ESI-TOF): m/z calculated for $C_{15}H_{12}ClN_2O$ $[M+H]^+$: m/z 271.0638, found 271.0640. **FTIR (KBr) [cm^{-1}]:** 3023 (C-H sp3); 1562 (C=C); 1335 (C=N); 1244 (C-O); 761 (C-Cl). **mp:** 120 °C

Synthesis of N-(3-(1H-imidazol-1-yl)propyl)-quinazolin-4-amines (5a-f).

Exemplified for the synthesis of N-(3-(1H-imidazol-1-yl)propyl)-2-phenylquinazolin-4-amine **(3a)**

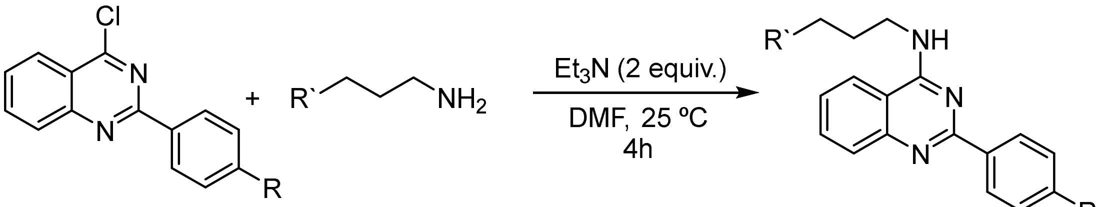

4(a-e) **5(a-e)**

Tor a solution of 4-chloro-2-phenylquinazoline **2a** (1.00 g, 4.2 mmol, 1 eq.) in DMF (10 mL) was added 1.2 mL of Et_3N (2 eq.) and 0.7 mL of 1-(3-aminopropyl)-imidazole (1.3 eq.). The mixture was stirred at 25 ºC for 4 h, and then 40 mL of cold water was poured into the solution. The resulting solid was filtered and crystallized over acetone to afford 1.15 g (3.6 mmol, 85%) of N-(3-(1H-imidazol-1-yl)propyl)-2-phenylquinazolin-4-amine (**3a**) as yellowish crystals. **^{1}H-NMR** (500 MHz, $CDCl_3$) δ 8.61 – 8.47 (m, 2H), 7.95 – 7.89 (m, 1H), 7.78 (dd, *J* = 8.2, 1.3 Hz, 1H), 7.72 (ddd, *J* = 8.4, 6.9, 1.3 Hz, 1H), 7.54 – 7.44 (m, 4H), 7.41 (t, *J* = 7.6 Hz, 1H), 7.10 (s, 1H), 6.98 (s, 1H), 6.46 (s, 1H) 4.11 (t, *J* = 6.7 Hz, 2H), 3.80 (q, *J* = 6.4 Hz, 2H), 2.31 (p, *J* = 6.7 Hz, 2H). **^{13}C-NMR** (126 MHz, $CDCl_3$) δ 160.5, 160.0, 150.7, 139.0, 137.3, 132.8, 130.3, 129.8, 129.0, 128.5, 128.4, 125.6, 121.0, 119.2, 113.9, 45.1, 38.6, 30.7. **HRMS** (ESI-TOF): m/z calculated for $C_{20}H_{20}N_5$ $[M+H]^+$: m/z 330.1719, found 330.1723. **FTIR (KBr) [cm^{-1}]:** 3231 (N-H); 3058 (C-H sp2); 2958 (C-H sp3); 1574 (C=C); 1362 (C=N). **mp:** 178 °C

N-(3-(1H-imidazol-1-yl)propyl)-2-(4-fluorophenyl)quinazolin-4-amine (5b)

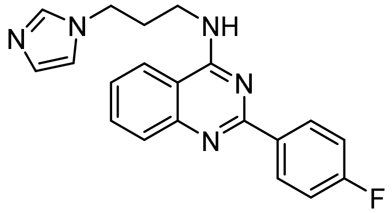

Obtained 0.46 g of **3b** (85%) as yellowish crystals. **^{1}H-NMR** (400 MHz, $CDCl_3$) δ 8.55 – 8.48 (m, 2H), 7.91 (ddd, *J* = 8.4, 1.3, 0.6 Hz, 1H), 7.75 (ddd, *J* = 8.4, 6.9, 1.4 Hz, 1H), 7.62 (dd, *J* = 8.2, 1.1 Hz, 1H), 7.56 (s, 1H), 7.44 (ddd, *J* = 8.2, 7.0, 1.2 Hz, 1H), 7.21 – 7.13 (m, 2H), 7.12 (t, *J* = 1.1 Hz, 1H), 6.99 (t, *J* = 1.3 Hz, 1H), 5.68 (d, *J* = 6.0 Hz, 1H), 4.15 (t, *J* = 6.7 Hz, 2H), 3.85 (q, *J* = 6.7 Hz, 2H), 2.33 (p, *J* = 6.7 Hz, 2H). **^{13}C-NMR** (126 MHz, $CDCl_3$) δ 164.59 (d, *J* = 249.5 Hz), 159.6, 150.7, 137.3, 135.1, 132.9, 130.51 (d, *J* = 8.6 Hz), 130.0, 129.1, 125.8, 120.5, 119.1, 115.33 (d, *J* = 21.4 Hz), 113.6, 45.1, 38.8, 30.8, 29.8. **^{19}F-NMR** (376 MHz, $CDCl_3$) δ -111.35. **HRMS** (ESI-TOF): m/z calculated for $C_{20}H_{19}N_5$ $[M+H]^+$: m/z 348.1624, found 348.1629. **FTIR (KBr) [cm^{-1}]:** 3229 (N-H); 3058 (C-H sp2); 2958 (C-H sp3); 1581 (C=C); 1362 (C=N); 1217(C-F Ar). **mp:** 230 °C

N-(3-(1H-imidazol-1-yl)propyl)-2-(4-chlorophenyl)quinazolin-4-amine (5c)

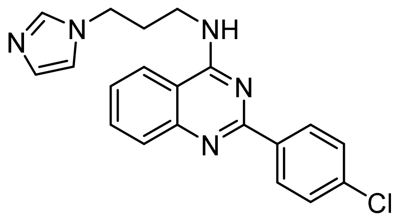

Obtained 0.51 g of **3c** (94%) as yellowish crystals. **^{1}H-NMR** (400 MHz, DMSO-*d*$_6$) δ 8.45 – 8.37 (m, 3H), 8.24 (d, *J* = 8.2 Hz, 1H), 7.82 – 7.72 (m, 2H), 7.70 (s, 1H), 7.58 – 7.47 (m, 3H), 7.25 (s, 1H), 6.95 (s, 1H), 4.13 (t, *J* = 6.7 Hz, 2H), 3.65 (q, *J* = 6.6 Hz, 2H), 2.16 (p, *J* = 6.9 Hz, 2H). **^{13}C-NMR** (101 MHz, DMSO-*d*$_6$) δ 159.7, 158.2, 149.8, 137.4, 134.9, 132.8, 129.7, 129.6, 128.4, 128.3, 127.8, 125.0, 122.8, 119.5, 113.9, 44.0, 38.0, 30.2. **HRMS** (ESI-TOF): m/z calculated for $C_{20}H_{19}ClN_5$ $[M+H]^+$: m/z 364.1329, found 364.1333. **FTIR (KBr) [cm^{-1}]:** 3227 (N-H); 3065 (C-H sp2); 2964 (C-H sp3); 1580 (C=C);1354 (C=N); 1231(C-Cl Ar). **mp:** 220°C

N-(3-(1H-imidazol-1-yl)propyl)-2-(4-bromophenyl)quinazolin-4-amine (5d)

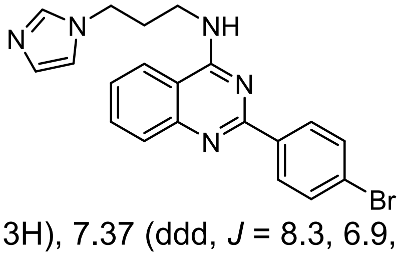

Obtained 0.48 g of **3d** (94%) as yellowish crystals. **^{1}H-NMR** (500 MHz, $CDCl_3$) δ 8.35 – 8.29 (m, 2H), 7.84 (d, *J* = 8.3 Hz, 1H), 7.67 (ddd, *J* = 8.4, 7.0, 1.3 Hz, 1H), 7.62 (d, *J* = 8.2 Hz, 1H), 7.57 – 7.52 (m, 3H), 7.37 (ddd, *J* = 8.3, 6.9, 1.2 Hz, 1H), 7.05 (s, 1H), 6.92 (s, 1H), 5.91 (s, 1H), 4.08 (t, *J* = 6.7 Hz, 2H), 3.76 (q, *J* = 6.5 Hz, 2H), 2.26 (p, *J* = 6.7 Hz, 2H). **^{13}C-**

NMR (126 MHz, $CDCl_3$) δ 159.9, 159.5, 150.6, 137.9, 133.0, 131.6, 130.1, 129.9, 129.1, 126.0, 125.0, 120.7, 119.1, 113.8, 45.1, 38.8, 30.7. **HRMS** (ESI-TOF): m/z calculated for $C_{20}H_{19}BrN_5$ $[M+H]^+$: m/z 408.0824, found 408.0830. **FTIR (KBr) [cm^{-1}]:** 3227 (N-H); 3060 (C-H sp2); 2932 (C-H sp3); 1579 (C=C); 1352 (C=N); 1227(C-Br Ar). **mp:** 204 °C.

N-(3-(1H-imidazol-1-yl)propyl)-2-(4-methoxyphenyl)quinazolin-4-amine (5e)

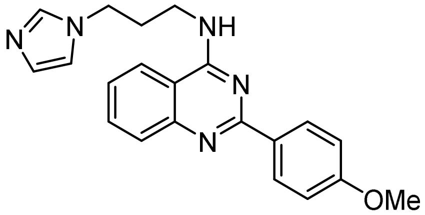

Obtained 0.46 g of **3e** (86%) as yellowish crystals. **^{1}H-NMR** (500 MHz, $CDCl_3$) δ 8.53 – 8.37 (m, 2H), 7.88 (dd, *J* = 8.5, 1.2 Hz, 1H), 7.75 (dd, *J* = 8.3, 1.3 Hz, 1H), 7.69 (ddd, *J* = 8.3, 7.0, 1.3 Hz, 1H), 7.48 (d, *J* = 1.2 Hz, 1H), 7.36 (ddd, *J* = 8.1, 6.9, 1.2 Hz, 1H), 7.10 (d, *J* = 1.1 Hz, 1H), 7.03 – 6.98 (m, 2H), 6.97 (s, 1H), 6.41 (t, *J* = 5.8 Hz, 1H), 4.10 (t, *J* = 6.7 Hz, 2H), 3.88 (s, 3H), 3.77 (q, *J* = 6.5 Hz, 2H), 2.29 (p, *J* = 6.7 Hz, 2H). **^{13}C-NMR** (126 MHz, $CDCl_3$) δ 161.6, 160.3, 159.8, 150.8, 137.2, 132.7, 131.7, 130.0, 129.8, 128.8, 125.2, 121.0, 119.1, 113.8, 113.7, 55.5, 45.0, 38.6, 30.7. **HRMS** (ESI-TOF): m/z calculated for $C_{21}H_{22}N_5O$ $[M+H]^+$: m/z 360.1824, found 360.1830. **FTIR (KBr) [cm^{-1}]:** 3247 (N-H); 3057 (C-H sp2);2935 (C-H sp3); 1582 (C=C); 1354 (C=N); 1243 (C-O Ar). **mp:** 146 °C

2-(4-bromophenyl)-N-butylquinazolin-4-amine (5f)

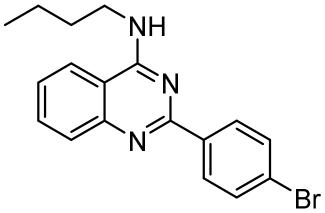

Obtained 0.42 g of **3b** (83%) as yellowish crystals.**^{1}H-NMR** (500 MHz, $CDCl_3$) δ 8.45 (d, *J* = 8.5 Hz, 2H), 7.89 (d, *J* = 8.3 Hz, 1H), 7.72 (ddd, *J* = 8.2, 6.9, 1.4 Hz, 1H), 7.67 (d, *J* = 8.2 Hz, 1H), 7.61 (d, *J* = 8.5 Hz, 2H), 7.42 (ddd, *J* = 8.2, 7.0, 1.2 Hz, 1H), 5.70 (t, *J* = 5.5 Hz, 1H), 3.79 (td, *J* = 7.2, 5.5 Hz, 2H), 1.77 (p, *J* = 7.3 Hz, 2H), 1.52 (h, *J* = 7.4 Hz, 2H), 1.02 (t, *J* = 7.4 Hz, 3H). **^{13}C-NMR** (126 MHz, $CDCl_3$) δ 159.8, 159.8, 150.5, 138.2, 132.7, 131.5, 130.2, 129.1, 125.7, 124.8, 120.5, 113.9, 41.2, 31.7, 20.4, 14.1. **HRMS** (ESI-TOF): m/z calculated for $C_{18}H_{19}BrN_3$ $[M+H]^+$: m/z 356.0762, found 356.0768.**FTIR (KBr) [cm^{-1}]:** 3341 (N-H); 3057 (C-H sp2); 2937 (C-H sp3); 1574 (C=C); 1357 (C=N); 1219(C-Br Ar). **mp:** 120 °C.

Selected Spectra

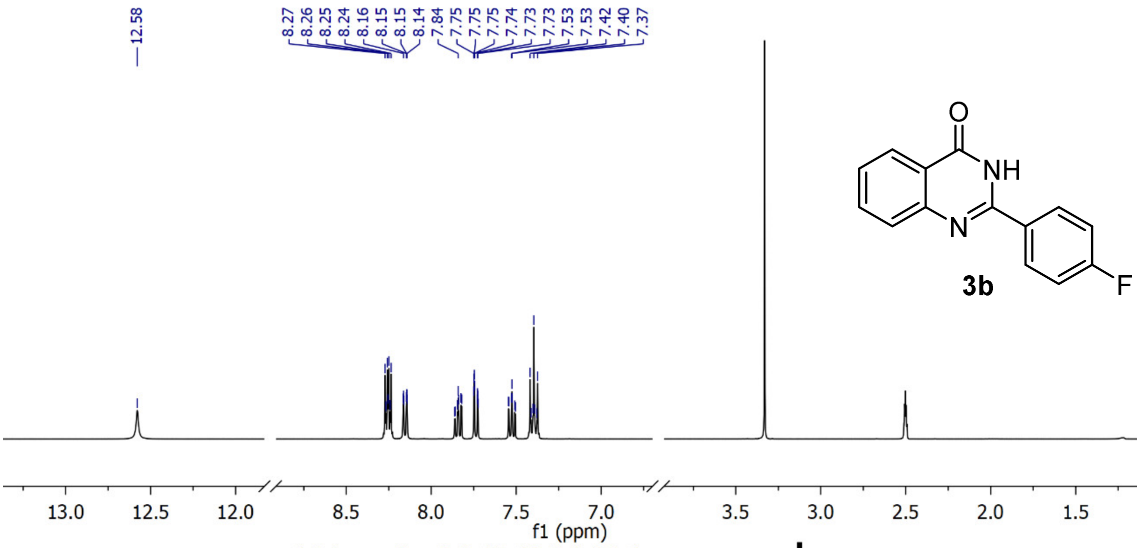

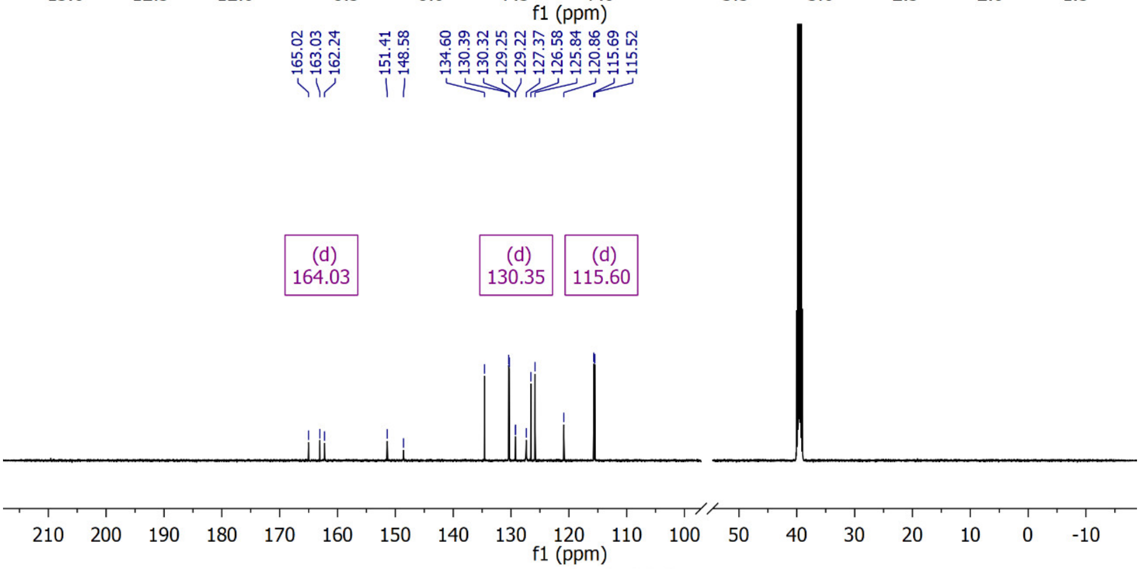

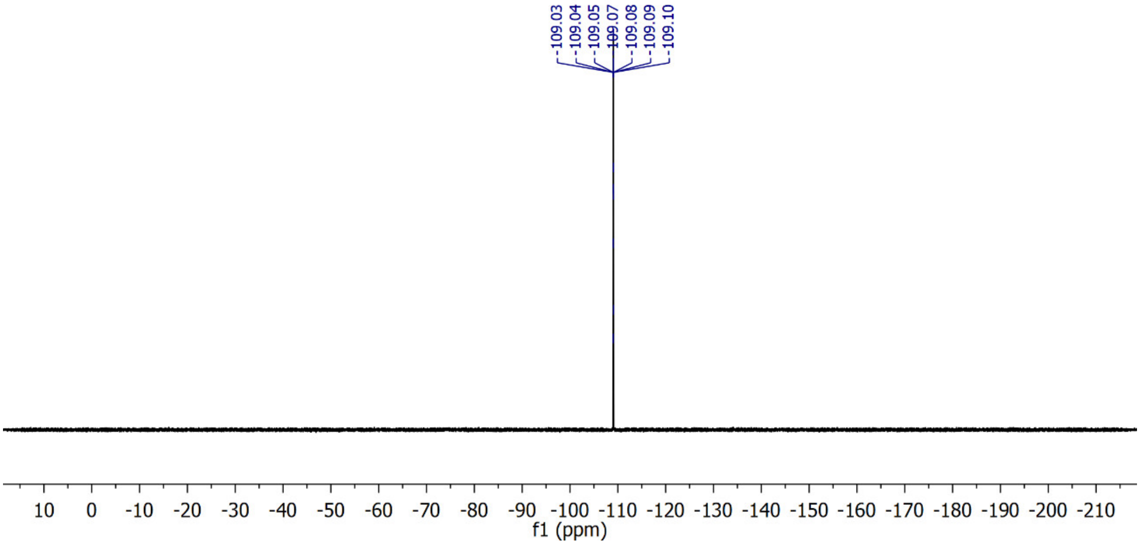

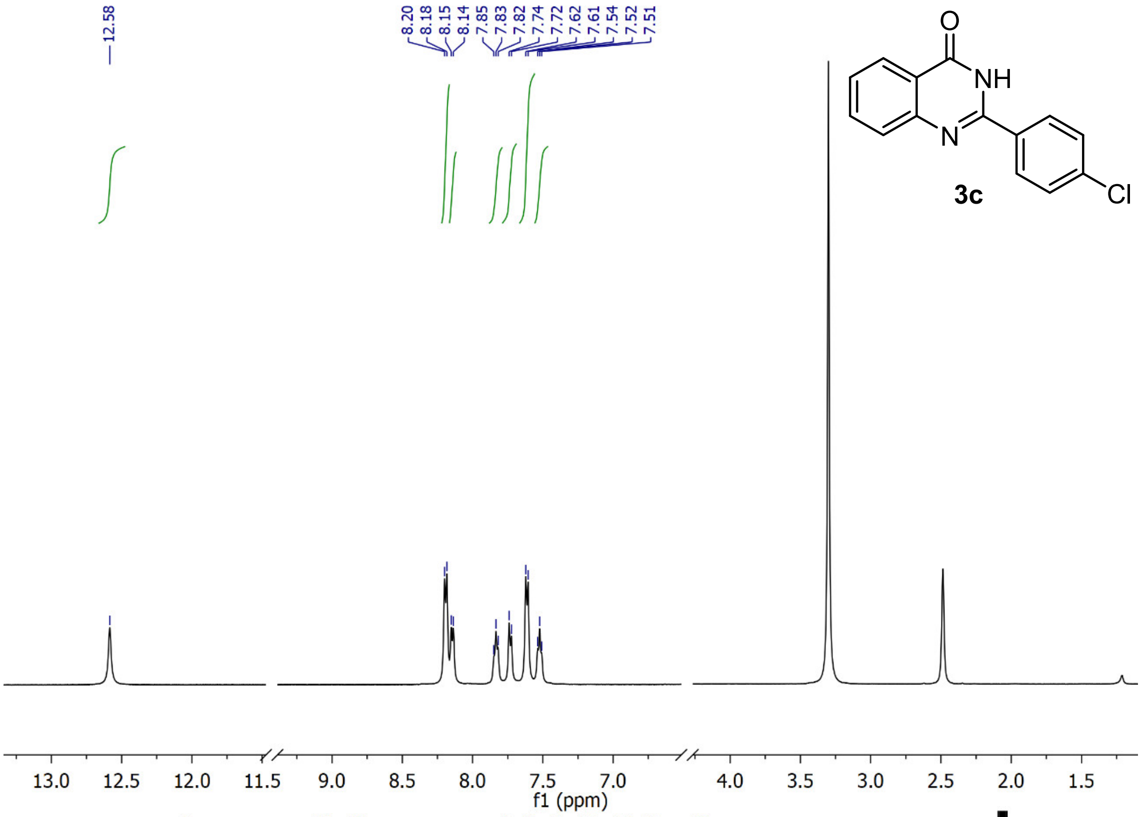
3c
O
NH
N
Cl
12.58
8.20 8.18 8.15 8.14 7.85 7.83 7.82 7.74 7.72 7.62 7.61 7.54 7.52 7.51
13.0 12.5 12.0 11.5 9.0 8.5 8.0 7.5 7.0 4.0 3.5 3.0 2.5 2.0 1.5
f1 (ppm)

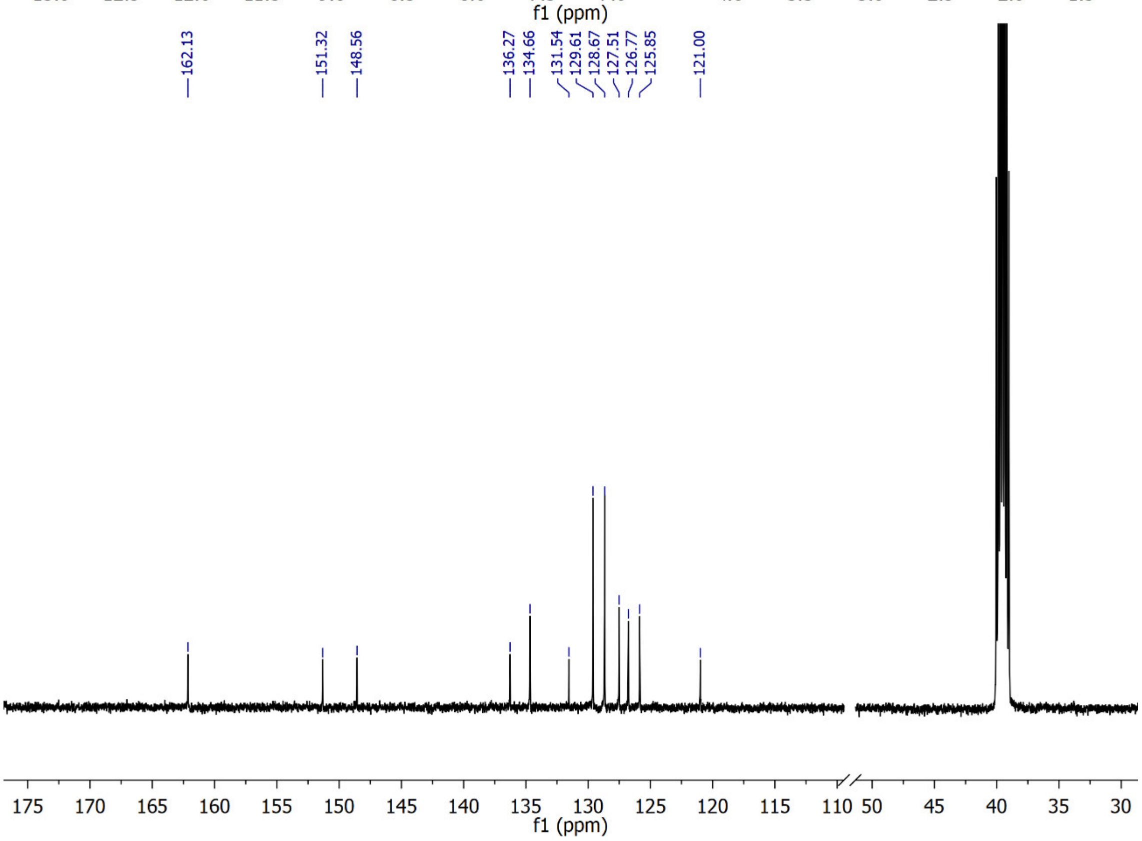
162.13
151.32
148.56
136.27
134.66
131.54 129.61 128.67 127.51 126.77 125.85
121.00
175 170 165 160 155 150 145 140 135 130 125 120 115 110 50 45 40 35 30
f1 (ppm)

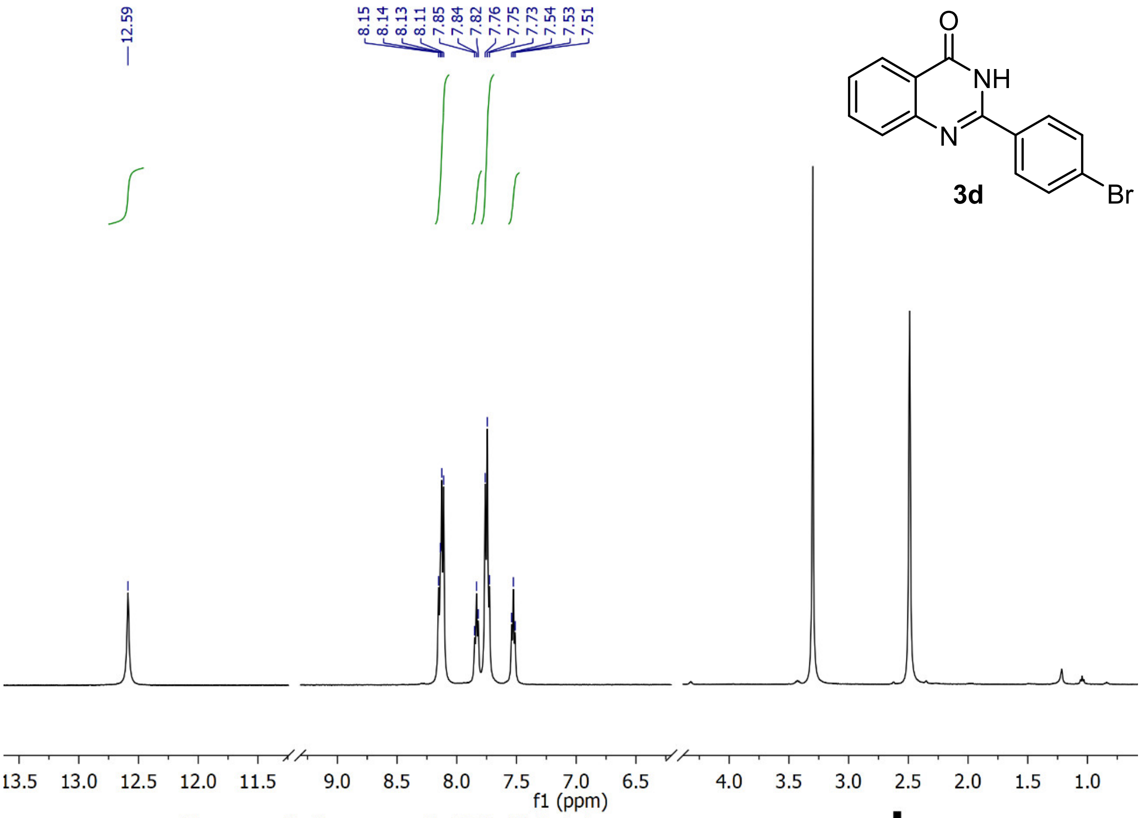

12.59
8.15
8.14
8.13
8.11
7.85
7.84
7.82
7.76
7.75
7.73
7.54
7.53
7.51
O
NH
N
Br
3d
13.5 13.0 12.5 12.0 11.5 9.0 8.5 8.0 7.5 7.0 6.5 4.0 3.5 3.0 2.5 2.0 1.5 1.0
f1 (ppm)

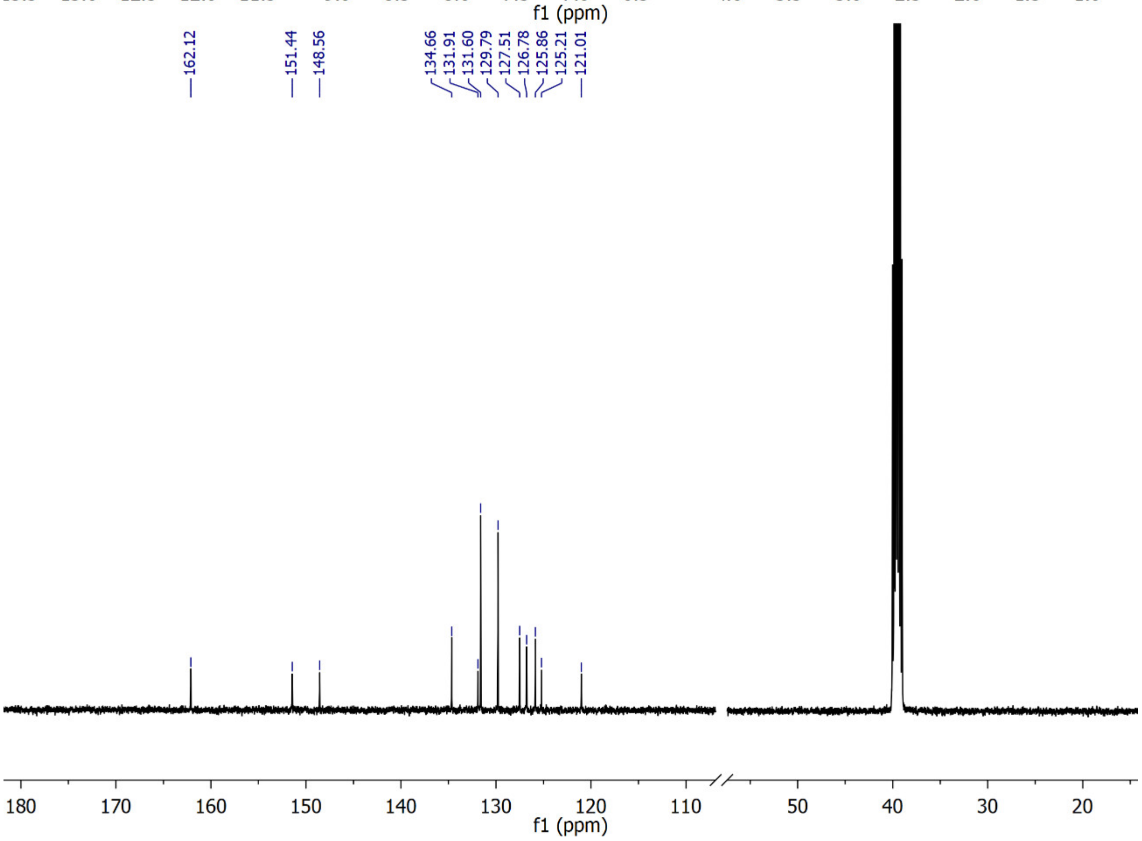

162.12
151.44
148.56
134.66
131.91
131.60
129.79
127.51
126.78
125.86
125.21
121.01
180 170 160 150 140 130 120 110 50 40 30 20
f1 (ppm)

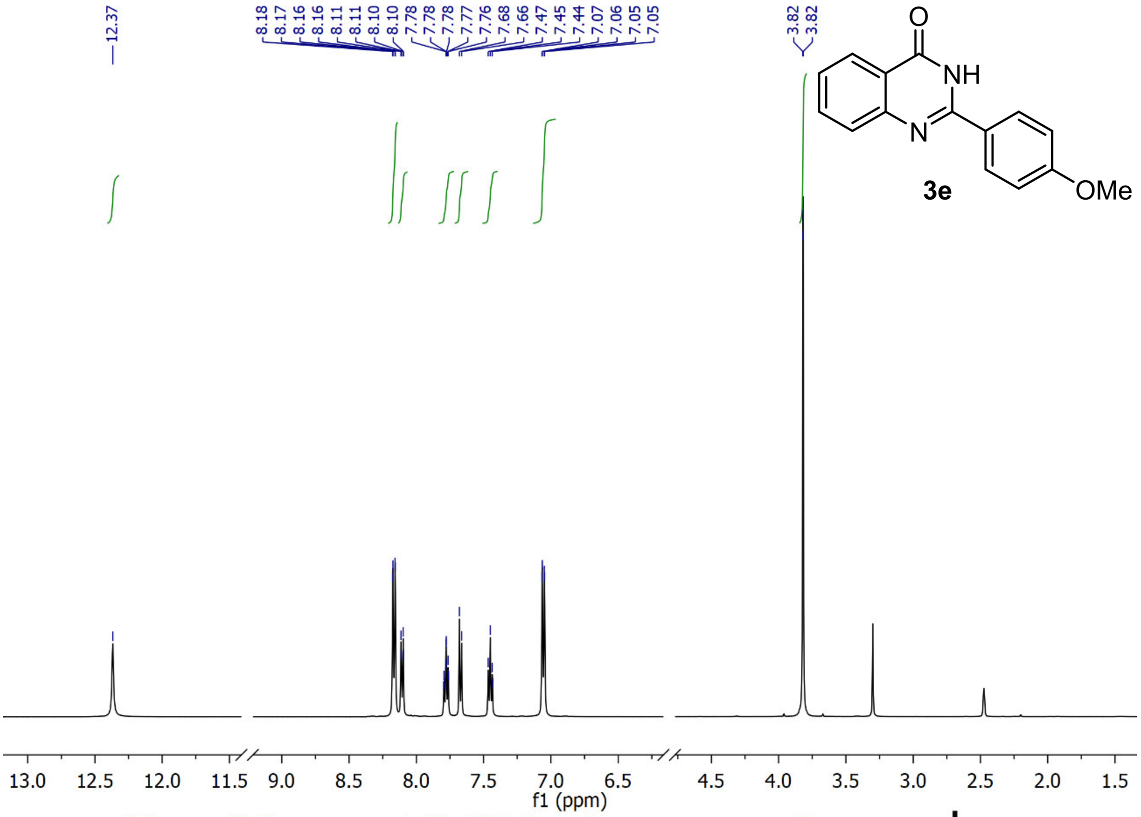
12.37
8.18 8.17 8.16 8.16 8.11 8.11 8.10 8.10 7.78 7.78 7.78 7.77 7.76 7.68 7.66 7.47 7.45 7.44 7.07 7.06 7.05 7.05
3.82 3.82
O
NH
N
OMe
3e
13.0 12.5 12.0 11.5 9.0 8.5 8.0 7.5 7.0 6.5 4.5 4.0 3.5 3.0 2.5 2.0 1.5
f1 (ppm)

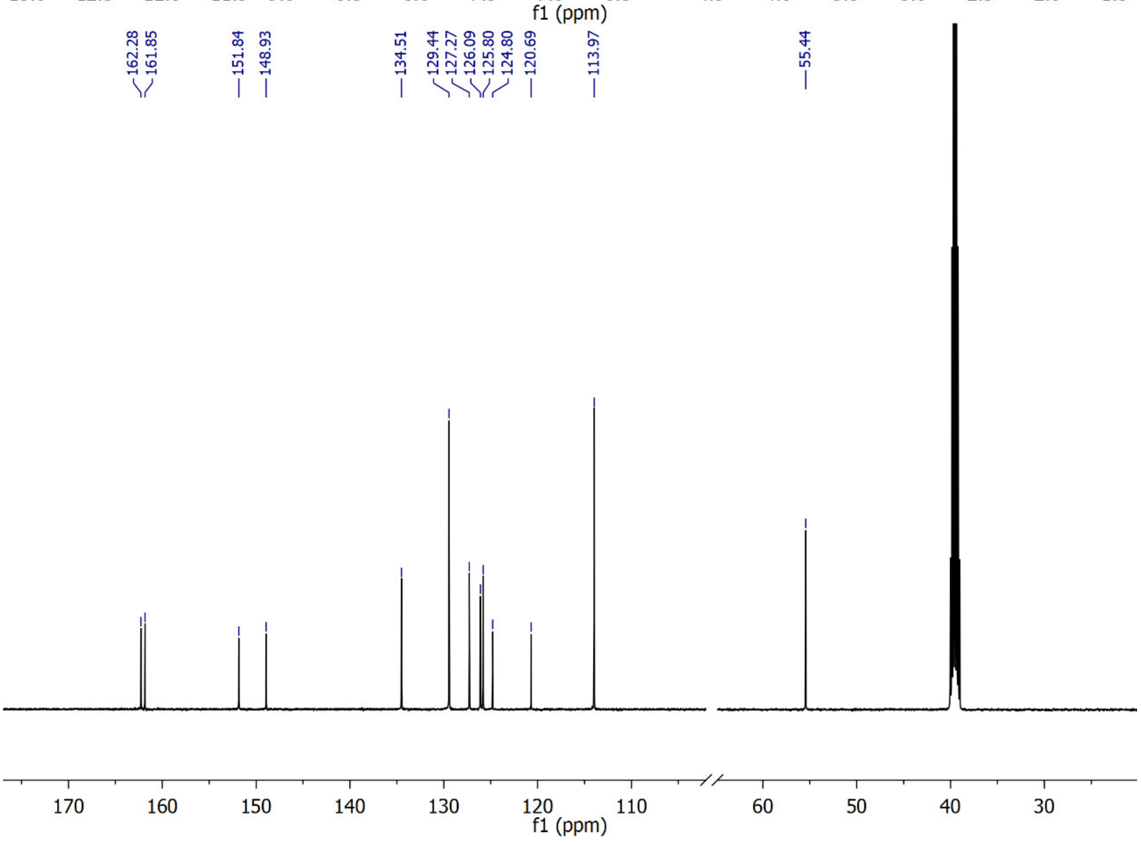
162.28 161.85
151.84
148.93
134.51
129.44 127.27 126.09 125.80 124.80
120.69
113.97
55.44
170 160 150 140 130 120 110 60 50 40 30
f1 (ppm)

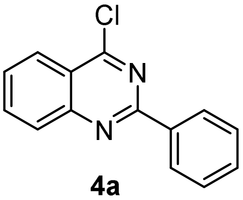
Cl
N
N

4a

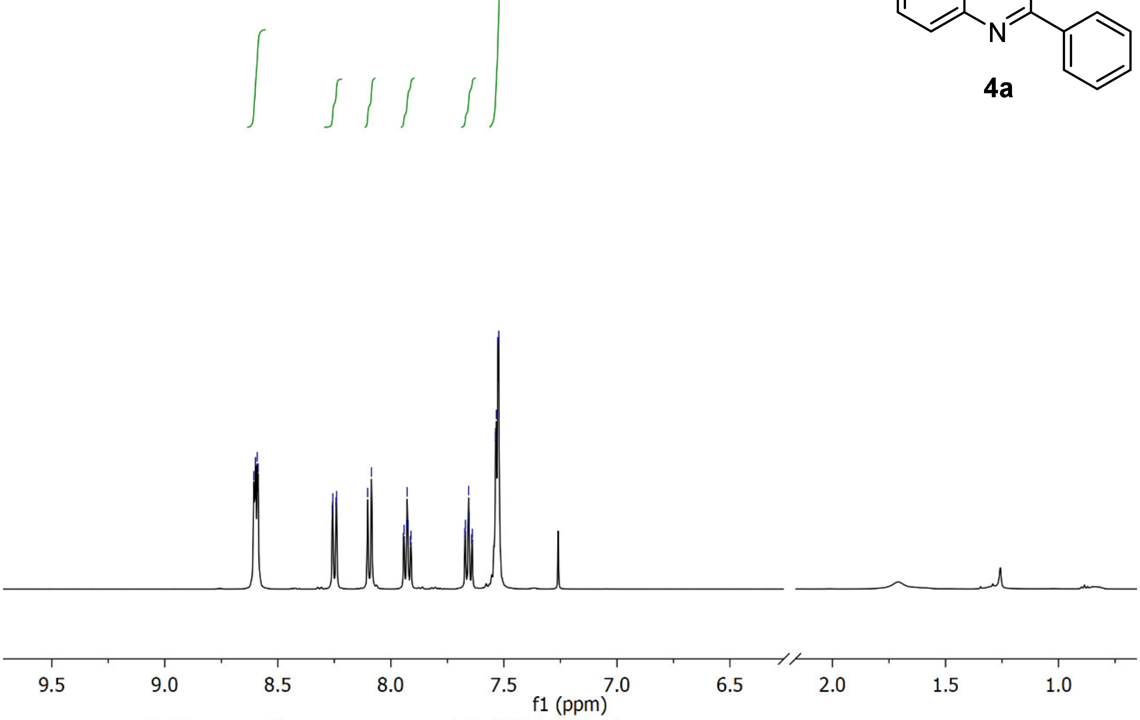
8.61
8.60
8.59
8.59
8.26
8.26
8.24
8.24
8.10
8.09
7.94
7.94
7.93
7.93
7.92
7.91
7.91
7.67
7.67
7.66
7.66
7.65
7.64
7.64
7.54
7.54
7.53
7.53
7.52
9.5 9.0 8.5 8.0 7.5 7.0 6.5 2.0 1.5 1.0
f1 (ppm)

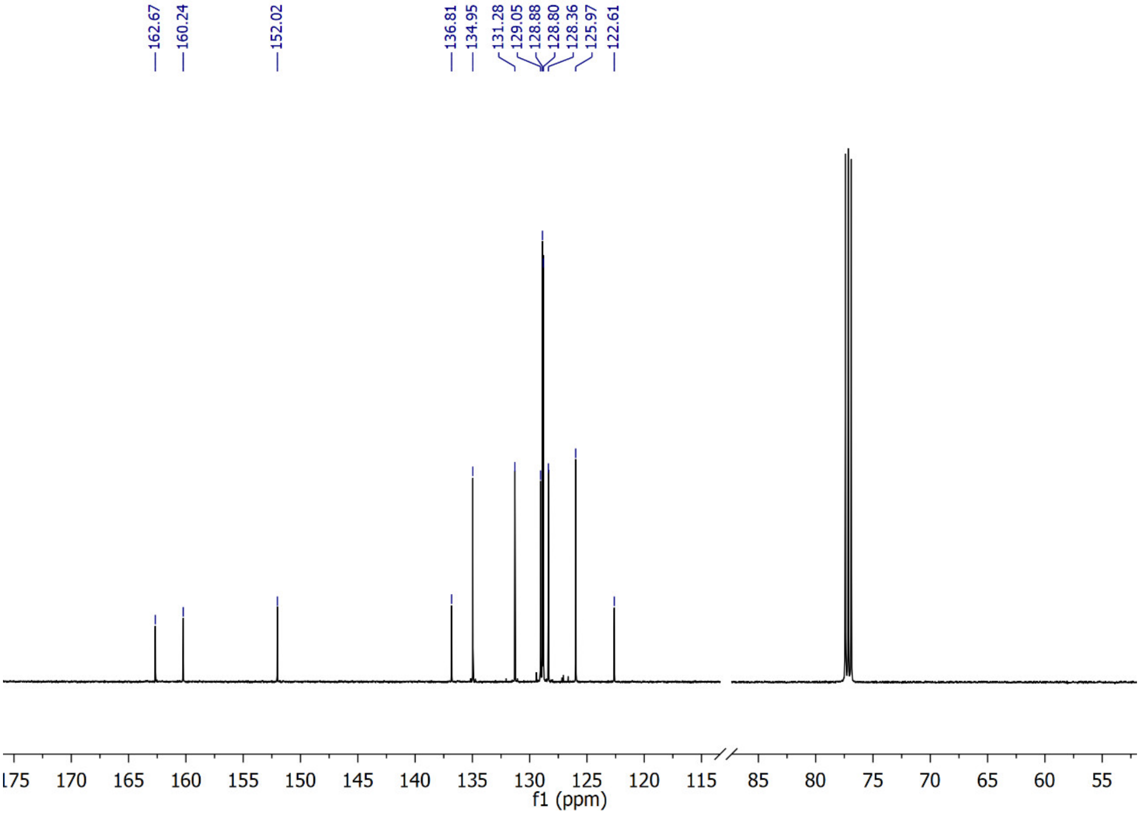
162.67
160.24
152.02
136.81
134.95
131.28
129.05
128.88
128.80
128.36
125.97
122.61
175 170 165 160 155 150 145 140 135 130 125 120 115 85 80 75 70 65 60 55
f1 (ppm)

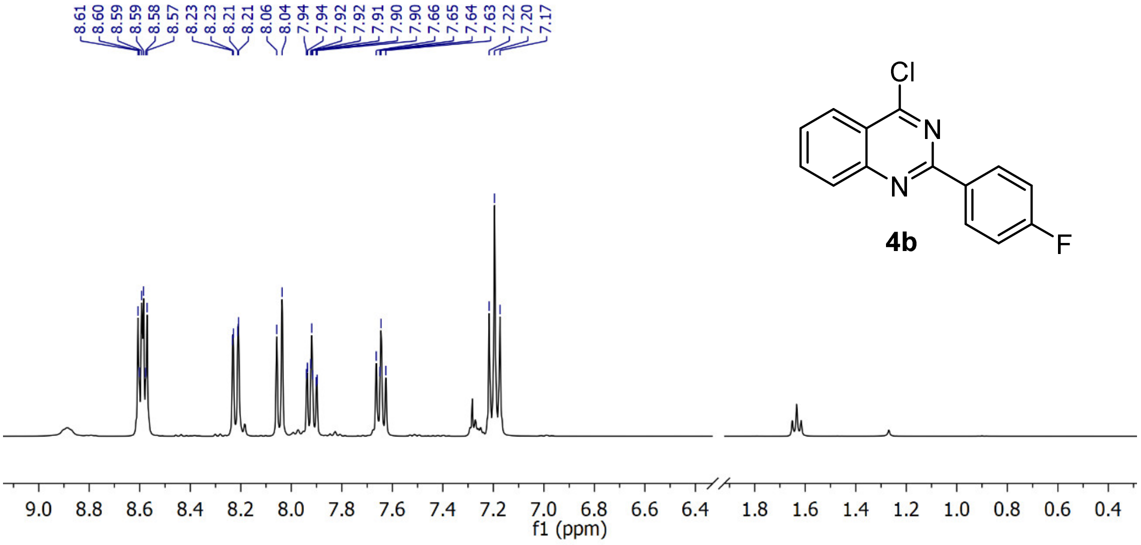
8.61
8.60
8.59
8.59
8.58
8.57
8.23
8.23
8.21
8.21
8.06
8.04
7.94
7.94
7.92
7.92
7.91
7.90
7.90
7.66
7.65
7.64
7.63
7.22
7.20
7.17
f1 (ppm)
Cl
N
N
F

4b

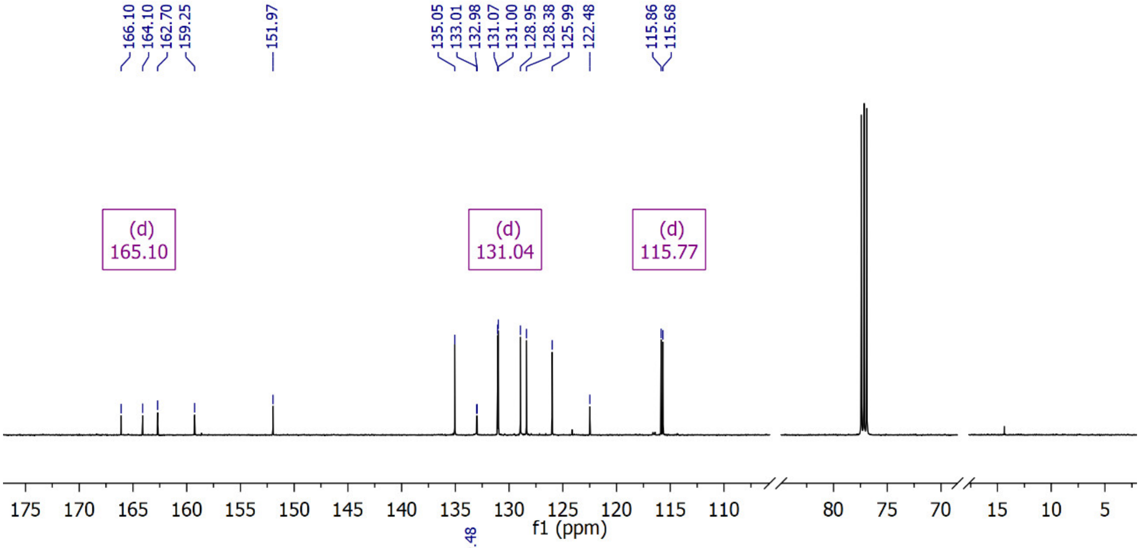
166.10
164.10
162.70
159.25
151.97
135.05
133.01
132.98
131.07
131.00
128.95
128.38
125.99
122.48
115.86
115.68
(d)
165.10
(d)
131.04
(d)
115.77
f1 (ppm)

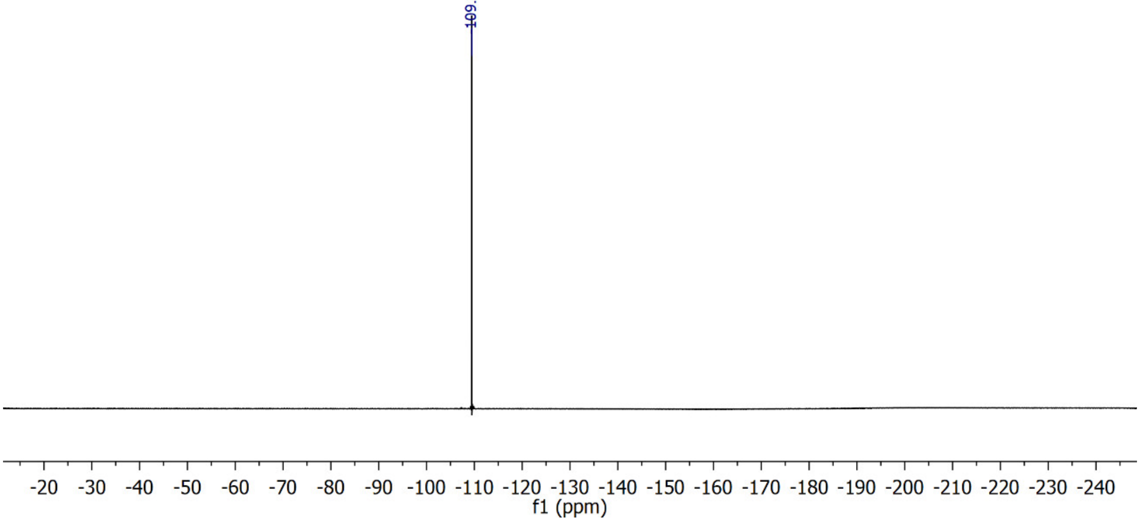
-109.48
f1 (ppm)

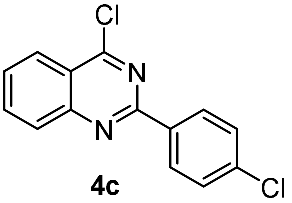
Cl
N
N
Cl

4c

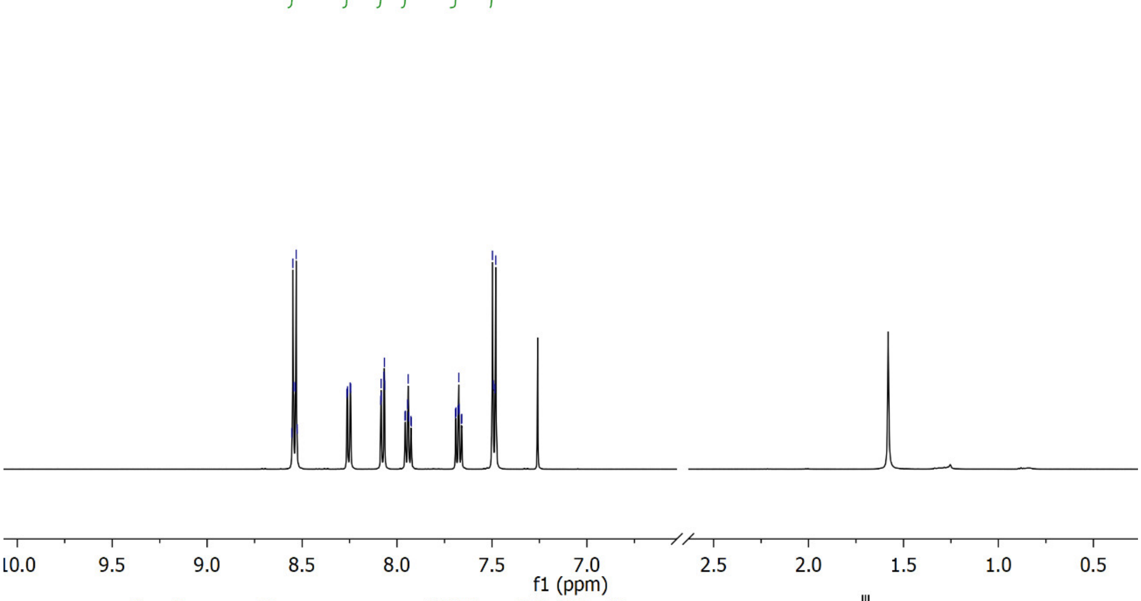
8.55
8.55
8.54
8.54
8.53
8.53
8.26
8.26
8.25
8.24
8.09
8.08
8.08
8.07
8.07
8.07
7.96
7.96
7.94
7.94
7.94
7.93
7.92
7.69
7.69
7.68
7.68
7.67
7.66
7.66
7.50
7.49
7.48
7.48
10.0
9.5
9.0
8.5
8.0
7.5
7.0
2.5
2.0
1.5
1.0
0.5
f1 (ppm)

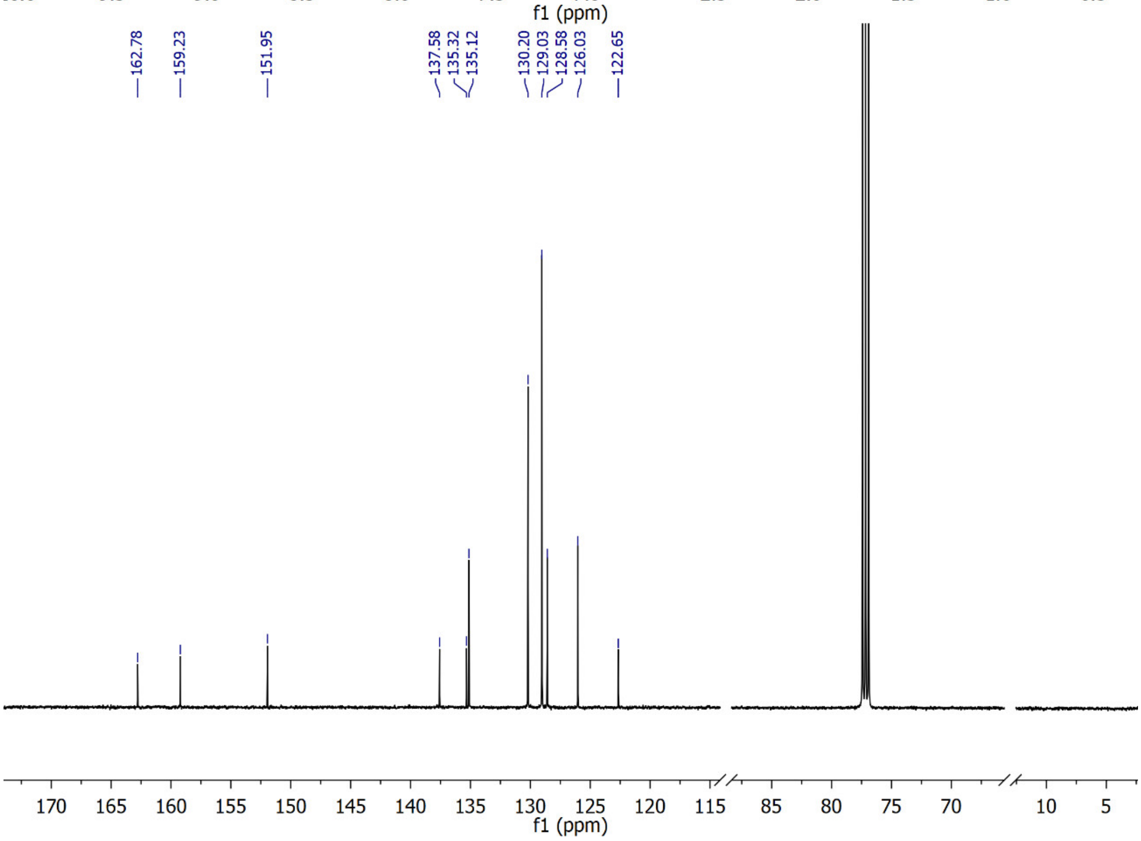
162.78
159.23
151.95
137.58
135.32
135.12
130.20
129.03
128.58
126.03
122.65
170
165
160
155
150
145
140
135
130
125
120
115
85
80
75
70
10
5
f1 (ppm)

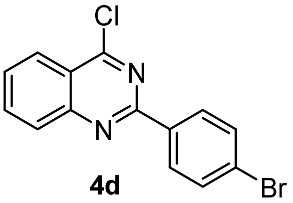
Cl
N
N
Br

4d

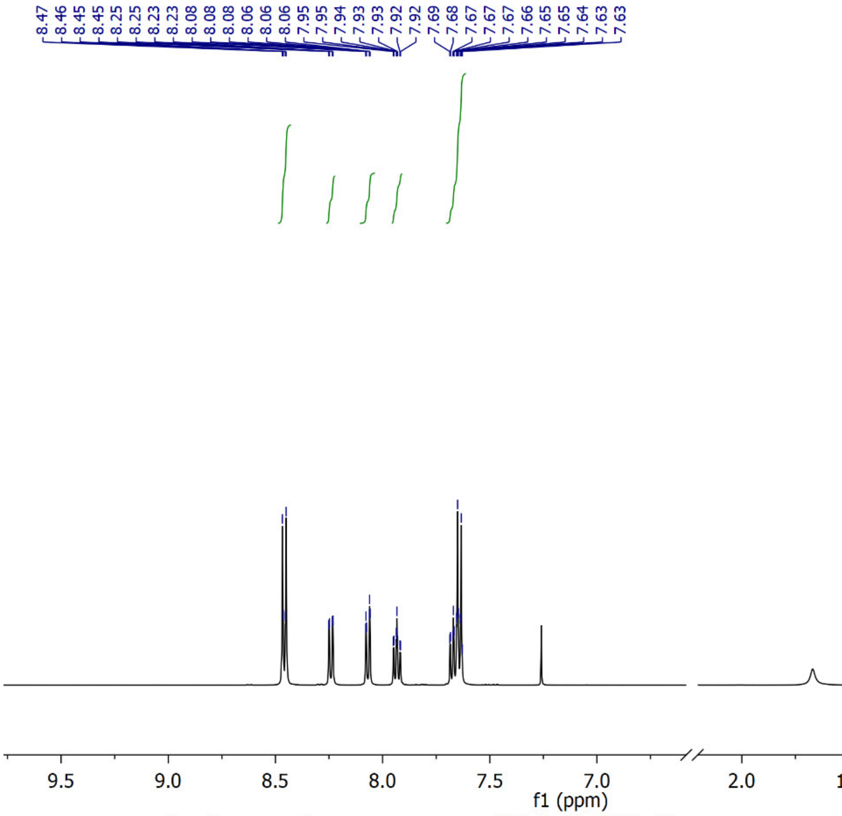
8.47
8.46
8.45
8.45
8.25
8.25
8.23
8.23
8.08
8.08
8.08
8.06
8.06
7.95
7.95
7.94
7.93
7.93
7.92
7.92
7.69
7.68
7.67
7.67
7.67
7.66
7.65
7.65
7.64
7.63
7.63
9.5
9.0
8.5
8.0
7.5
7.0
2.0
1.5
1.0
0.5
f1 (ppm)

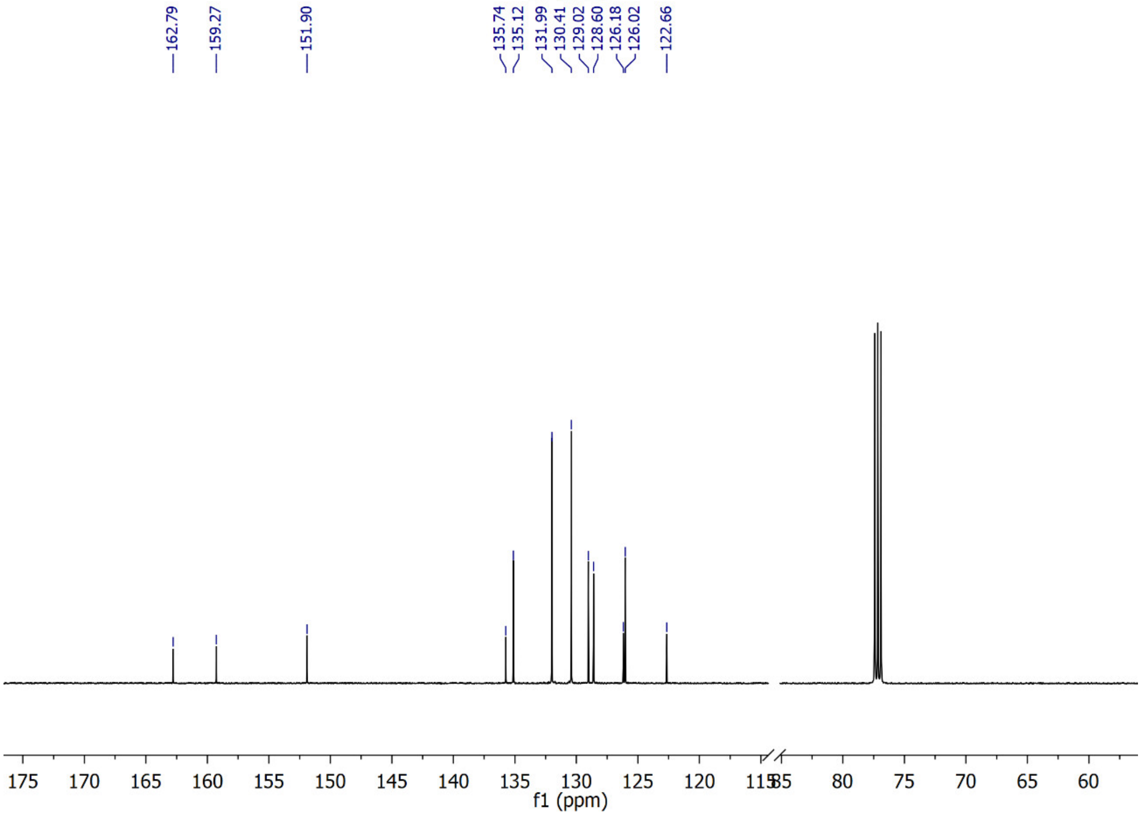
162.79
159.27
151.90
135.74
135.12
131.99
130.41
129.02
128.60
126.18
126.02
122.66
175
170
165
160
155
150
145
140
135
130
125
120
115
85
80
75
70
65
60
f1 (ppm)

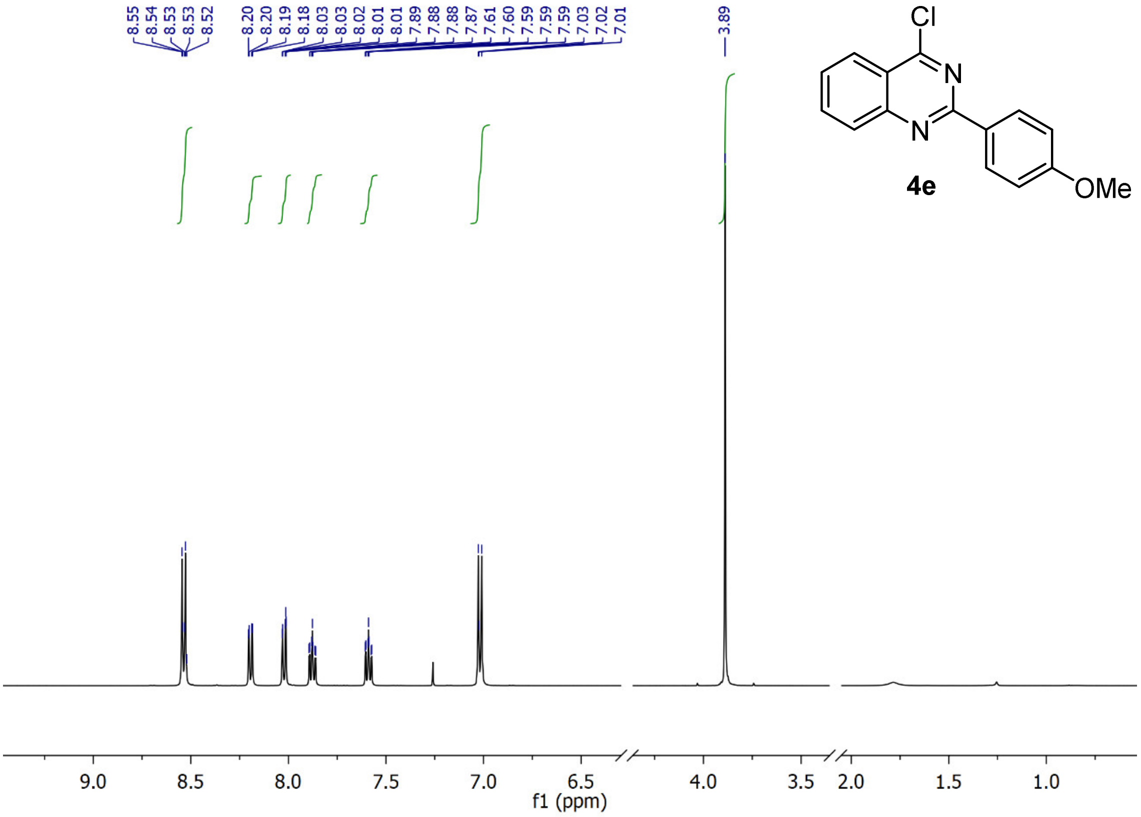
8.55
8.54
8.53
8.53
8.52
8.20
8.20
8.19
8.18
8.03
8.03
8.02
8.01
8.01
7.89
7.88
7.88
7.87
7.61
7.60
7.59
7.59
7.59
7.03
7.02
7.01
3.89
Cl
N
N
OMe
4e
9.0
8.5
8.0
7.5
7.0
6.5
4.0
3.5
2.0
1.5
1.0
f1 (ppm)

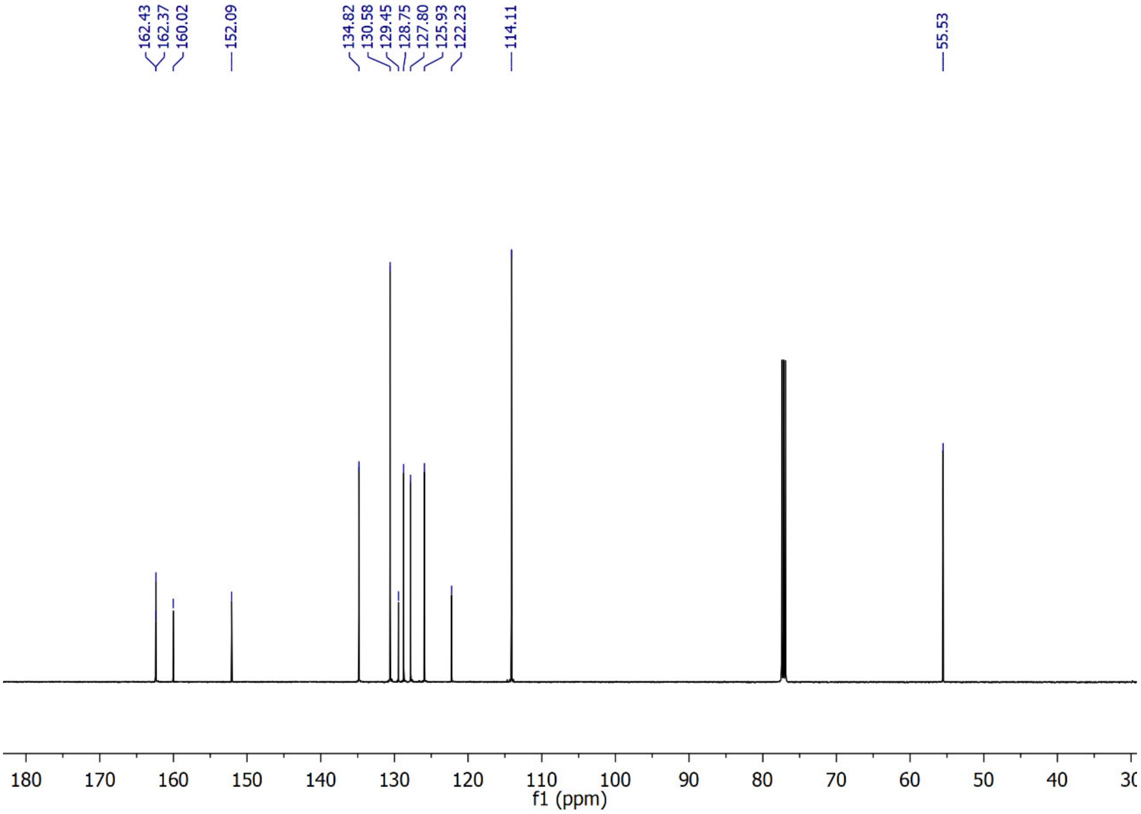
162.43
162.37
160.02
152.09
134.82
130.58
129.45
128.75
127.80
125.93
122.23
114.11
55.53
180
170
160
150
140
130
120
110
100
90
80
70
60
50
40
30
f1 (ppm)

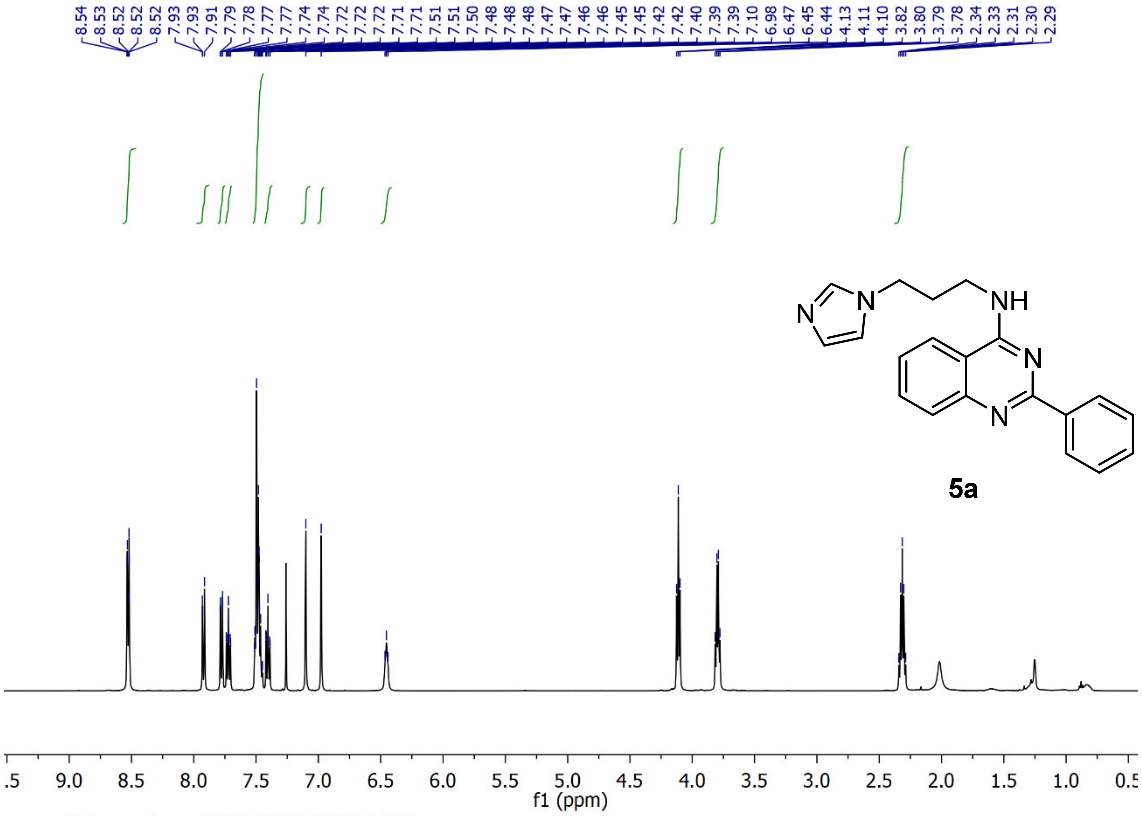
NH
N
N
N
N
5a
f1 (ppm)

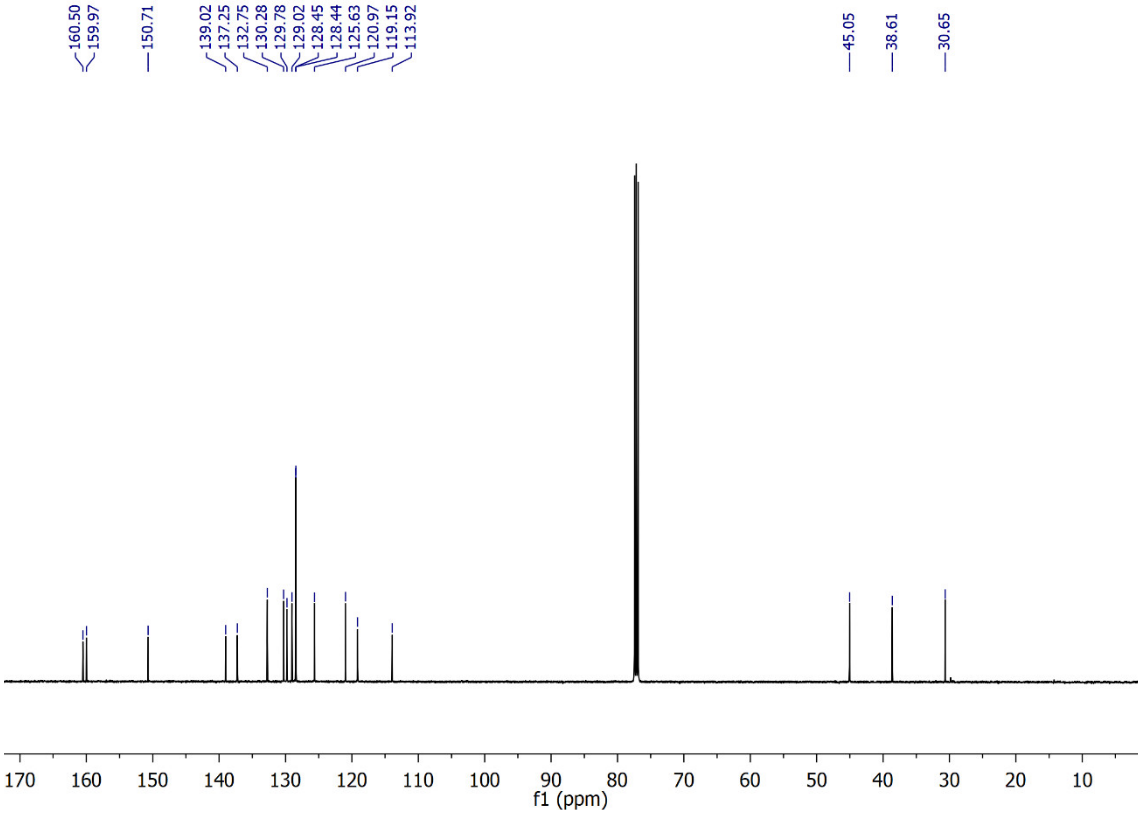
f1 (ppm)

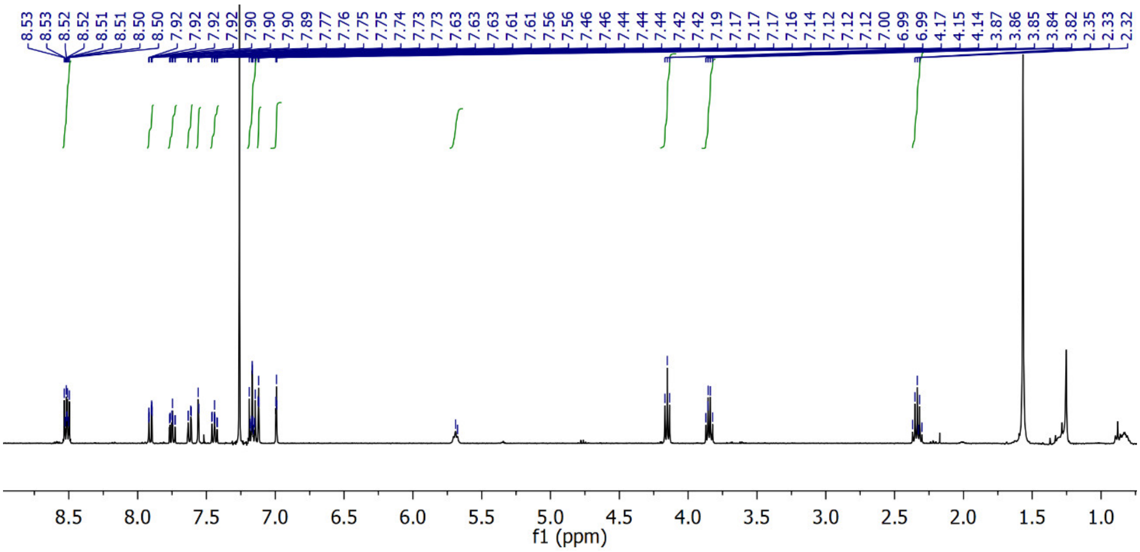
8.53 8.53 8.52 8.52 8.51 8.51 8.50 8.50 7.92 7.92 7.92 7.92 7.92 7.90 7.90 7.90 7.89 7.77 7.76 7.75 7.75 7.74 7.73 7.73 7.63 7.63 7.63 7.61 7.61 7.56 7.56 7.46 7.46 7.44 7.44 7.44 7.42 7.42 7.19 7.17 7.17 7.17 7.16 7.14 7.12 7.12 7.12 7.00 6.99 4.17 4.15 4.14 3.87 3.86 3.85 3.84 3.82 2.35 2.33 2.32
f1 (ppm)

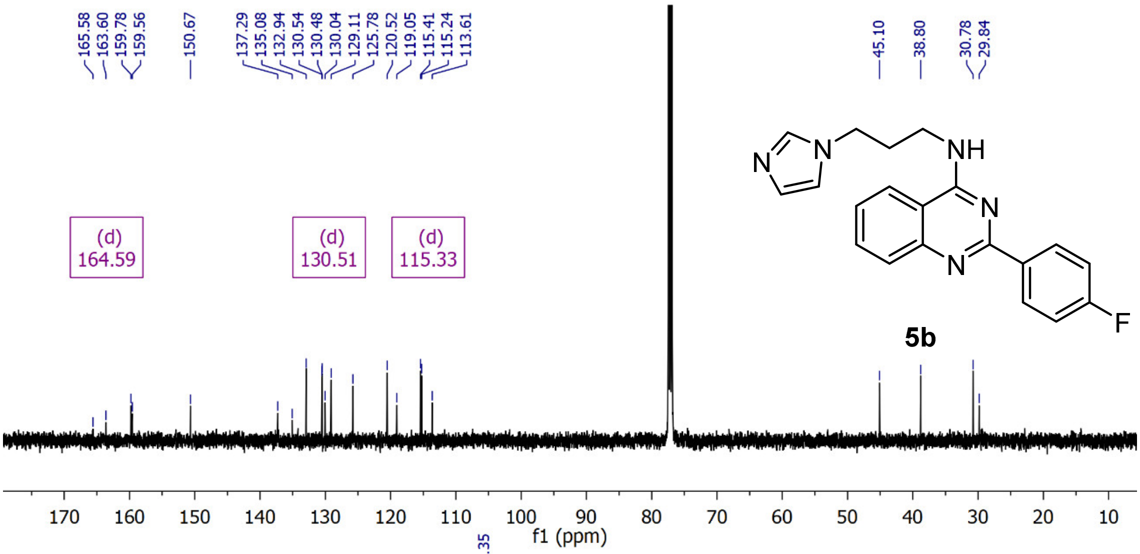
165.58 163.60 159.78 159.56 150.67 137.29 135.08 132.94 130.54 130.48 130.04 129.11 125.78 120.52 119.05 115.41 115.24 113.61
45.10 38.80 30.78 29.84
(d) 164.59
(d) 130.51
(d) 115.33
NH
N
N
N
N
F
5b
f1 (ppm)

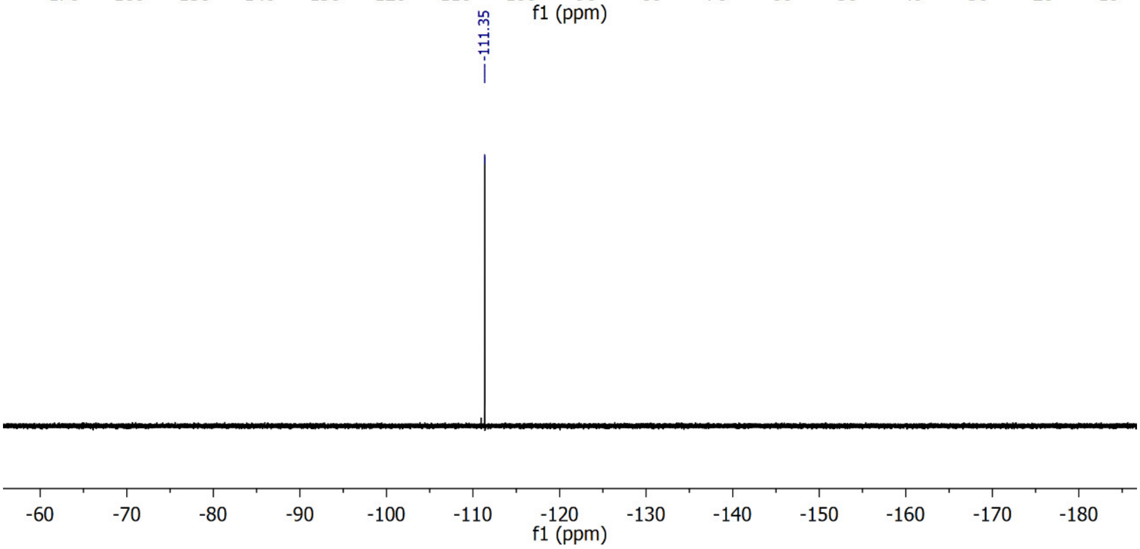
-111.35
f1 (ppm)

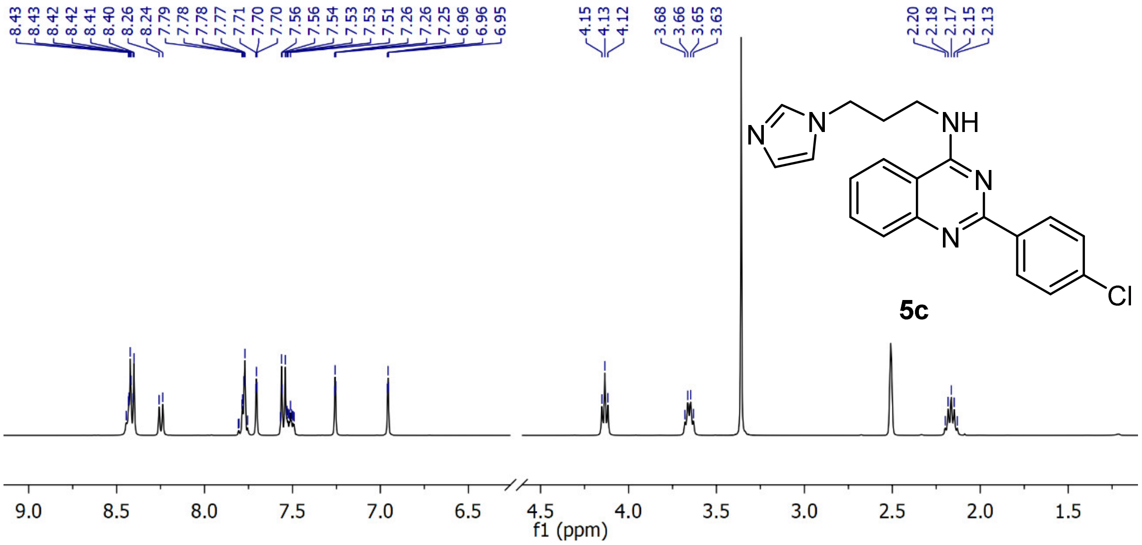
8.43
8.43
8.42
8.42
8.41
8.40
8.26
8.24
7.79
7.78
7.78
7.77
7.71
7.70
7.70
7.56
7.56
7.54
7.53
7.53
7.51
7.26
7.26
7.25
6.96
6.96
6.95
4.15
4.13
4.12
3.68
3.66
3.65
3.63
2.20
2.18
2.17
2.15
2.13
N
N
NH
N
N
Cl
5c
9.0
8.5
8.0
7.5
7.0
6.5
4.5
4.0
3.5
3.0
2.5
2.0
1.5
f1 (ppm)

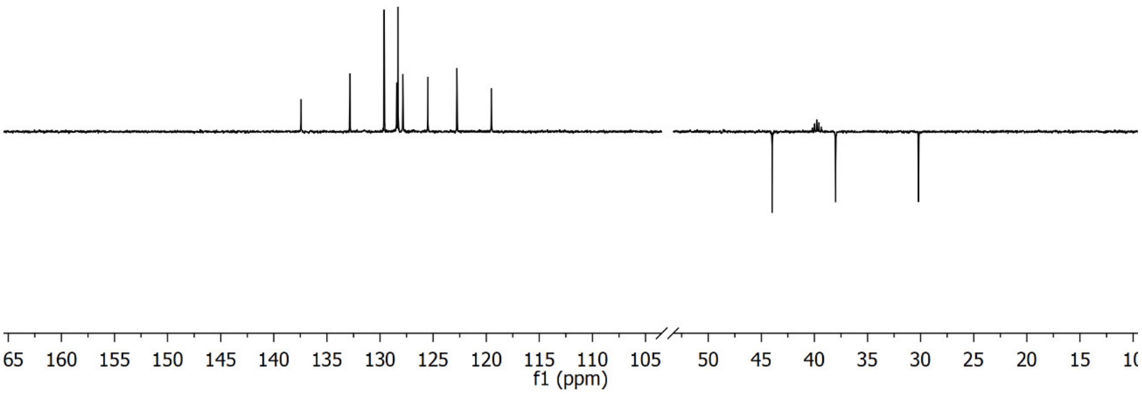
65
160
155
150
145
140
135
130
125
120
115
110
105
50
45
40
35
30
25
20
15
1(
f1 (ppm)

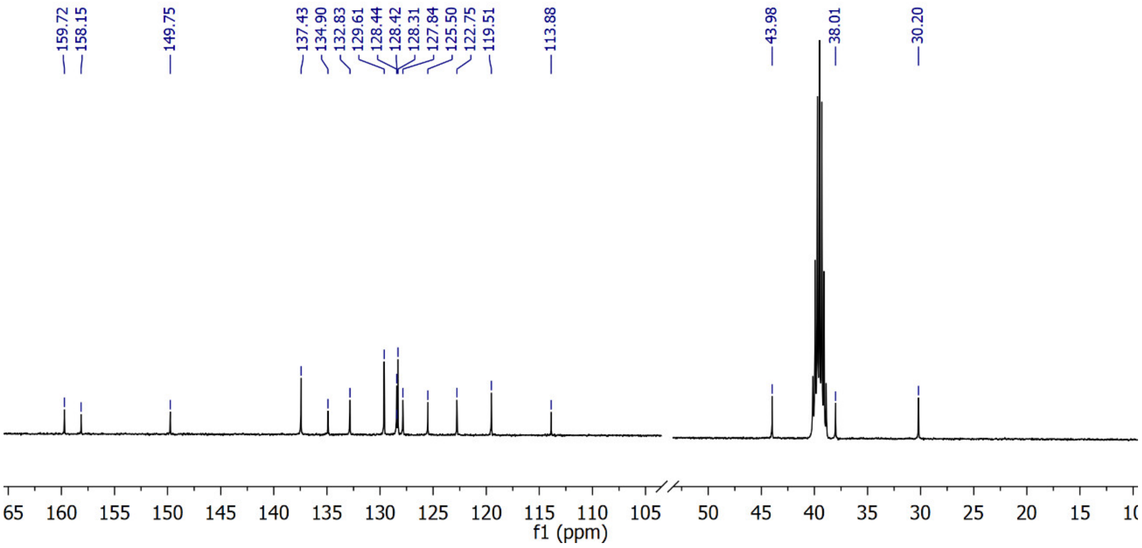
159.72
158.15
149.75
137.43
134.90
132.83
129.61
128.44
128.42
128.31
127.84
125.50
122.75
119.51
113.88
43.98
38.01
30.20
65
160
155
150
145
140
135
130
125
120
115
110
105
50
45
40
35
30
25
20
15
1(
f1 (ppm)

HMBC spectra of compound **5c**.

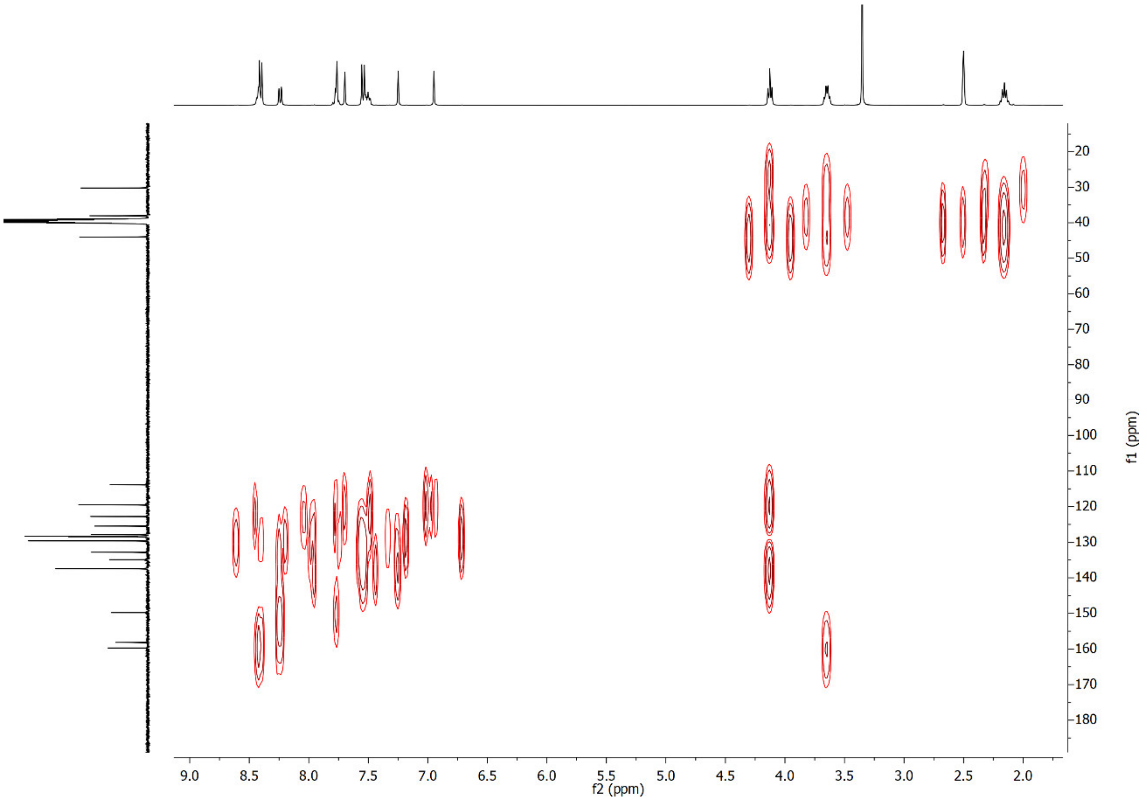

HSQC spectra of compound **5c**.

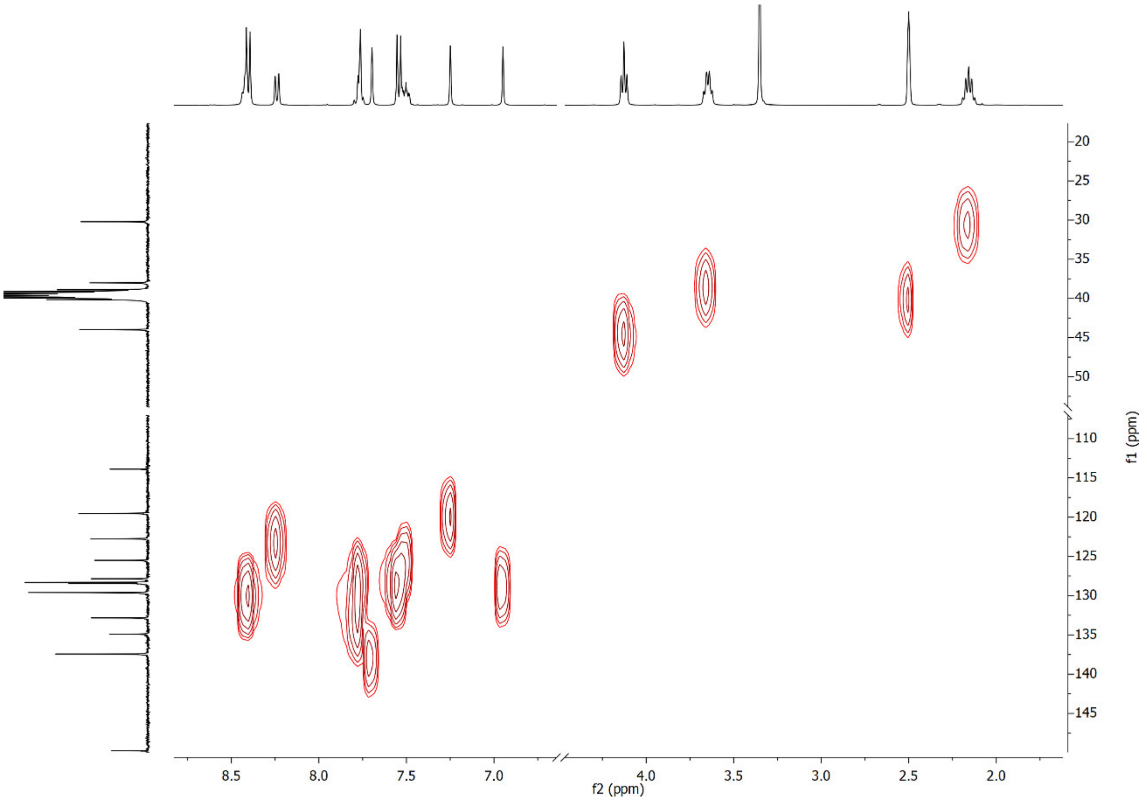

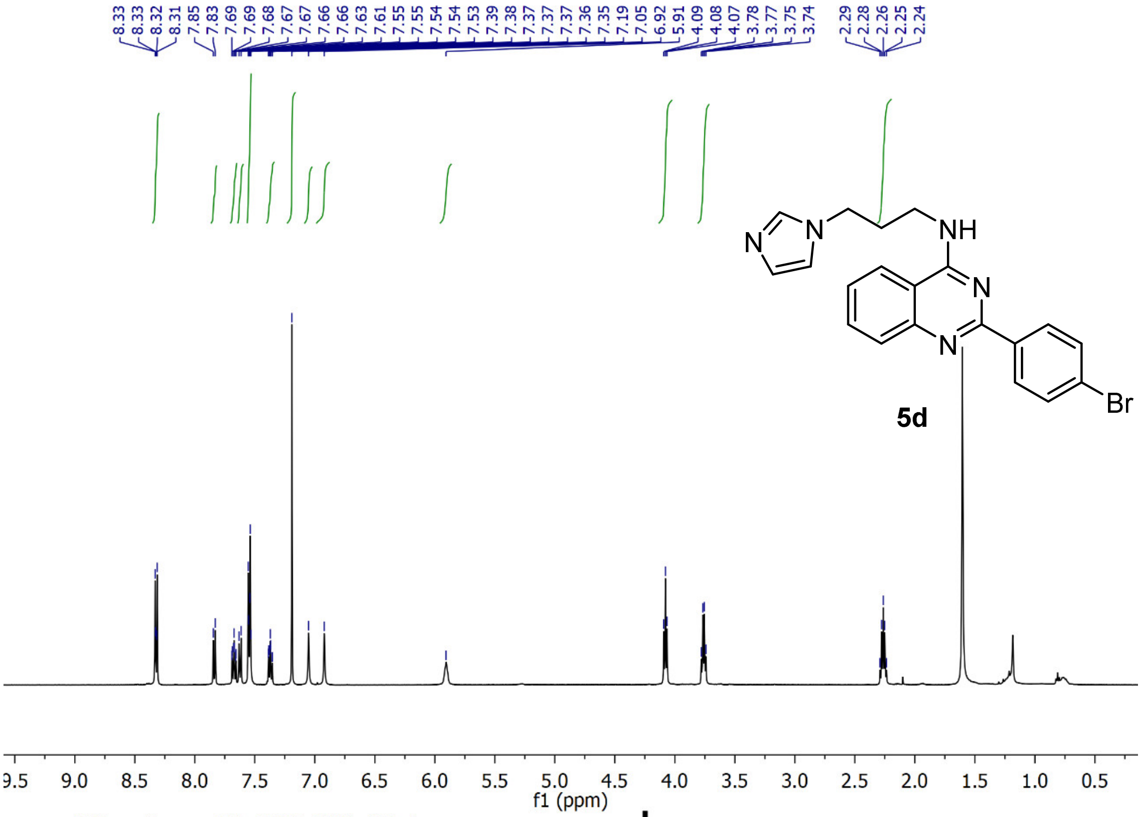
8.33
8.33
8.32
8.31
7.85
7.83
7.69
7.69
7.68
7.67
7.67
7.66
7.66
7.63
7.61
7.55
7.55
7.54
7.54
7.53
7.39
7.38
7.37
7.37
7.37
7.36
7.35
7.19
7.05
6.92
5.91
4.09
4.08
4.07
3.78
3.77
3.75
3.74
2.29
2.28
2.26
2.25
2.24
N
N
NH
N
N
Br
5d
f1 (ppm)

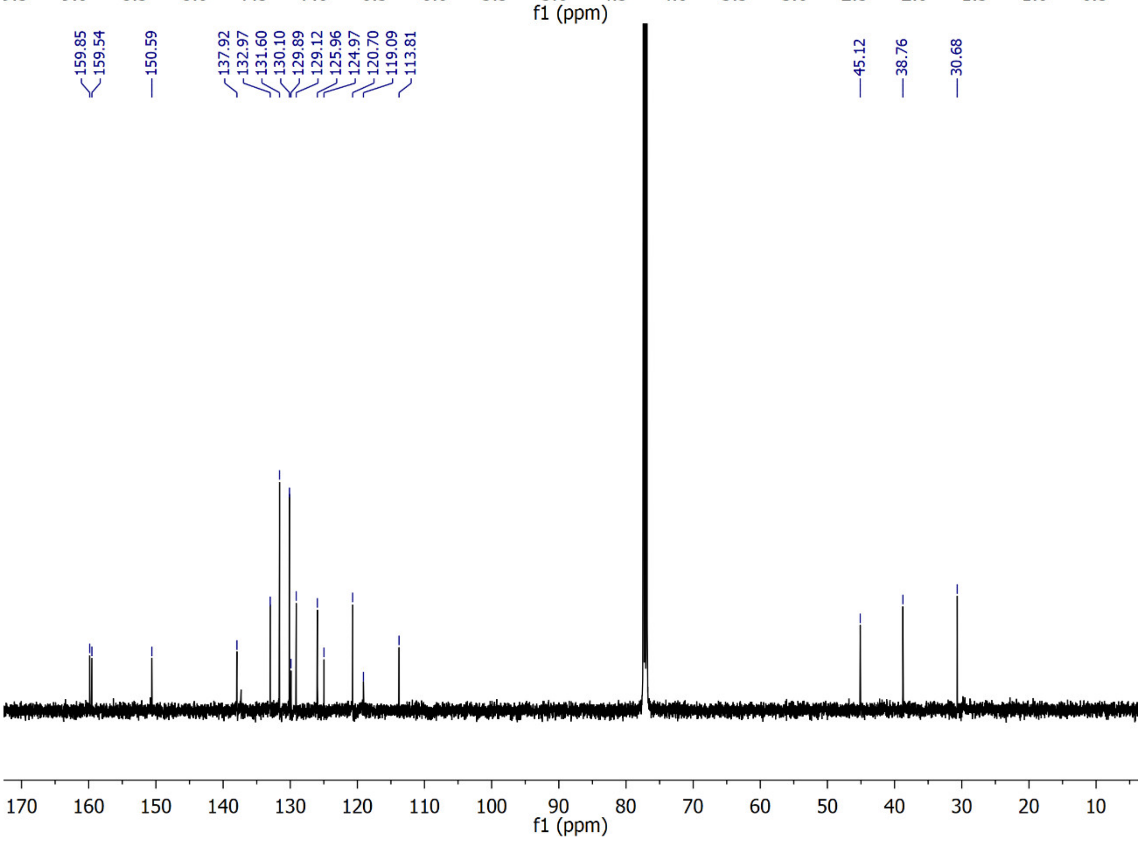
159.85
159.54
150.59
137.92
132.97
131.60
130.10
129.89
129.12
125.96
124.97
120.70
119.09
113.81
45.12
38.76
30.68
f1 (ppm)

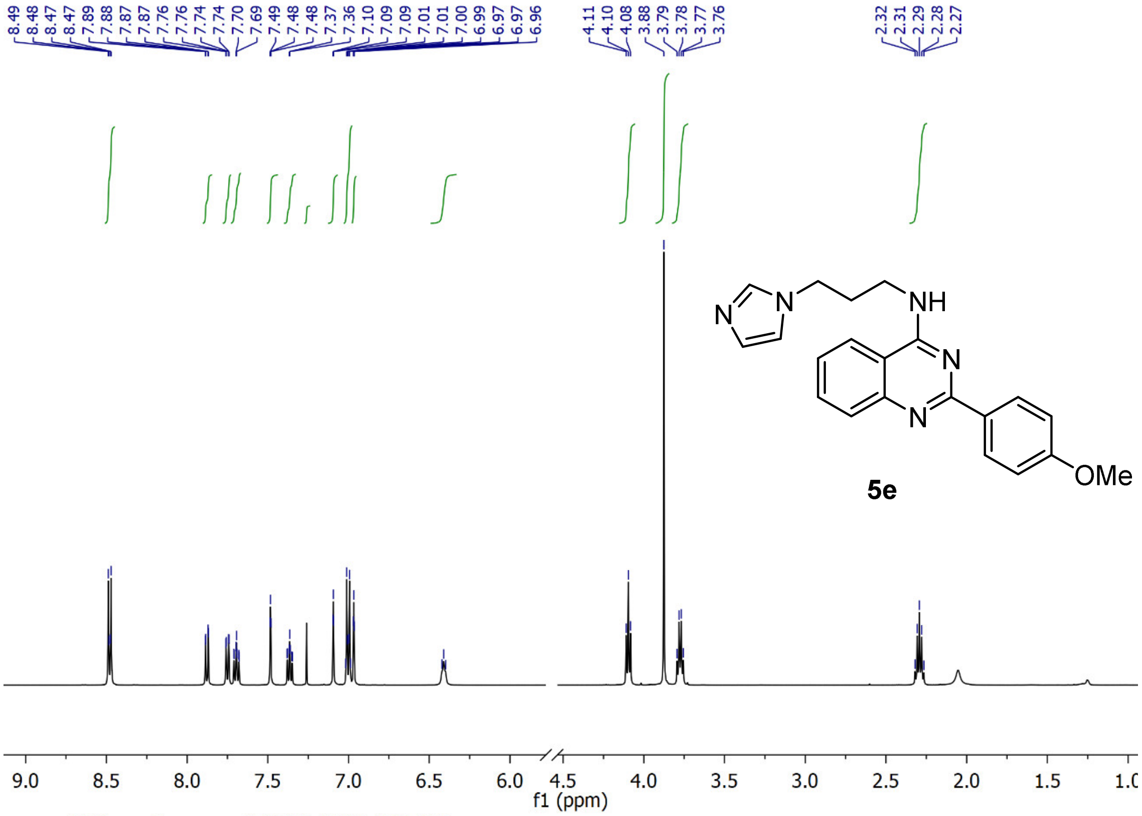
8.49 8.48 8.47 8.47 7.89 7.88 7.87 7.87 7.76 7.76 7.74 7.74 7.70 7.69 7.49 7.48 7.48 7.37 7.36 7.10 7.09 7.09 7.01 7.01 7.00 6.99 6.97 6.97 6.96
4.11 4.10 4.08 3.88 3.79 3.78 3.77 3.76
2.32 2.31 2.29 2.28 2.27
NH
N
N
N
N
OMe
5e
9.0 8.5 8.0 7.5 7.0 6.5 6.0 4.5 4.0 3.5 3.0 2.5 2.0 1.5
f1 (ppm)

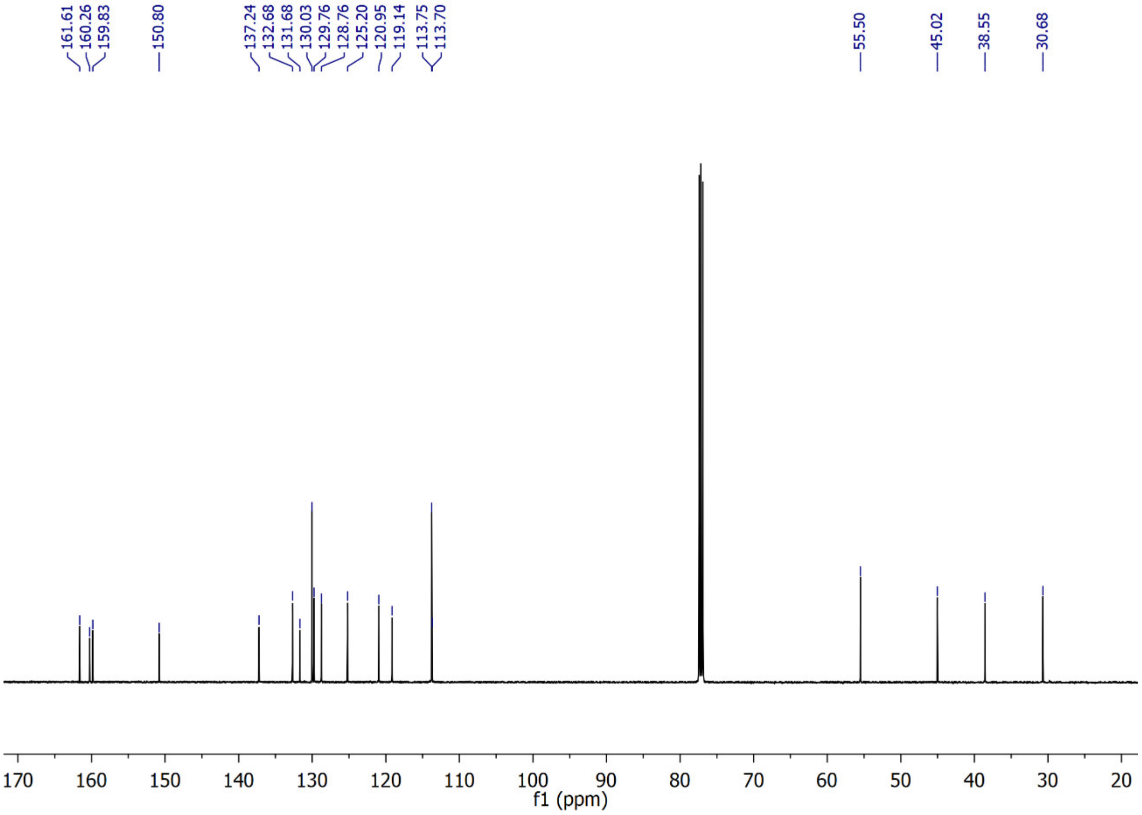
161.61 160.26 159.83 150.80 137.24 132.68 131.68 130.03 129.76 128.76 125.20 120.95 119.14 113.75 113.70
55.50 45.02 38.55 30.68
170 160 150 140 130 120 110 100 90 80 70 60 50 40 30 20
f1 (ppm)

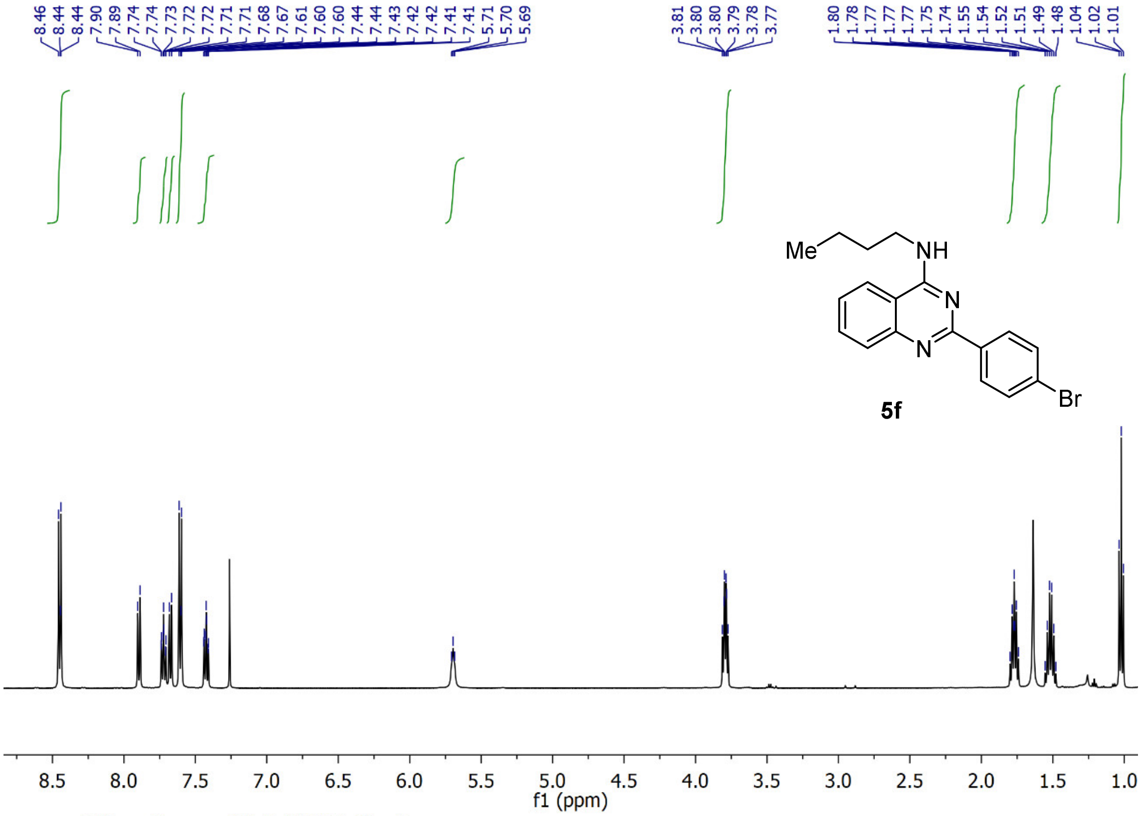
8.46 8.44 8.44 7.90 7.89 7.74 7.74 7.73 7.72 7.72 7.71 7.71 7.68 7.67 7.61 7.60 7.60 7.44 7.44 7.43 7.42 7.42 7.41 7.41 5.71 5.70 5.69
3.81 3.80 3.80 3.79 3.78 3.77
1.80 1.78 1.77 1.77 1.77 1.75 1.74 1.55 1.54 1.52 1.51 1.49 1.48 1.04 1.02 1.01
Me
NH
N
N
Br
5f
8.5 8.0 7.5 7.0 6.5 6.0 5.5 5.0 4.5 4.0 3.5 3.0 2.5 2.0 1.5 1.0
f1 (ppm)

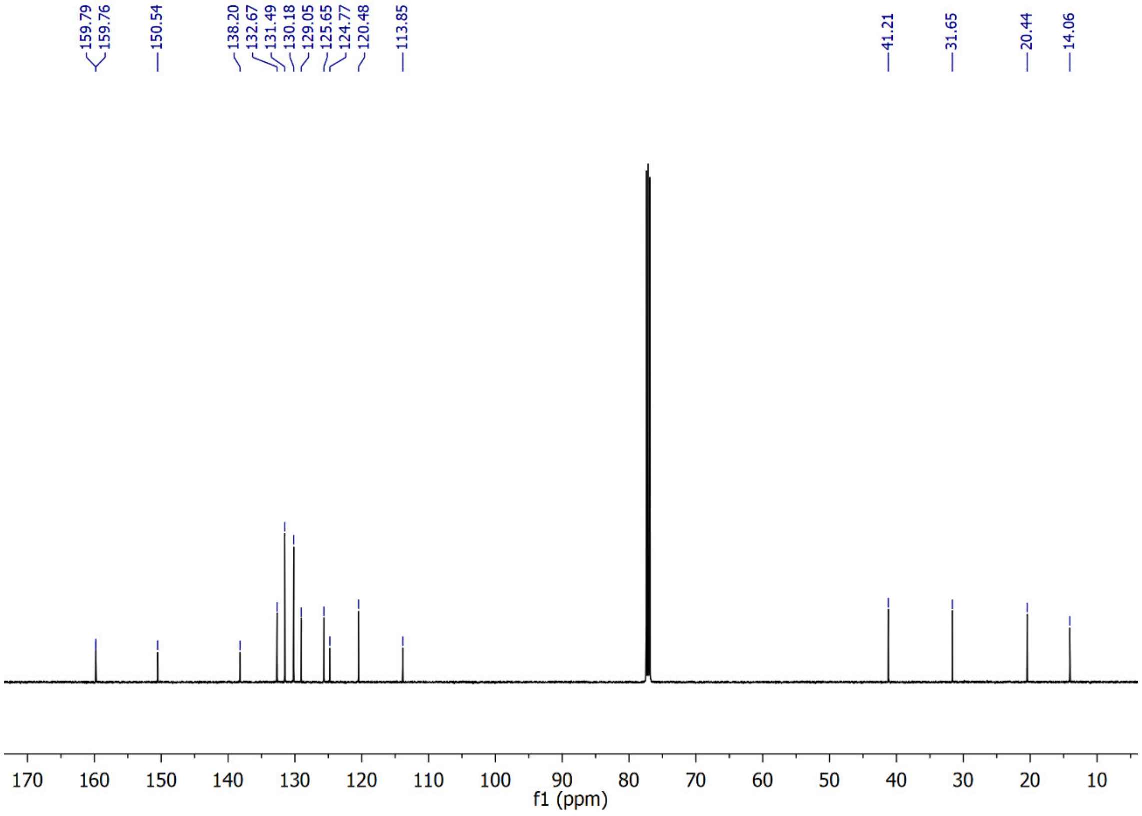
159.79 159.76
150.54
138.20 132.67 131.49 130.18 129.05 125.65 124.77 120.48
113.85
41.21
31.65
20.44
14.06
170 160 150 140 130 120 110 100 90 80 70 60 50 40 30 20 10
f1 (ppm)
